# Synthesis, structure, and properties of switchable cross-conjugated 1,4-diaryl-1,3-butadiynes based on 1,8-bis(dimethylamino)naphthalene

**DOI:** 10.3762/bjoc.19.49

**Published:** 2023-05-15

**Authors:** Semyon V Tsybulin, Ekaterina A Filatova, Alexander F Pozharskii, Valery A Ozeryanskii, Anna V Gulevskaya

**Affiliations:** 1 Department of Chemistry, Southern Federal University, Zorge str. 7, Rostov-on-Don 344090, Russian Federation

**Keywords:** 1,8-bis(dimethylamino)naphthalene, cross-conjugated systems, 1,4-diaryl-1,3-butadiynes, donor–acceptor systems, Glaser–Hay reaction

## Abstract

A set of novel 1,4-diaryl-1,3-butadiynes terminated by two 7-(arylethynyl)-1,8-bis(dimethylamino)naphthalene fragments was prepared via the Glaser–Hay oxidative dimerization of 2-ethynyl-7-(arylethynyl)-1,8-bis(dimethylamino)naphthalenes. The oligomers synthesized in this way are cross-conjugated systems, in which two conjugation pathways are possible: π-conjugation of 1,8-bis(dimethylamino)naphthalene (DMAN) fragments through a butadiyne linker and a donor–acceptor aryl–C≡C–DMAN conjugation path. The conjugation path can be “switched” simply by protonation of DMAN fragments. X-ray diffraction, UV–vis spectroscopy and cyclic voltammetry are applied to analyze the extent of π-conjugation and the efficiency of particular donor–acceptor conjugation path in these new compounds. X-ray structures and absorption spectra of doubly protonated tetrafluoroborate salts of the oligomers are also discussed.

## Introduction

π-Conjugated oligomers and polymers attracted considerable attention from the very start as a promising class of semiconductors, chemosensors, and various electronic devices [1–2]. Although silicon and inorganic materials still play a major role in the development of modern electronics, the prospects for using organic electronic materials as an alternative are becoming increasingly clear. One of the advantages of those materials is a possibility to fine-tune useful properties by simply varying of the π-conjugated backbone and side-chain substituents [1–5].

π-Conjugated oligomers consisting of alternating C≡C bonds and aromatic nuclei, commonly, have a rigid, rod-like structure and exhibit high charge carriers’ mobility [6]. 1,4-Diaryl-1,3-butadiynes is a particular class of such compounds. Both theoretical and experimental studies revealed that the side groups of 1,4-diaryl-1,3-butadiynes have a significant impact on their useful characteristics [7–14]. For example, single-molecule conductivity, nonlinear optical properties, and the ability to serve as photosensitizers of singlet oxygen production have been identified in porphyrin-based butadiynes [7–9], 1,3-butadiyne-linked oligoporphycenes [10], and 1,3-butadiyne-linked amines [13]. A wide variety of applications was proposed for graphdiynes (2D allotropes of graphene), including electrocatalysts and energy devices, which exploit the carbon-rich nature, porous framework, and expanded π-electron system of these compounds [11]. And this is not a complete list.

**Figure 1:**
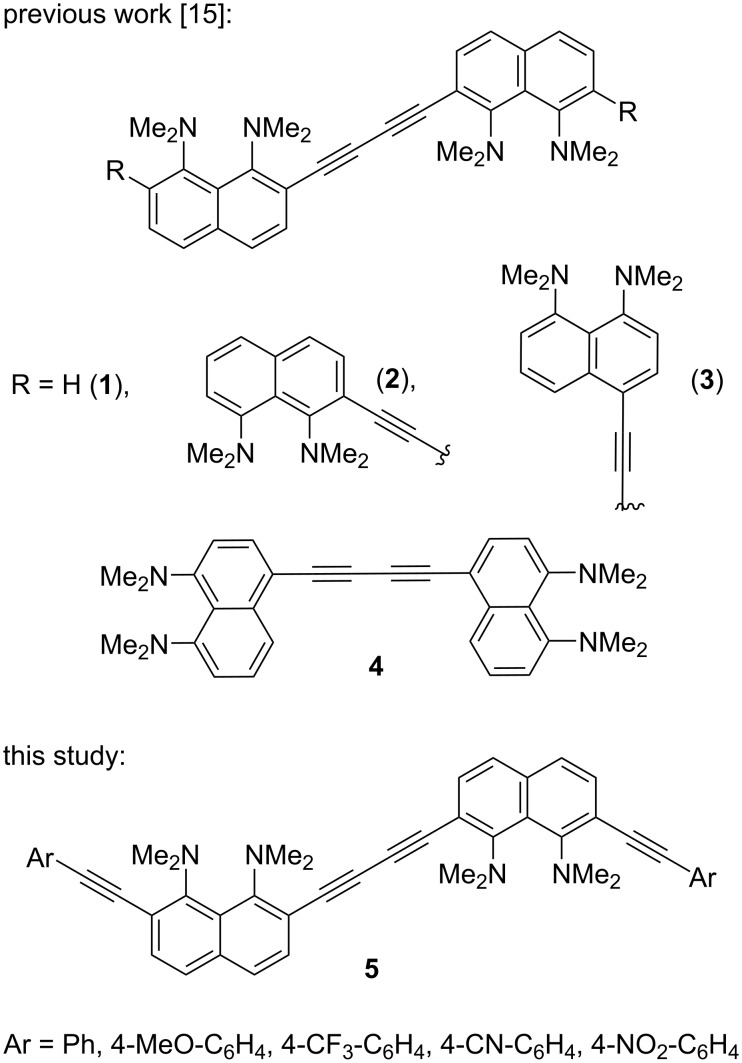
Proton sponge-based 1,4-diaryl-1,3-butadiynes synthesized previously and in this study.

**Figure 2:**
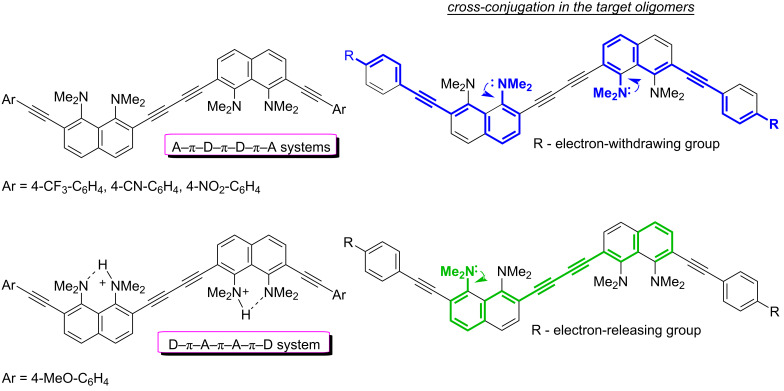
Target oligomers as push–pull and cross-conjugated π-systems.

Recently, we reported on the synthesis of 1,4-diaryl-1,3-butadiynes **1**–**4** based on the “proton sponge” [1,8-bis(dimethylamino)naphthalene, DMAN] (Figure 1) [15]. In the present work we describe the synthesis of a new family of proton sponge-based butadiynes **5** bearing arylethynyl substituents of different electronic nature. Oligomers **5** having electron-withdrawing groups on the aryl termini are interesting as push–pull A–π–D–π–D–π–A systems, whereas the counterpart with an electron-donating methoxy group can be converted into a D–π–A–π–A–π–D system by protonation of the proton sponge fragments (Figure 2). Moreover, oligomers **5** are cross-conjugated π-systems. “A cross-conjugated compound may be defined as a compound possessing three unsaturated groups, two of which although conjugated to a third unsaturated center are not conjugated to each other” [16]. It is easy to see that there are two π-conjugation paths in molecules **5**: a donor–acceptor conjugation path (Figure 2, highlighted in blue) and the π-conjugation of naphthalene rings through a butadiyne linker (highlighted in green). In comparison to linearly conjugated materials, oligomeric and polymeric compounds with a fully cross-conjugated carbon backbone are relatively unexplored [17–20]. Molecules of this type serve not only as objects of fundamental research into the phenomena of cross-conjugation, electron transfer, and quantum interference [17–20], but are also considered as promising molecular switches and transistors [21–25], NLO materials [26–29], and suitable starting compounds for syntheses involving multiple Diels–Alder additions [30]. All these facts motivated us to undertake the current study. X-ray crystallography, UV–vis spectroscopy and cyclic voltammetry were applied to analyze the extent of π-electron conjugation and the efficiency of the particular donor–acceptor conjugation path in chromophores **5**.

## Results and Discussion

### Synthesis

**Scheme 1:**
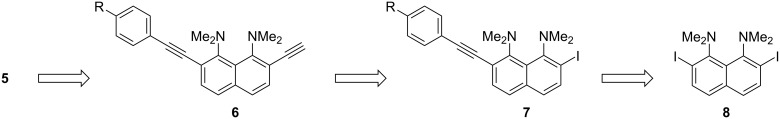
Synthetic strategy for target oligomers **5**.

The target oligomers **5** can be synthesized by a Glaser oxidative dimerization of monomers **6** (Scheme 1). The obvious route for the synthesis of the latter is the sequential alkynylation of 2,7-diiodonaphthalene **8**.

In accordance with this strategy, diiodide **8** was cross-coupled with copper(I) arylacetylides (Castro–Stephens reaction, method A) and arylacetylenes (Sonogashira reaction, method B). In all cases, even when using a small excess of **8**, in addition to the desired monoalkynyl derivative **7**, a double alkynylation product **9** was formed (Table 1). The Sonogashira coupling was somewhat more efficient, yielding compounds **7a–e** in 42–62% yields, but also gave higher amounts of products **9a–e** (10–30%). Thus, the Pd- and phosphine-free Castro–Stephens coupling was a good enough alternative to synthesize alkynes **7**. The structure of the double alkynylation product **9e** was confirmed by X-ray diffraction data (see Supporting Information, File 1, Figure S60).

**Table 1:** Synthesis of 7-(arylethynyl)-2-iodo-DMAN **7**.

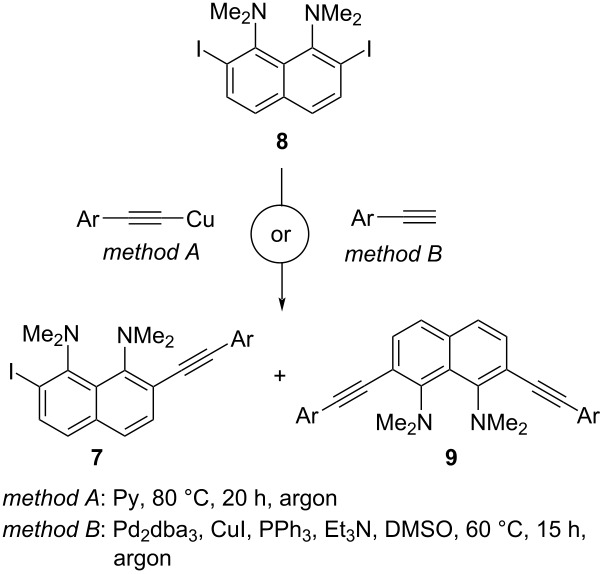					

Ar		Yield, %			

		method А		method B	

		**7**	**9**	**7**	**9**

Ph	**a**	52	18	62	30
4-MeO-C_6_H_4_	**b**	41	2.5	51	21
4-CF_3_-C_6_H_4_	**c**	45	11	53	22
4-CN-C_6_H_4_	**d**	24	9	49	26
4-NO_2_-C_6_H_4_	**e**	30	5	42	10

**Scheme 2:**
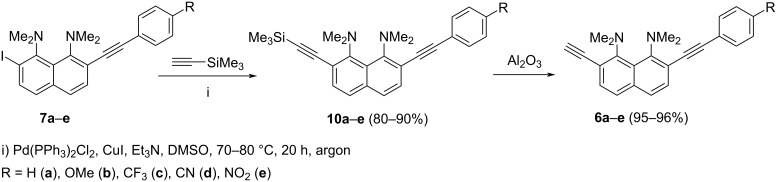
Synthesis of 7-(arylethynyl)-2-ethynyl-DMAN **6**.

The further alkynylation of compounds **7a**–**e** was carried out using trimethylsilylacetylene and the Pd(PPh_3_)_2_Cl_2_/CuI/Et_3_N/DMSO catalytic system giving rise to dialkynyl derivatives **10a**–**e** in high yields (Scheme 2). Column chromatography of trimethylsilyl derivatives **10a**–**e** on Al_2_O_3_ resulted in their quantitative desilylation with the formation of the target monomers **6a**–**e**, thus eliminating the need to remove the trimethylsilyl protection. Pure samples of compounds **10a**–**e** could be obtained by extraction of the reaction mixture with hexane followed by recrystallization of the crude product from ethanol.

**Scheme 3:**
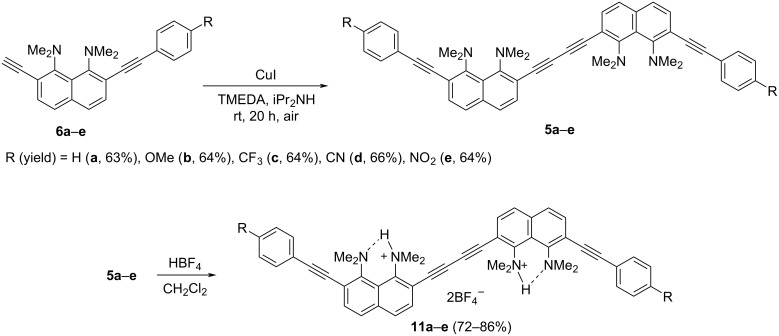
Synthesis of 1,4-diaryl-1,3-butadiynes **5** and their salts **11**.

Next, the oxidative dimerization of terminal alkynes **6a**–**e** was carried out in an aerobic medium in the CuI/TMEDA/iPr_2_NH system at room temperature, which proved to be effective in the synthesis of butadiynes **1**–**4** [15] (Scheme 3). The desired diarylbutadiynes **5a**–**e** were obtained in good yields regardless of the substituent R in the benzene ring. Treatment of the latter with fluoroboric acid in dichloromethane gave double salts **11a**–**e**.

### X-ray structures

**Figure 3:**
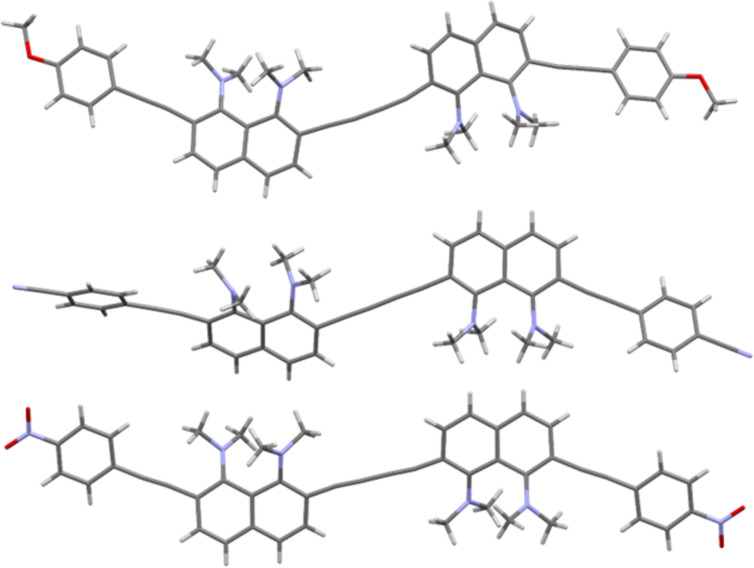
Molecular structures of compounds **5b** (top), **5d** (middle), and **5e** (bottom).

**Figure 4:**
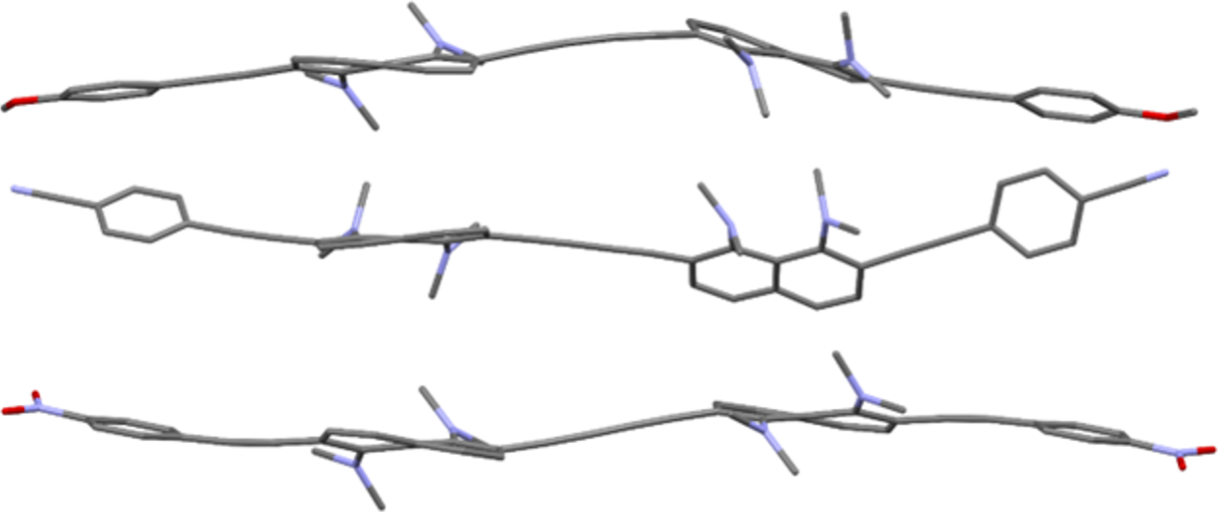
Views on the molecular backbone of compounds **5b** (top), **5d** (middle), and **5e** (bottom) along the naphthalene rings plane (hydrogen atoms omitted).

**Scheme 4:**
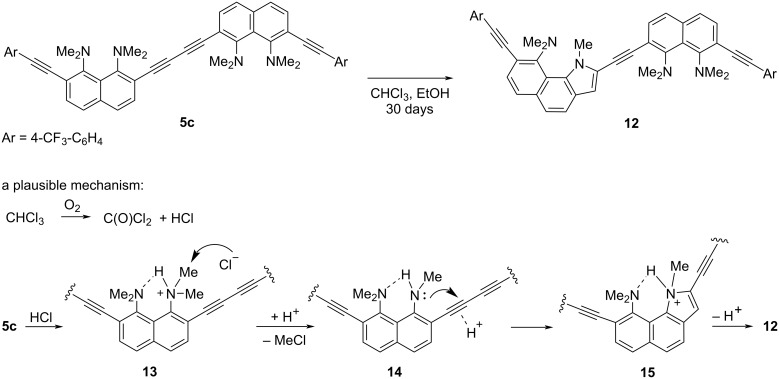
Transformation of butadiyne **5c** into benzo[*g*]indole **12**.

**Figure 5:**
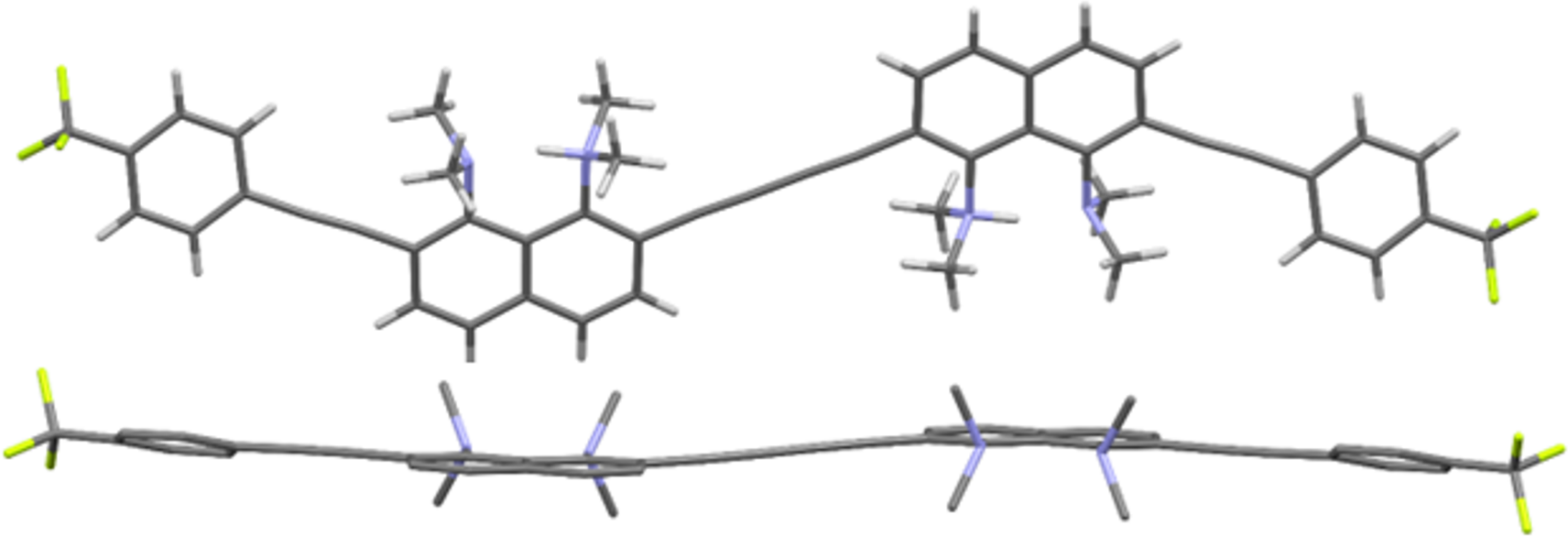
Molecular structure of compound **11c**: frontal (top; BF_4_^−^ omitted) and side views (bottom; hydrogen atoms omitted).

Slow evaporation of solutions of butadiynes **5** in the CHCl_3_/EtOAc system made it possible to grow single crystals of samples **5b**, **5d**, and **5e** suitable for X-ray diffraction studies (Figure 3 and Figure 4). Crystals of compound **5c** were grown up using CHCl_3_/EtOH solvent, and it was unexpectedly found that keeping this compound in the above system for a month leads to its partial heterocyclization to benzo[*g*]indole **12** (Scheme 4 and Figure S59 in Supporting Information File 1). The structure of compound **12** was unambiguously established by X-ray diffraction analysis (see Supporting Information File 1, Figure S61). We assumed that this transformation is facilitated by hydrogen chloride, which is formed during the oxidation of chloroform with atmospheric oxygen. We also succeeded in growing crystals of the salt **11c** in the MeCN/EtOH system (Figure 5). Unfortunately, good crystals of other salts have not been obtained.

From Figure 3 and Figure 4 it is easy to see that all molecules **5b**, **5d**, and **5e** are rather distorted, including naphthalene cores, butadiyne and acetylene linkers. The main structural parameters of diynes **5** that characterize the degree of this distortion are presented in Table 2, where ϕ1 is the angle between the planes of the benzene ring and the neighboring naphthalene system, ϕ2 is the angle between the averaged planes of the naphthalene rings, ∠Cx‒Cy–Cz is the bond angle of the carbon–carbon bonds in the butadiyne linker, Θ is the C2(2′)–C3(3′)–C6(6′)–C7(7′) torsion, N···N is the internitrogen distance in the DMAN fragments, Σ∠N is the sum of the C–N–C angles of the NMe_2_ groups, and φ is the N1(1′)–C1(1′)–C8(8′)–N8(8′) torsion. For salt **11c**, two additional parameters characterizing the hydrogen N–H···N bond are given, e.g., the N–H bond lengths and the angle between them (∠N–H···N).

**Table 2:** Some structural parameters of oligomers **5** and salt **11c** (X-ray data).

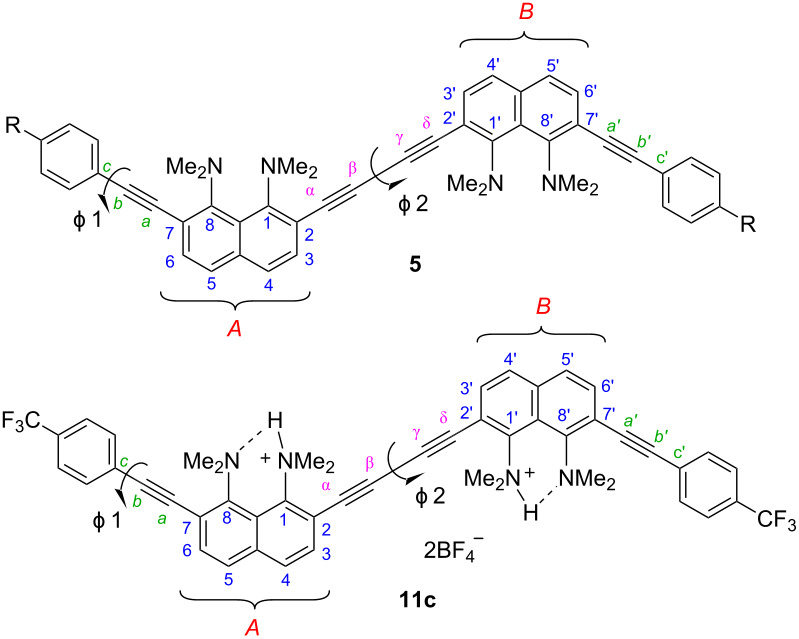								

Parameter		**5b** (R = OMe)		**5d** (R = CN)		**5e** (R = NO_2_)	**11c** ^a^	
		
		*A*	*B*	*A*	*B*	*A = B*	*A = B*	

ϕ2 torsion, °		17.4		34.4		0	0	0
ϕ1 torsion, °		1.4	12.7	28.2	29.0	17.9	32.4	12.8
∠C2‒Cα‒Сβ, ° ∠Cα‒Cβ‒Сγ, ° ∠Cβ‒Cγ‒Сδ, ° ∠Cγ‒Cδ‒С2′, °		176.9 172.9 176.0 170.2		177.5 177.7 177.9 176.7		172.2 177.6	173.5 178.2	176.1 177.7
∠C7(7')‒Ca(a')‒Сb(b'), ° ∠C*a*(*a'*)‒C*b*(*b'*)‒С*c*(*c'*), °		173.2 178.3	173.3 179.1	174.1 178.5	174.5 176.9	174.5 172.2	168.5 174.2	175.0 176.2
C2(2′)–C3(3′)–C6(6′)–C7(7′) torsion Θ, °		19.6	15.3	12.8	9.5	18.6	0.8	0.0
N···N distance, Å		2.859	2.808	2.779	2.772	2.848	2.553	2.570
sum of the C–N–C angles Σ∠N, °	N1(1′)	357.3	357.6	354.4	354.9	357.6	340.0	340.5
	N8(8′)	359.3	357.0	357.3	353.5	358.6	341.0	340.9
N1(1′)–C1(1′)–C8(8′)–N8(8′) torsion φ, °		29.0	21.7	20.9	8.4	31.6	2.7	2.5
N–H···N bond lengths, Å		–	–	–	–	–	1.03 1.54	1.01 1.59
∠N–H···N, °		–	–	–	–	–	166	162

^a^Structural parameters of two independent molecules are given.

In all cases naphthalene fragments linked by a 1,3-butadiyne axis take a *trans* position relative to each other. Despite formally symmetrical structure of diynes **5**, naphthalene rings *A* and *B* of molecules **5b** and **5d** differ in their structural parameters. At the same time, the monomer fragments of the nitro derivative **5e** are identical. In the case of **5e**, the naphthalene rings lie in parallel planes, while in crystals of **5b** and **5d** the angle between the average planes of naphthalene nuclei *A* and *B* reaches 17° and 34°, respectively. Molecule **5d** demonstrates the largest rotation angles of the aryl termini with respect to the naphthalene rings (ϕ1 = 28–29°). The dimethylamino groups of **5** are strongly flattened (the Σ∠N value varies from 353.5° to 359.3°), which is characteristic of *ortho*-substituted proton sponges [31]. The observed N···N distances are slightly larger than those typical for *ortho*-disubstituted DMAN derivatives [31].

Molecule **5b** is the most distorted, as evidenced by the significant twisting of naphthalene rings (Θ_A_ = 19.6° and Θ_B_ = 15.3°), the largest internitrogen distance (2.859 and 2.808 Å for rings *A* and *B*, respectively), the largest deviation of the dimethylamino groups from the naphthalene ring plane (φ_A_ = 29.0°, φ_B_ = 21.7°), as well as deviation of the bond angles of the butadiyne linker from the standard value of 180° by 3–10°. Deviations of bond angles in acetylene bridges are ≈1–7°. The methoxy derivative **5b** has the most complex crystal packing with a large number of different nonvalent interactions (see Supporting Information File 1, Figures S62 and S63).

The molecule of cyano derivative **5d** is characterized by the least distortion of the DMAN fragments in the series (twisting Θ = 9.45 and 12.83°, torsions φ_A_ = 20.9° and φ_B_ = 8.4°, bond angle deviations in both butadiyne and acetylene linkers do not exceed 6°). In the crystal packing of **5d** (see Supporting Information File 1, Figures S64 and S65), the DMAN fragments do not participate in nonvalent interactions and do not form short contacts. The recurring motif in the crystals is the coordination of the benzene *meta* proton by the nitrogen atom of the C≡N group.

The structural parameters of both monomer fragments of nitro derivative **5e** are identical. This fact, together with the parallelism of the naphthalene ring planes, indicates the existence of an inversion center in the molecule. Molecule **5e** is characterized by the largest N1(1′)–C1(1′)–C8(8′)–N8(8′) torsion angle φ (31.6°) in the series. Other structural parameters are close to those of the molecule **5b**. In the crystal packing (Figure S66 in Supporting Information File 1), molecules **5e** tend to approach π-donor DMAN and π-acceptor *p*-nitrophenyl fragments, and the shortest distance between the two molecules is 2.810 Å (Figure S67 in Supporting Information File 1).

**Figure 6:**
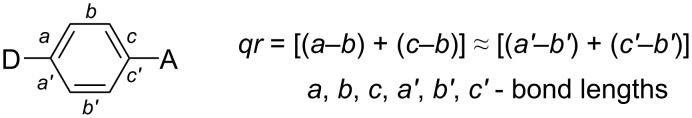
Calculation of the *qr* parameter.

The alternation of the C–C bond lengths in the aryl rings of molecules **5d** and **5e** may indirectly indicate the conjugation of the π-donor fragment with the π-acceptor *p*-nitrophenyl or *p*-cyanophenyl fragments. The *qr* parameter, calculated according to equation [32] (Figure 6) and characterizing the quinoid character of the aryl ring, was proposed for D–π–A systems. This parameter is a good indication for intramolecular charge transfer from the donor to the acceptor moiety in the ground state. In benzene, the *qr* value is equal to 0. In a fully quinoid ring, the *qr* was found to be equal to 0.10–0.12.

Calculations based on the bond lengths in the aryl fragments determined by X-ray diffraction analysis gave the following average values of the *qr* parameter: 0.012 for **5d** and 0.014 for **5e**. For comparison, the same parameter calculated for *N*,*N*-dimethyl-4-nitroaniline is 0.038 (X-ray data from reference [33]). Therefore, the π-charge transfer from the donor DMAN to the acceptor aryl ring of **5d** and **5e** is extremely modest in the ground state. It should be also noted that the C_Naph_–N bonds of **5e** are the shortest in the series (1.379–1.380 Å), which may also indirectly indicate a more pronounced conjugation of the dimethylamino groups with the nitro group.

There are two types of independent non-equivalent dications, marked in blue and green, and two types of BF_4_^−^ anions, marked in red and yellow, in the crystal structure of salt **11c** (Figure S68 in Supporting Information File 1). Monomer fragments in both are identical (Table 2, Figure 5). The trifluoromethyl group of one independent molecule is disordered with an occupancy of fluorine atoms of 0.54/0.46, which makes the molecule asymmetric. The second independent molecule has an inversion center. Compared to the free bases **5** discussed above, the protonated form **11с** demonstrates almost complete planarization of the naphthalene backbone due to the disappearance of steric and electrostatic stress between the NMe_2_ groups. The nitrogen atoms in the DMAN fragments strongly approach during protonation and practically do not deviate from the average naphthalene ring plane. The dimethylamino groups naturally become more pyramidal and the C_Naph_–N bonds lengthen. All these changes are typical for protonated DMAN derivatives [31]. Interestingly, the NH protons are localized on the 1-NMe_2_ and 1′-NMe_2_ groups adjacent to the butadiyne linker and do not move away from each other at the maximum distance closer to the aryl substituents. Noteworthy is the bending of the acetylene linker (in one of the independent molecules, the bond angle is only 168.5°) and the greater linearity of the butadiyne fragment, which may indicate a more pronounced conjugation between two DMAN fragments than between DMAN and *p*-trifluoromethylphenyl rings. By the way, the *qr* parameters calculated for two independent molecules of **11c** were 0.008 and 0.009, which is slightly less than in the case of compounds **5**. As for the crystal packing of **11c**, BF_4_^−^ anions of two types (“red” and “yellow”) interact with cations in different ways. The “red” anion hangs over the cationic centers of both independent molecules, which are almost perpendicular to each other, while the “yellow” one participates mainly in the coordination with the hydrogen atoms of the NMe_2_ groups. Such a distribution of counterions apparently ensures mutually perpendicular packing of almost linear molecules (Figure S69, Supporting Information File 1).

### UV–vis spectra and redox properties

**Figure 7:**
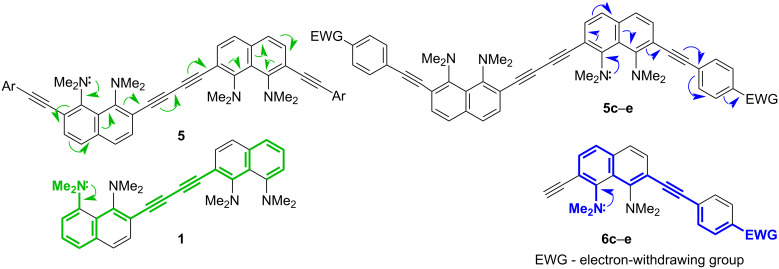
Two π-conjugation ways in oligomers **5**.

**Figure 8:**
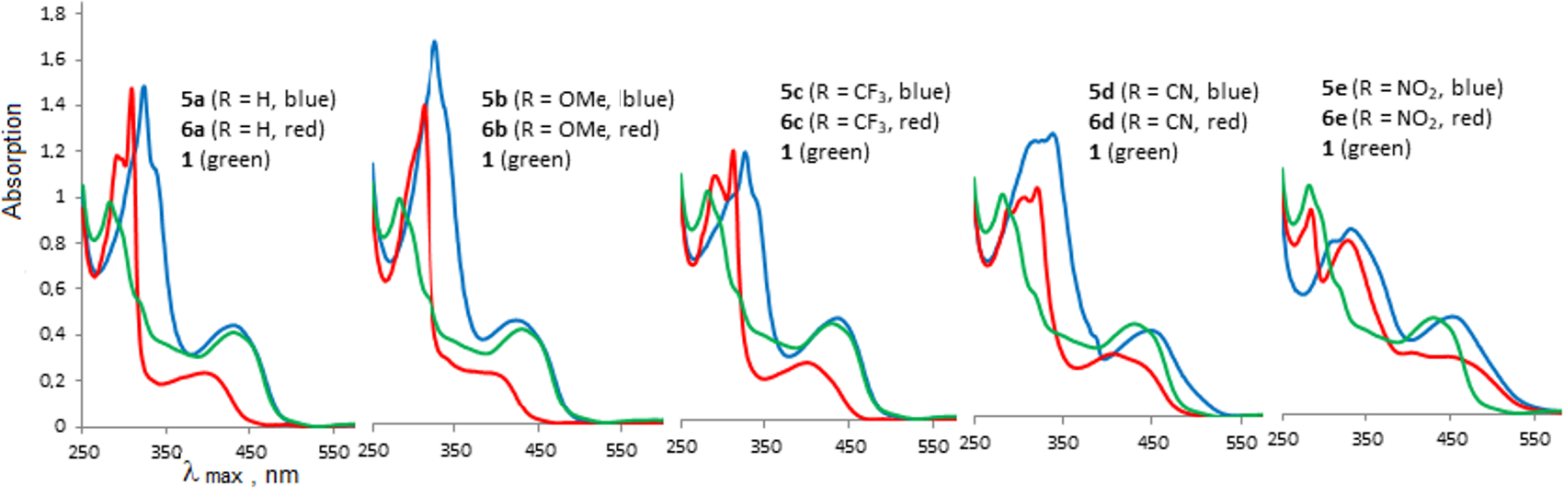
UV–vis spectra of oligomers **5** (blue line), monomers **6** (red line), and butadiyne **1** (green line).

As stated above, oligomers **5** are cross-conjugated π-systems. For cross-conjugated structures, the main question is about the preferential conjugation path. For oligomers **5**, two different directions of electron density transfer are possible (Figure 7): between two DMAN fragments through the butadiyne linker (highlighted in green) and between the DMAN and aryl rings through the acetylene bridge (highlighted in blue). Obviously, the “butadiyne path” includes a longer conjugation chain. Noncovalent interactions of molecules in crystals and packing effects do not allow one to strictly judge the charge transfer in the oligomers **5**. Therefore, we analyzed their UV–vis spectra (Table 3, Figure 8). The functional groups R are located at the far ends of the oligomeric chain and, from the steric point of view, cannot have a significant effect on conformational transformations of **5**. All differences in optical properties must be of an electronic nature. It is obvious that the same “butadiyne conjugation pathway” (marked in green) is realized in compound **1**, while the conjugation chain in monomers **6** is identical to the “blue” one in oligomers **5** (Figure 7). Thus, the UV–vis spectra of compounds **1** and **6** were used for comparison (Table 3, Figure 8).

**Table 3:** Summary of the UV–vis spectra^a^ of monomers **6**, oligomers **5** (in CHCl_3_), and salts **11** (in MeCN).

R		Monomer **6**	Oligomer **5**			Salt **11**		

		λ_max_, nm (lg ε)	λ_max_, nm (lg ε)	λ_onset_, nm	*E*_g_^opt^, eV^b^	λ_max_, nm (lg ε)	λ_onset_, nm	*E*_g_^opt^, eV^b^

H	**a**	393 (3.98)	432 (4.51)	519	2.39	382 sh (4.28)	397	3.12
OMe	**b**	405 sh (3.92)	423 (4.54)	518	2.39	387 sh (4.31)	415	2.99
CF_3_	**c**	400 (4.08)	437 (4.64)	524	2.37	382 sh (4.30)	390	3.18
CN	**d**	441 sh (4.08)	449 (4.54)	547	2.27	383 sh (4.24)	392	3.16
NO_2_	**e**	456 sh (3.92)	453 (4.58)	594	2.09	385 sh (4.19)	396	3.13

^a^Absorption maxima measured in the corresponding solutions at *c* = 10^−5^ M. ^b^The optical gap estimated from the onset point of the absorption spectra: *E*_g_^opt^ = 1240/λ_onset_.

The long-wave absorption maximum of the yellow-colored butadiyne **1** is observed at 429 nm (lg ε = 4.33) [15]. Compounds **5a**‒**с** (R = H, OMe, CF_3_) are yellow, **5d** (R = CN) is orange, and **5e** (R = NO_2_) is a crimson crystalline substance. As can be seen from Table 3, compound **5b** with electron-releasing methoxy groups shows the smallest λ_max_ value, while the absorption maximum of the nitro derivative **5e** is the most red-shifted in the series. There is a noticeable difference in the absorption maxima of diynes **5a**–**c** and the corresponding monomers **6a**–**c**. Moreover, the absorption maxima of compounds **5a** (R = H), **5b** (R = OMe), and **5c** (R = CF_3_) are rather close to diyne **1**. Figure 8 clearly demonstrates that in these cases the profiles of the long-wavelength maximum almost overlap with those of butadiyne **1**. Apparently, in molecules **5a**–**c**, the “butadiyne conjugation pathway” is realized, involving a larger number of multiple bonds. The absorption maxima of compounds **5a** and **5c** are slightly red-shifted (Δλ 3–8 nm), while in the case of compound **5b** a hypsochromic shift of λ_max_ is observed. On passing to oligomers **5d** and **5e** bearing strong electron-withdrawing CN and NO_2_ substituents in the benzene rings the picture changes. The π-deficient nature of the terminal aryl rings obviously facilitates their conjugation with π-excessive DMAN fragments. The λ_max_ values and the general shape of the spectra of compounds **5d** and **5e** are closer to those of the corresponding monomers **6d** and **6e**, but not of butadiyne **1**. The absorption maximum of derivative **5d** is red-shifted by 8 nm relative to monomer **6d**.

It should be noted that oligomers **5d** and **5e** as well as the corresponding monomers **6d** and **6e** are typical rod-like D–π–A systems. In such molecules, a photon absorption induces a shift from a D–π–A ground state to a D^+^–π–A^−^ excited state. It is obvious that the actual electron transfer depends on all three components of the push–pull molecule. However, the study on the through-space charge transfer (CT) in the rod-like donor–acceptor molecules showed that adding a stronger electron-donating group does not systematically induce an enhancement of the CT if a strong electron-accepting moiety is used, the latter tending to extract the electron from the conjugated chains rather from the donor moiety [34]. We therefore compared the UV spectra of the oligomers **5d** and **5e** and monomers **6d** and **6e** with those of model *p*,*p*'-disubstituted diphenylacetylenes having donor NMe_2_ and acceptor NO_2_ or CN termini. The reported absorption maxima of 4-((4-(dimethylamino)phenyl)ethynyl)benzonitrile and *N*,*N*-dimethyl-4-((4-nitrophenyl)ethynyl)aniline in chloroform solution are 373 and 416 nm, respectively [35]. In the same time, λ_max_ for 2-((4-nitrophenyl)ethynyl)-1,8-bis(dimethylamino)naphthalene is 474 nm [36]. The red shift observed in the spectrum of this compound as well as in the spectra of compounds **5d**,**e** and **6d**,**e** is likely a reflection of the elongation of the conjugation chain. This also supports the conjugation pathway between the nitro group and the more distant dimethylamino group of DMAN fragment marked in blue in Figure 7.

All synthesized oligomers **5** display no fluorescence in solution (chloroform and acetonitrile were tested). For comparison, *N*,*N*-dimethyl-4-((4-nitrophenyl)ethynyl)aniline demonstrates a weak fluorescence with an emission maximum at 550 nm (EtOH) [37]. The optical band gaps (*E*_g_^opt^), estimated from the onset point of the absorption spectra, ranged within 2.39 eV (for **5a** and **5b**) to 2.09 eV (for **5e**). Thus, the HOMO–LUMO gap is significantly reduced by the introduction of the electron-withdrawing substituent, while the introduction of a donor substituent, e.g., a OMe group, does not change this value.

Previously, using the example of diphenylpolyynes containing a donor *p*-amino and an acceptor *p*’-nitro terminus, it was shown that upon protonation the band associated with the intramolecular charge-transfer transition emanating from the lone pair on the NH_2_ nitrogen and terminating in an empty π* orbital on the NO_2_ group, disappears [38]. The high-energy absorption for these compounds were largely unaffected by HCl protonation, the UV spectra of the protonated forms were very similar to those of unsubstituted diphenylpolyynes attributing the above bands to a π→π* transition. Сomplete protonation of DMAN-based diarylacetylenes led to a hypsochromic shift of their absorption maxima by 40–70 nm. The UV spectra of these salts are similar to those of the corresponding unsubstituted dinaphthylacetylenes [39].

**Figure 9:**
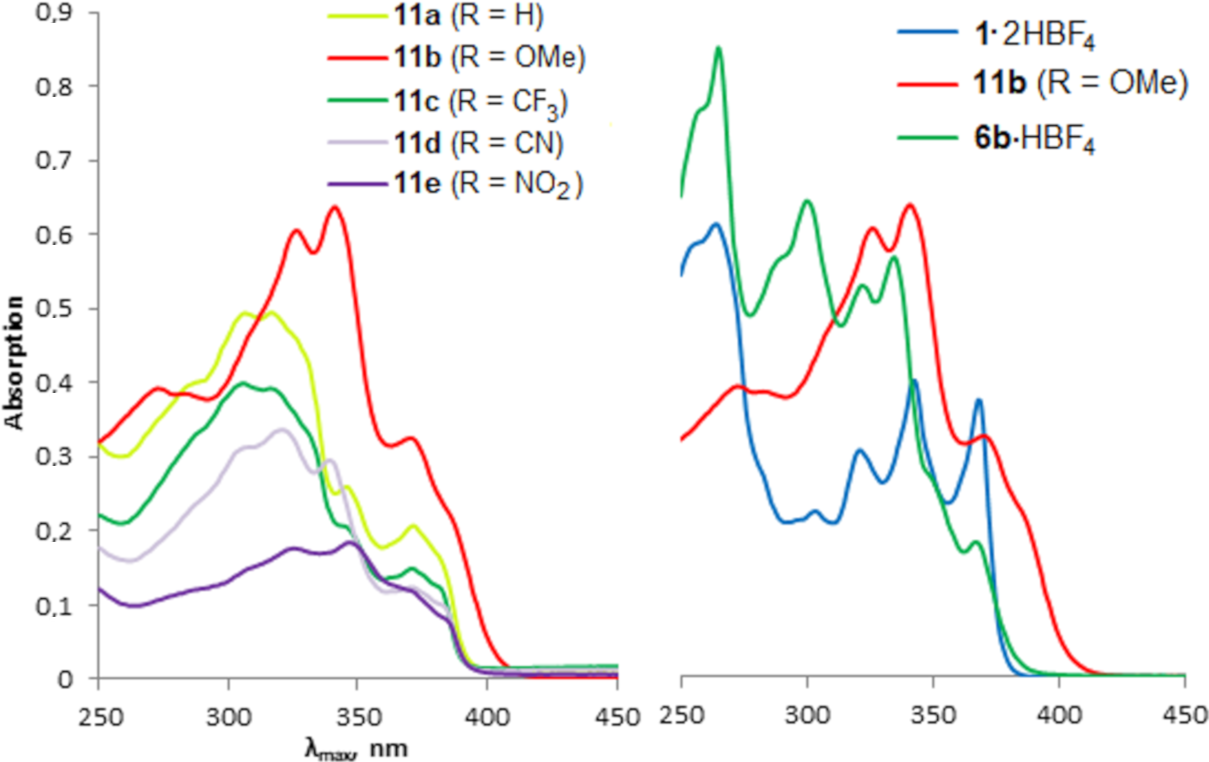
UV–vis spectra of salts **11** (left), **1**·2HBF_4_ and **6b**·HBF_4_ (right) in acetonitrile.

**Figure 10:**
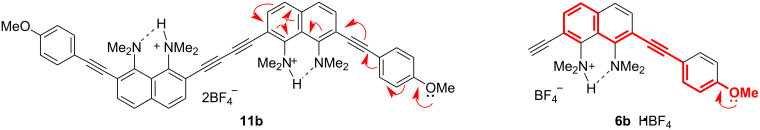
π-Conjugation pathway in salts **11b** and **6b**·HBF_4_.

In cases of oligomers **5** a comparable picture was observed. The protonated oligomers **11** show similar to each other UV–vis spectra and absorption maxima (Table 3, Figure 9). However, unlike salts **11a**,**c**,**d**, methoxy derivative **11b** demonstrates end absorption up to 415 nm and, thus, the lowest optical band gap in the series (2.99 eV). Evidently, protonation of **5b** gives rise to a push–pull D–π–A–π–A–π–D system, in which the π-conjugation between the donating methoxyphenyl and the accepting protonated DMAN fragments becomes preferable (Figure 10). This is supported by a comparison of the absorption spectra of salt **11b** and protonated forms of diyne **1** [15] and monomer **6b** (salts **1**·2HBF_4_ and **6b**·HBF_4_), which demonstrates an obvious similarity of spectral curves of **11b** and **6b**·HBF_4_. Since the UV–vis spectra of salts **11** are similar (even and especially in cases of methoxy and nitro derivatives **11e** and **11b**), it can be assumed that in all salts **11** the electron transfer from the terminal aryl to the central naphthalene rings takes place. Naturally, the more deficient the aryl fragment, the less intense the long-wavelength maximum. Conjugation between the aryl substituent and the DMAN fragment in salts **11** is indirectly supported by the fact that the basicity of monomer **6b** with the donor methoxy group (p*K*_a_ = 8.2, measured in DMSO by the ^1^H NMR transprotonation approach [40]) is almost an order of magnitude higher than that of monomer **6e** with the acceptor nitro group (p*K*_a_ = 7.3).

The absorption maxima of 2,7-dialkynyl derivatives of DMAN **9** were observed at 402–465 nm, regularly shifting to the red region when passing from compound **9b** bearing terminal methoxy groups to its analogs with electron-withdrawing substituents.

**Figure 11:**
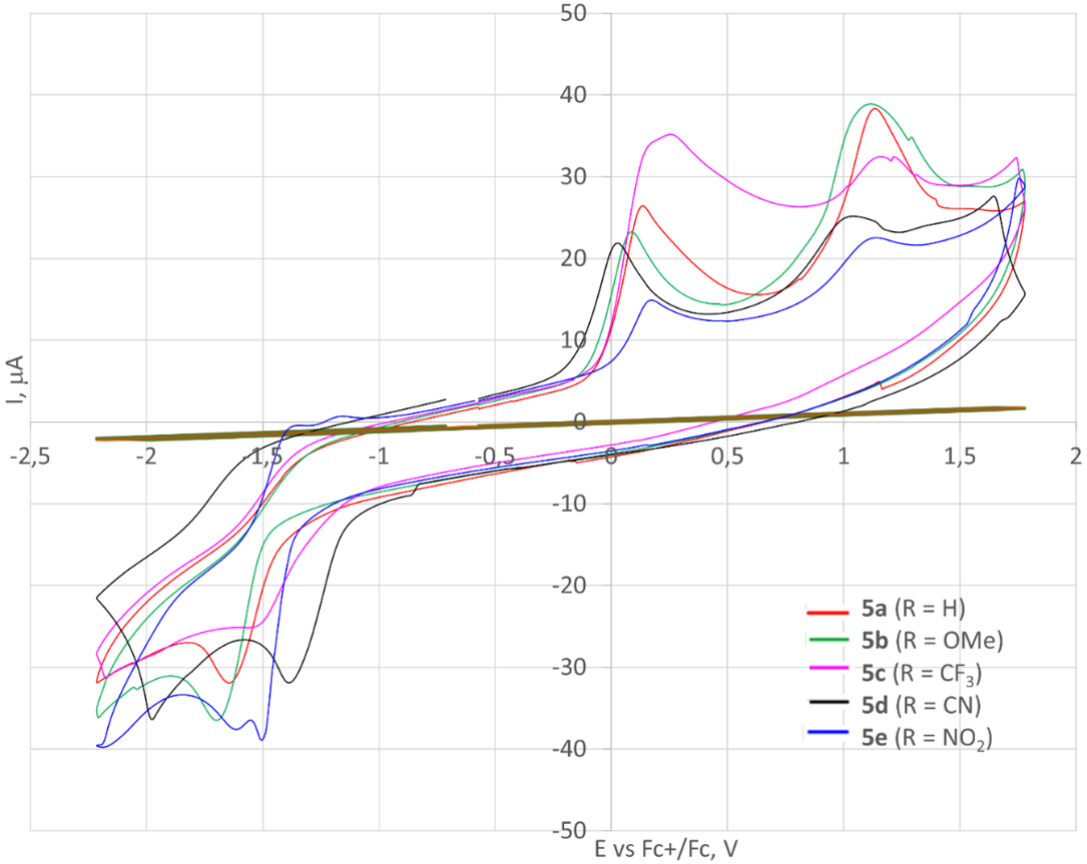
Cyclic voltammograms of oligomers **5**.

**Scheme 5:**
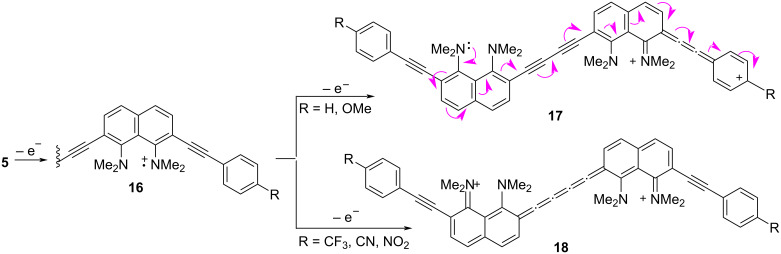
Possible ways of one- and two-electron oxidation of oligomers **5**.

The redox properties of oligomers **5** were evaluated by cyclic voltammetry (CV) in dichloromethane solution containing 0.1 M *n*-Bu_4_NPF_6_ in the standard three-electrode electrochemical cell: glassy carbon working electrode, platinum auxiliary electrode, and reference electrode Ag/Ag^+^ 0.01 M AgNO_3_ (Figure 11, Table 4). Compounds **5a**–**d** displayed two waves of irreversible oxidation in the potentials range of 0.0–1.1 V and one reduction wave (−1.5 to −1.6 V) with the little variation of the potentials induced by the substituent R. The CV curve of nitro derivative **5e** demonstrated the minimum peak current. Considering that the current is a quantitative expression of how fast an electrochemical process is happening, compound **5e** shows the lowest oxidation rate. In this case, two quasi-reversible reduction waves with lower *E*_1/2_^ox^ compared to the other oligomers **5** were observed. Apparently, two nitrophenyl fragments are successively reduced in this process. A distinctive feature of the CV curves of compounds **5a** and **5b** was a more pronounced second oxidation wave. We speculated that the second oxidation of molecules **5** gives dicationic species (Scheme 5). Presumably, when R = H or OMe, structure **17** contributes the most to the resonance hybride, while in cases of molecules **5d**,**e** with π-acceptor substituents, a dication of type **18** better describes the electron density distribution. The only exception is the trifluoromethyl derivative **5с**, which shows the most positive oxidation potential and rate of the first oxidation as well as the lowest rate of reduction. However, the relatively small differences may simply be due to the different local solvation of the CF_3_ substituent.

**Table 4:** Cyclic voltammetry data of oligomers **5** in CH_2_Cl_2_ (+ 0.1 M *n*-Bu_4_NPF_6_).

Compd.	Half-wave potentials			

	*E*_1/2_^ox^ (1), V	*E*_1/2_^ox^ (2), V	*E*_1/2_^red^ (1), V	*E*_1/2_^red^ (2), V

**5a**	0.03	0.98	−1.52	–
**5b**	−0.01	0.95	−1.59	–
**5c**	0.09	1.09	−1.39	–
**5d**	0.04	1.01	−1.47	–
**5e**	0.06	0.95	−1.38	−1.49

## Conclusion

Glaser–Hay homocoupling of 2-ethynyl-7-(arylethynyl)-1,8-bis(dimethylamino)naphthalenes yielded a series of previously unknown butadiynes **5** containing two fragments of arylethynyl substituted DMAN. The oligomers synthesized in this way are cross-conjugated systems, in which two independent conjugation pathways are realized: π-conjugation of DMAN fragments through a butadiyne linker and aryl–C≡C–DMAN conjugation paths. A comprehensive study by X-ray diffraction, NMR spectroscopy and cyclic voltammetry revealed that the “butadiyne pathway” is realized in cases where the aryl substituent is *p*-methoxyphenyl, phenyl or *p*-(trifluoromethyl)phenyl. Oligomers **5** bearing a π-acceptor *para*-nitro or *para*-cyano substituent on the aryl termini are A–π–D–π–D–π–A systems, which are characterized by the donor–acceptor conjugation pathway between the π-excessive DMAN residue and the π-deficient aryl ring. The effectiveness of this conjugation pathway was also confirmed by UV–vis spectroscopy data. Butadiynes **5** having electron-withdrawing CN and NO_2_ substituents in the aryl moieties demonstrated the longest wavelength absorption maxima in the series. The optical band gaps (*E*_g_^opt^), estimated from the onset point of the absorption spectra of **5**, ranged within 2.39 eV (for phenyl and *p*-methoxyphenyl derivatives) to 2.09 eV (for *p*-nitrophenyl derivative). Thus, the HOMO–LUMO gap is significantly reduced by the introduction of the electron-withdrawing substituent, while the introduction of a donor substituent, e.g., a OMe group, does not change this value. On the other hand, compounds **5a**–**c** (Ar = Ph, 4-MeOC_6_H_4_, 4-CF_3_C_6_H_4_) were red-shifted by 18–39 nm relatively to the corresponding monomers. Their absorption maxima were rather close to those of butadiyne **1** end-capped by two DMAN residues.

In doubly protonated tetrafluoroborate salts of the oligomers, conjugation between the aryl and naphthalene fragments becomes preferable, albeit diminished compared to bases **5**. In the case of the 4-methoxyphenyl derivative, protonation results in the transformation of the D–π–D–π–D–π–D system into the D–π–A–π–A–π–D system.

Butadiynes **5** with terminal phenyl and *p*-methoxyphenyl groups demonstrated the lowest first oxidation potentials and the highest second oxidation rates, whereas *p*-nitrophenyl analogs showed the lowest oxidation rates and two quasi-reversible reduction waves with lower *E*_1/2_^ox^ compared to other oligomers **5**.

Upon prolonged exposure to a CHCl_3_/EtOH mixture, *p*-CF_3_C_6_H_4_-terminated butadiyne **5** gradually underwent demethylation/acid-catalyzed heterocyclization involving one of the dimethylamino groups and the adjacent C≡C bond of the butadiyne linker, forming the corresponding benzo[*g*]indole derivative.

## Supporting Information

File 1 Experimental section.
