# Supplementary material for: Synthesis, structure, and properties of switchable cross-conjugated 1,4-diaryl-1,3-butadiynes based on 1,8-bis(dimethylamino)naphthalene
Source: Beilstein J Org Chem. 2023 May 15;19:674–86. doi: 10.3762/bjoc.19.49 (PMC10204205; doi:10.3762/bjoc.19.49)
Supplement: File 1 — Experimental section. [file Beilstein_J_Org_Chem-19-674-s001.pdf]

BEILSTEIN JOURNAL OF ORGANIC CHEMISTRY

Supporting Information

for

Synthesis, structure, and properties of switchable cross-conjugated 1,4-diaryl-1,3-butadiynes based on 1,8-bis(dimethylamino)naphthalene

Semyon V. Tsybulin, Ekaterina A. Filatova, Alexander F. Pozharskii, Valery A. Ozeryanskii and Anna V. Gulevskaya

Beilstein J. Org. Chem. **2023,** *19,* 674–686. doi:10.3762/bjoc.19.49

Experimental section

License and Terms: This is a supporting information file under the terms of the Creative Commons Attribution License (https://creativecommons.org/licenses/by/4.0). Please note that the reuse, redistribution and reproduction in particular requires that the author(s) and source are credited and that individual graphics may be subject to special legal provisions.
The license is subject to the *Beilstein Journal of Organic Chemistry* terms and conditions: (https://www.beilstein-journals.org/bjoc/terms)

Table of contents

General information S2

Experimental procedures and characterization data S2

Copies of ^{1}H and ^{13}C NMR spectra (Figures S1–S59) S11

X-ray data (Figures S60−S69) S41

References S45

General information

^{1}H and ^{13}C NMR spectra were recorded on 250 and 600 MHz spectrometers. Chemical shifts were reported in ppm relative to Me_4Si. The UV/Vis spectra of oligomers were recorded on a Varian Cary 50 Probe spectrophotometer in $CHCl_3$ and CH_3CN. The HR-ESI mass-spectra were obtained on a BRUKER maXis spectrometer equipped with an electrospray ionization (ESI) source. Melting points were determined on a Stuart SMP30 instrument in glass capillaries and are uncorrected. Flash column chromatography was performed on Al_2O_3. Commercial aryl- and (trimethylsilyl)acetylenes, amines, $Pd(PPh_3)_2Cl_2$, Pd_2dba_3, anhydrous DMSO were used as received. 2,7-Diiodo-1,8-bis(dimethylamino)naphthalene (**8**) was synthesized as it was described earlier.[1] Copper(I) acetylides were prepared from the corresponding alkynes according to the published procedure.[2]

Experimental procedures and characterization data

2-Iodo-*N*^{1},*N*^{1},*N*^{8},*N*^{8}-tetramethyl-7-(phenylethynyl)naphthalene-1,8-diamine (7a) and *N*^{1},*N*^{1},*N*^{8},*N*^{8}-tetramethyl-2,7-bis(phenylethynyl)-naphthalene-1,8-diamine (9a)

Method A. To a solution of 2,7-diiodo-1,8-bis(dimethylamino)naphthalene (**8**, 513 mg, 1.1 mmol) in dry pyridine (5 mL) dry copper(I) phenylacetylide (82 mg, 0.5 mmol) was added under argon. The reaction mixture was stirred at 80 °C for 20 h. The solvent was evaporated to dryness. The residue was purified by flash column chromatography on Al_2O_3 (2 × 20 cm) with *n*-hexane as the eluent. The first yellow fraction with R_f = 0.3–0.4 gave 114 mg (52%) of compound **7a**. The second yellow fraction with R_f = 0.1 gave 19 mg (18%) of compound **9a**.

Method B. A mixture of 2,7-diiodo-1,8-bis(dimethylamino)naphthalene (**8**, 513 mg, 1.1 mmol), CuI (9.5 mg, 0.05 mmol), Pd_2dba_3 (18 mg, 0.02 mmol), Ph_3P (39 mg, 0.15 mmol), dry DMSO (5 mL) and Et_3N (3 mL) were stirred for 20 min at 60 °C under argon. To the reaction mixture a solution of ethynylbenzene (52 mg, 0.5 mmol) in Et_3N (2 mL) was then added in portions over 1.5 h. The stirring was continued for 15 h at 60 °C. The reaction mixture was then evaporated without heating to remove the Et_3N. The residue was diluted with water (50 mL) and extracted with CH_2Cl_2 (5 × 20 mL). The combined organic extracts were dried over Na_2SO_4 and filtered. The solvent was evaporated to dryness. Flash column chromatography was then carried out similarly to *Method A*. Yield **7a**: 136 mg (62%); **9a**: 31 mg (30%).

Compound **7a** was obtained as a yellow solid, mp 105–106 °C (*n*-hexane). ^{1}H NMR (250 MHz, $CDCl_3$): δ = 2.97 (s, 6 H), 3.12 (s, 6 H), 7.19 (d, J = 8.6 Hz, 1 H), 7.34–7.46 (m, 4 H), 7.49 (d, J = 8.4 Hz, 1 H), 7.53–7.61 (m, 2 H), 7.83 (d, J = 8.6 Hz, 1 H) ppm. ^{13}C NMR (62.9 MHz, $CDCl_3$): δ = 43.6, 45.6, 90.0, 95.6, 103.2, 119.1, 124.0, 125.5, 126.6, 128.4, 128.6, 131.2, 131.4, 131.8, 137.3(2), 137.3(3), 151.1, 152.4 ppm. UV/Vis ($CHCl_3$): λ_{max} [log ε]: 287 [4.70], 301 [4.66], 332 sh [4.13], 390 sh [3.73] nm. HRMS (ESI): *m/z* calcd. for $C_{22}H_{22}IN_2^+$ [M+H$^+$]: 441.0823, found 441.0826.

Compound **9a** was obtained as a yellow solid, mp 156–157 °C (EtOH). UV/Vis ($CHCl_3$): λ_{max} [log ε]: 312 [4.83], 321 [4.84], 378 sh [3.96], 405 [4.05] nm. The compound was identical to that previously obtained in our laboratory.[3]

2-Iodo-7-((4-methoxyphenyl)ethynyl)-*N*[1],*N*[1],*N*[8],*N*[8]-tetramethylnaphthalene-1,8-diamine (7b) and 2,7-bis((4-methoxyphenyl)ethynyl)-*N*[1],*N*[1],*N*[8],*N*[8]-tetramethylnaphthalene-1,8-diamine (9b)

Method A. Compounds **7b** and **9b** were synthesized similarly to **7a** and **9a** using copper(I) (4-methoxyphenyl)acetylide (97 mg, 0.5 mmol). Flash column chromatography was carried out on Al_2O_3 (2 × 20 cm) with *n*-hexane/CH_2Cl_2 5:1 (v/v) as the eluent. The first yellow fraction with R_f = 0.3–0.4 gave compound **7b** (96 mg, 41%). The second yellow fraction was eluted using CH_2Cl_2. Fraction with R_f = 0.3 gave compound **9b** (3 mg, 2.5%).

Method B. Compounds **7b** and **9b** were synthesized similarly to **7a** and **9a** using 1-ethynyl-4-methoxybenzene (66 mg, 0.5 mmol). The purification was carried out similarly to *Method A*. Yield **7b**: 120 mg (51%); **9b**: 25 mg (21%).

Compound **7b** was obtained as a yellow solid, mp 111–112 °C (*n*-hexane). ^1H NMR (250 MHz, $CDCl_3$): δ = 2.97 (s, 6 H), 3.11 (s, 6 H), 3.85 (s, 3 H), 6.92 (d, J = 8.3 Hz, 2 H), 7.19 (d, J = 8.5 Hz, 1 H), 7.42 (d, J = 8.3 Hz, 1 H), 7.45–7.59 (m, 3 H), 7.83 (d, J = 8.5 Hz, 1 H) ppm. ^{13}C NMR (62.9 MHz, $CDCl_3$): δ = 43.6, 45.6, 55.5, 88.6, 95.7, 103.4, 114.2, 116.1, 119.5, 125.5, 126.6, 131.4, 132.0, 132.6, 137.0(9), 137.1(2), 151.0, 152.0, 159.8 ppm. UV/Vis ($CHCl_3$): λ_{max} [log ε]: 293–307 [4.66], 342 [4.31], 394 sh [3.65] nm. HRMS (ESI): *m/z* calcd. for $C_{23}H_{24}IN_2O^+$ [M+H$^+$]: 471.0928, found 471.0928.

Compound **9b** was obtained as a yellow solid, mp 161–163 °C ($CHCl_3$). ^1H NMR (250 MHz, $CDCl_3$): δ = 3.16 (s, 12 H), 3.84 (s, 6 H), 6.86–6.94 (m, 4 H), 7.33 (d, J = 8.3 Hz, 2 H), 7.39 (d, J = 8.3 Hz, 2 H), 7.45–7.53 (m, 4 H) ppm. ^{13}C NMR (62.9 MHz, $CDCl_3$): δ = 44.9, 55.5, 89.4, 94.5, 114.2, 116.5, 117.4, 123.6, 126.6, 131.3, 132.5, 137.3, 152.4, 159.6 ppm. UV/Vis ($CHCl_3$): λ_{max} [log ε]: 317–323 [4.89], 386 [4.07], 402 [4.06] nm. HRMS (ESI): *m/z* calcd. for $C_{32}H_{31}N_2O_2^+$ [M+H$^+$]: 475.2380, found 475.2386.

2-Iodo-*N*[1],*N*[1],*N*[8],*N*[8]-tetramethyl-7-((4-(trifluoromethyl)phenyl)ethynyl)naphthalene-1,8-diamine (7c) and *N*[1],*N*[1],*N*[8],*N*[8]-tetramethyl-2,7-bis((4-(trifluoromethyl)phenyl)ethynyl)naphthalene-1,8-diamine (9c)

Method A. Compounds **7c** and **9c** were synthesized similarly to **7a** and **9a** using copper(I) (4-(trifluoromethyl)phenyl)acetylide (116 mg, 0.5 mmol). Flash column chromatography was carried out on Al_2O_3 (2 × 20 cm) with *n*-hexane as the eluent. The first yellow fraction with R_f = 0.5 gave compound **7c** (114 mg, 45 %). The second yellow fraction with R_f = 0.1–0.2 gave compound **9c** (15 mg, 11 %).

Method B. Compounds **7c** and **9c** were synthesized similarly to **7a** and **9a** using 1-ethynyl-4-(trifluoromethyl)benzene (85 mg, 0.5 mmol). The purification was carried out similarly to *Method A*. Yield **7c**: 135 mg (53 %); **9c**: 30 mg (22 %).

Compound **7c** was obtained as a yellow solid, mp 87–88 °C (EtOH). ^1H NMR (250 MHz, $CDCl_3$): δ = 2.99 (s, 6 H), 3.12 (s, 6 H), 7.19 (d, J = 8.4 Hz, 1 H), 7.43 (d, J = 8.4 Hz, 1 H), 7.49 (d, J = 8.4 Hz, 1 H), 7.65 (s, 4 H), 7.85 (d, J = 8.4 Hz, 1 H) ppm. ^{13}C NMR (62.9 MHz, $CDCl_3$): δ = 43.7, 45.7, 92.5, 94.1, 102.7, 118.1, 124.1 (q, J = 272.1 Hz), 125.4(8), 125.5(4) (q, J = 3.7 Hz), 126.5, 127.8, 130.0 (q, J = 32.6 Hz), 131.2, 131.3, 131.5, 137.6, 137.8, 151.2, 152.9 ppm. UV/Vis ($CHCl_3$): λ_{max} [log ε]: 289 [4.86], 306 [4.84], 343 [4.76], 369 sh [4.66] nm. HRMS (ESI): *m/z* calcd. for $C_{23}H_{21}F_3IN_2^+$ [M+H$^+$]: 509.0696, found 509.0703.

Compound **9c** was obtained as a yellow solid, mp 148–149 °C (EtOH). ^{1}H NMR (250 MHz, $CDCl_3$): δ = 3.19 (s, 12 H), 7.33 (d, J = 8.3 Hz, 2 H), 7.41 (d, J = 8.3 Hz, 2 H), 7.63 (s, 8 H) ppm. ^{13}C NMR (62.9 MHz, $CDCl_3$): δ = 45.1, 93.4 (2C), 115.7, 123.3, 124.2 (q, J = 272.1 Hz), 125.4(8) (q, J = 3.8 Hz), 125.4(9), 128.1, 129.7 (q, J = 32.6 Hz), 131.2, 131.8, 138.3, 153.2 ppm. UV/Vis ($CHCl_3$): λ_{max} [log ε]: 296 [4.71], 316 [4.78], 329 [4.83], 374 sh [4.07], 415 [4.19] nm. HRMS (ESI): *m/z* calcd. for $C_{32}H_{25}F_6N_2^+$ [M+H$^+$]: 551.1917, found 551.1906.

4-((1,8-Bis(dimethylamino)-7-iodonaphthalen-2-yl)ethynyl)benzonitrile (7d) and 4,4'-((1,8-bis(dimethylamino)naphthalene-2,7-diyl)bis(ethyne-2,1-diyl))dibenzonitrile (9d)

Method A. Compounds **7d** and **9d** were synthesized similarly to **7a** and **9a** using copper(I) (4-cyanophenyl)acetylide (95 mg, 0.5 mmol). Flash column chromatography was carried out on Al_2O_3 (2 × 20 cm) with *n*-hexane/CH_2Cl_2 5:1 (v/v) as the eluent. The first yellow fraction with R_f = 0.3 gave compound **7d** (56 mg, 24%). The second yellow fraction with R_f = 0.6–0.7 was eluted using CH_2Cl_2. From it compound **9d** was isolated (10 mg, 9%).

Method B. Compounds **7d** and **9d** were synthesized similarly to **7a** and **9a** using 4-ethynylbenzonitrile (64 mg, 0.5 mmol). The purification was carried out similarly to *Method A*. Yield **7d**: 114 mg (49%); **9d**: 30 mg (26%).

Compound **7d** was obtained as a yellow solid, mp 153–155 °C (EtOH). ^{1}H NMR (250 MHz, $CDCl_3$): δ = 2.98 (s, 6 H), 3.11 (s, 6 H), 7.19 (d, J = 8.6 Hz, 1 H), 7.40 (d, J = 8.4 Hz, 1 H), 7.48 (d, J = 8.4 Hz, 1 H), 7.59–7.69 (m, 4 H), 7.85 (d, J = 8.6 Hz, 1 H) ppm. ^{13}C NMR (62.9 MHz, $CDCl_3$): δ = 43.7, 45.8, 93.8, 94.7, 102.4, 111.5, 117.6, 118.7, 125.4, 126.4, 128.9, 131.1, 131.3, 131.5, 132.3, 137.7, 138.0, 151.2, 153.1 ppm. UV/Vis ($CHCl_3$): λ_{max} [log ε]: 284 [4.63], 300 sh [4.61], 314 sh [4.58], 343 sh [4.33], 418 sh [3.80] nm. HRMS (ESI): *m/z* calcd. for $C_{23}H_{21}IN_3^+$ [M+H$^+$]: 466.0775, found 466.0770.

Compound **9d** was obtained as an orange solid, mp 211–212 °C (EtOH). ^{1}H NMR (250 MHz, $CDCl_3$): δ = 3.18 (s, 12 H), 7.32 (d, J = 8.4 Hz, 2 H), 7.40 (d, J = 8.4 Hz, 2 H), 7.56–7.68 (m, 8 H) ppm. ^{13}C NMR (62.9 MHz, $CDCl_3$): δ = 45.2, 93.3, 95.6, 111.2, 115.0, 118.8, 123.2, 125.0, 129.2, 131.4, 132.0, 132.3, 138.6, 153.4 ppm. UV/Vis ($CHCl_3$): λ_{max} [log ε]: 306 [4.66], 331 [4.65], 343 [4.74], 436 [4.21] nm. HRMS (ESI): *m/z* calcd. for $C_{32}H_{25}N_4^+$ [M+H$^+$]: 465.2074, found 465.2080.

2-Iodo-N^1,N^1,N^8,N^8-tetramethyl-7-((4-nitrophenyl)ethynyl)naphthalene-1,8-diamine (7e) and N^1,N^1,N^8,N^8-tetramethyl-2,7-bis((4-nitrophenyl)ethynyl)naphthalene-1,8-diamine (9e)

Method A. Compounds **7e** and **9e** were synthesized similarly to **7a** and **9a** using copper(I) (4-nitrophenyl)acetylide (105 mg, 0.5 mmol). Flash column chromatography was carried out on Al_2O_3 (2 × 20 cm) with *n*-hexane/CH_2Cl_2 5:1 (v/v) as the eluent. The first orange fraction with R_f = 0.3–0.4 gave compound **7e** (73 mg, 30%). The second red fraction with R_f = 0.6–0.7 was eluted using CH_2Cl_2. From it compound **9e** was isolated (8 mg, 5%).

Method B. Compounds **7e** and **9e** were synthesized similarly to **7a** and **9a** using 1-ethynyl-4-nitrobenzene (74 mg, 0.5 mmol). The purification was carried out similarly to *Method A*. Yield **7e**: 102 mg (42%); **9e**: 13 mg (10%).

Compound **7e** was obtained as a red solid, mp 121–122 °C (EtOH). ^{1}H NMR (250 MHz, $CDCl_3$): δ = 2.99 (s, 6 H), 3.12 (s, 6 H), 7.19 (d, J = 8.5 Hz, 1 H), 7.41 (d, J = 8.5 Hz, 1 H), 7.48 (d, J = 8.5 Hz, 1 H), 7.68 (d, J = 7.9 Hz, 2 H), 7.86 (d, J = 8.5 Hz, 1 H), 8.25 (d, J = 7.9 Hz, 2 H) ppm. ^{13}C NMR

(62.9 MHz, $CDCl_3$): δ = 43.8, 45.8, 93.7, 95.7, 102.2, 117.4, 123.9, 125.4, 126.4, 130.9, 131.1, 131.2, 131.7, 137.8, 138.1, 147.0, 151.3, 153.3 ppm. UV/Vis ($CHCl_3$): λ_{max} [log ε]: 273 sh [4.69], 320 [4.54], 357 sh [4.44] nm. HRMS (ESI): *m/z* calcd. for $C_{22}H_{21}IN_3O_2^+$ [M+H$^+$]: 486.0673, found 486.0678.

Compound **9e** was obtained as red-brown crystals with a greenish metallic luster, mp 210–211 °C (DCE). ^{1}H NMR (250 MHz, $CDCl_3$): δ = 3.21 (s, 12 H), 7.33 (d, J = 8.3 Hz, 2 H), 7.42 (d, J = 8.3 Hz, 2 H), 7.65 (d, J = 8.8 Hz, 4 H), 8.24 (d, J = 8.8 Hz, 4 H) ppm. ^{13}C NMR (62.9 MHz, $CDCl_3$): δ = 45.3, 93.2, 96.7, 114.7, 123.1, 123.9, 124.7, 131.2, 131.5, 132.1, 138.8, 146.8, 153.6 ppm. UV/Vis ($CHCl_3$): λ_{max} [log ε]: 287 [4.46], 330 [4.66], 365 sh [4.53], 465 [4.28] nm. HRMS (ESI): *m/z* calcd. for $C_{30}H_{25}N_4O_4^+$ [M+H$^+$]: 505.1871, found 505.1867.

N^1,*N*^{1},*N*^{8},*N*^{8}-Tetramethyl-2-(phenylethynyl)-7-((trimethylsilyl)ethynyl)naphthalene-1,8-diamine (10a) and 2-ethynyl-*N*^{1},*N*^{1},*N*^{8},*N*^{8}-tetramethyl-7-(phenylethynyl)naphthalene-1,8-diamine (6a)

A mixture of iodide **7a** (88 mg, 0.2 mmol), CuI (4 mg, 0.02 mmol), $Pd(PPh_3)_2Cl_2$ (7 mg, 0.01 mmol), dry DMSO (4 mL) and Et_3N (4 mL) was stirred at 80 °C for 20 min under argon. To the reaction mixture trimethylsilylacetylene (58 mg, 0.08 mL, 0.6 mmol) was then added in portions over 1 h. The stirring was continued for 15 h at 80 °C. The reaction mixture was then evaporated without heating to remove the Et_3N. The residue was diluted with water (50 mL) and extracted with CH_2Cl_2 (5 × 20 mL). The combined organic extracts were dried over Na_2SO_4 and filtered. The solvent was evaporated to dryness. The residue was treated with *n*-hexane (30 mL) and filtered; the filtrate was evaporated to dryness. The crude product was recrystallized from EtOH. Yield: 74 mg (90%). Compound **10a** was obtained as a yellow solid, mp 132–133 °C (EtOH). ^{1}H NMR (250 MHz, $CDCl_3$): δ = 0.28 (s, 9 H), 3.11 (s, 6 H), 3.16 (s, 6 H), 7.24–7.44 (m, 7 H), 7.52–7.58 (m, 2 H) ppm. ^{13}C NMR (62.9 MHz, $CDCl_3$): δ = 0.1, 44.8, 45.0, 90.8, 94.6, 99.5, 106.4, 116.4, 116.6, 123.2, 123.3, 124.3, 126.0, 128.1, 128.5, 131.1, 131.6, 131.9, 137.8, 152.8, 153.5 ppm. UV/Vis ($CHCl_3$): λ_{max} [log ε]: 281 [4.57], 294 [4.74], 298 sh [4.73], 312 [4.80], 331 sh [4.06], 370 [3.93], 400 [3.98] nm. HRMS (ESI): *m/z* calcd. for $C_{27}H_{31}N_2Si^+$ [M+H$^+$]: 411.2251, found 411.2254.

The column chromatography of crude product **10a** on Al_2O_3 (2 × 20 cm) with *n*-hexane/CH_2Cl_2 5:1 (v/v) as the eluent led to its full desilylation and formation of terminal alkyne **6a**. Compound **6a** was obtained as a yellow solid, mp 85–86 °C (EtOH). ^{1}H NMR (250 MHz, $CDCl_3$): δ = 3.13 (s, 6 H), 3.17 (s, 6 H), 3.39 (s, 1 H), 7.28–7.44 (m, 7 H), 7.52–7.59 (m, 2 H) ppm. ^{13}C NMR (62.9 MHz, $CDCl_3$): δ = 44.9, 45.0, 82.6, 84.8, 90.7, 94.7, 115.6, 116.7, 123.3, 123.4, 124.3, 126.0, 128.1, 128.5, 131.1, 131.7, 132.0, 137.9, 152.8, 153.7 ppm. UV/Vis ($CHCl_3$): λ_{max} [log ε]: 291 [4.68], 296 [4.67], 308 [4.77], 393 [3.98] nm. HRMS (ESI): *m/z* calcd. for $C_{24}H_{23}N_2^+$ [M+H$^+$]: 339.1856, found 339.1860.

2-((4-Methoxyphenyl)ethynyl)-*N*^{1},*N*^{1},*N*^{8},*N*^{8}-tetramethyl-7-((trimethylsilyl)ethynyl)naphthalene-1,8-diamine (10b) and 2-ethynyl-7-((4-methoxyphenyl)ethynyl)-*N*^{1},*N*^{1},*N*^{8},*N*^{8}-tetramethyl-naphthalene-1,8-diamine (6b)

Compound **10b** was obtained similarly to **10a** starting from iodide **7b** (94 mg, 0.2 mmol). Yield: 70 mg (80%). Compound **10b** was obtained as a yellow solid, mp 145–146 °C (EtOH). ^{1}H NMR (250 MHz, $CDCl_3$): δ = 0.28 (s, 9 H), 3.11 (s, 6 H), 3.14 (s, 6 H), 3.84 (s, 3 H), 7.86–7.94 (m, 2 H), 7.24–7.34 (m, 3 H), 7.38 (d, J = 8.2 Hz, 1 H), 7.45–7.52 (m, 2 H) ppm. ^{13}C NMR (62.9 MHz, $CDCl_3$): δ = 0.1, 44.8, 44.9, 55.5, 89.4, 94.6, 99.5, 106.5, 114.2, 116.5, 116.6, 117.2, 123.3, 123.4, 126.3, 131.6,

131.7, 132.5, 137.6, 152.5, 153.5, 159.6 ppm. UV/Vis ($CHCl_3$): λ_{max} [log ε]: 302 sh [4.73], 315 [4.80], 340 sh [4.15], 375 [4.00], 398 [4.00] nm. HRMS (ESI): *m/z* calcd. for $C_{28}H_{33}N_2OSi^+$ [M+H$^+$]: 441.2357, found 441.2364.

The column chromatography of crude product **10b** on Al_2O_3 (2 × 20 cm) with *n*-hexane/CH_2Cl_2 5:1 (v/v) as the eluent led to its full desilylation and formation of terminal alkyne **6b**. Compound **6b** was obtained as a yellow solid, mp 121–122 °C (EtOH). ^{1}H NMR (250 MHz, $CDCl_3$): δ = 3.12 (s, 6 H), 3.15 (s, 6 H), 3.38 (s, 1 H), 3.84 (s, 3 H), 6.90 (d, *J* = 8.2 Hz, 2 H), 7.27–7.43 (m, 4 H), 7.49 (d, *J* = 8.2 Hz, 2 H) ppm. ^{13}C NMR (62.9 MHz, $CDCl_3$): δ = 44.9, 45.0, 55.5, 82.6, 84.8, 89.3, 94.7, 114.2, 115.7, 116.4, 117.3, 123.4, 123.5, 126.2, 131.7, 131.8, 132.5, 137.7, 152.4, 153.6, 159.6 ppm. UV/Vis ($CHCl_3$): λ_{max} [log ε]: 297 sh [4.63], 312 [4.72], 338 sh [4.05], 405 sh [3.92] nm. HRMS (ESI): *m/z* calcd. for $C_{25}H_{25}N_2O^+$ [M+H$^+$]: 369.1961, found 369.1967.

N^1,*N*^{1},*N*^{8},*N*^{8}-Tetramethyl-2-((4-(trifluoromethyl)phenyl)ethynyl)-7-((trimethylsilyl)ethynyl)-naphthalene-1,8-diamine (10c) and 2-ethynyl-*N*^{1},*N*^{1},*N*^{8},*N*^{8}-tetramethyl-7-((4-(trifluoromethyl)-phenyl)ethynyl)naphthalene-1,8-diamine (6c)

Compound **10c** was obtained similarly to **10a** starting from iodide **7c** (102 mg, 0.2 mmol). Yield: 81 mg (85%). Compound **10c** was obtained as a yellow solid, mp 136–137 °C (EtOH). ^{1}H NMR (250 MHz, $CDCl_3$): δ = 0.28 (s, 9 H), 3.11 (s, 6 H), 3.16 (s, 6 H), 7.23–7.41 (m, 4 H), 7.62 (s, 4 H) ppm. ^{13}C NMR (62.9 MHz, $CDCl_3$): δ = 0.1, 44.9, 45.1, 93.2, 93.5, 99.8, 106.4, 115.6, 116.5, 123.1, 123.3, 124.2 (q, *J* = 272.1 Hz), 125.5 (q, *J* = 3.8 Hz), 125.7, 128.2, 129.6 (q, *J* = 32.6 Hz), 131.2, 131.5, 132.3, 138.2, 153.3, 153.5 ppm. UV/Vis ($CHCl_3$): λ_{max} [log ε]: 280 sh [4.55], 294 [4.69], 304 sh [4.64], 316 [4.71], 408 [4.02] nm. HRMS (ESI): *m/z* calcd. for $C_{28}H_{30}F_3N_2Si^+$ [M+H$^+$]: 479.2125, found 479.2127.

The column chromatography of crude product **10c** on Al_2O_3 (2 × 20 cm) with *n*-hexane/CH_2Cl_2 5:1 (v/v) as the eluent led to its full desilylation and formation of terminal alkyne **6c**. Compound **6c** was obtained as a yellow solid, mp 78–79 °C (EtOH). ^{1}H NMR (250 MHz, $CDCl_3$): δ = 3.12 (s, 6 H), 3.17 (s, 6 H), 3.39 (s, 1 H), 7.27–7.42 (m, 4 H), 7.62 (s, 4 H) ppm. ^{13}C NMR (62.9 MHz, $CDCl_3$): δ = 44.9, 45.1, 82.8, 84.7, 93.3, 93.4, 115.6, 115.7, 123.3, 123.4, 124.2 (q, *J* = 272.1 Hz), 125.5 (q, *J* = 3.9 Hz), 125.7, 128.1, 129.7 (q, *J* = 32.6 Hz), 131.2, 131.6, 132.5, 138.2, 153.3, 153.7 ppm. UV/Vis ($CHCl_3$): λ_{max} [log ε]: 276 sh [4.58], 291 [4.70], 299 sh [4.68], 313 [4.75], 400 [4.08] nm. HRMS (ESI): *m/z* calcd. for $C_{25}H_{22}F_3N_2^+$ [M+H$^+$]: 407.1730, found 407.1730.

4-((1,8-Bis(dimethylamino)-7-((trimethylsilyl)ethynyl)naphthalen-2-yl)ethynyl)benzonitrile (10d) and 4-((1,8-bis(dimethylamino)-7-ethynylnaphthalen-2-yl)ethynyl)benzonitrile (6d)

Compound **10d** was obtained similarly to **10a** starting from iodide **7d** (93 mg, 0.2 mmol). Yield: 72 mg (83%). Compound **10d** was obtained as a yellow solid, mp 170–172 °C (EtOH). ^{1}H NMR (250 MHz, $CDCl_3$): δ = 0.27 (s, 9 H), 3.11 (s, 6 H), 3.15 (s, 6 H), 7.22–7.38 (m, 4 H), 7.56–7.67 (m, 4 H) ppm. ^{13}C NMR (62.9 MHz, $CDCl_3$): δ = 0.0, 44.9, 45.1, 93.0, 95.8, 99.8, 106.3, 111.0, 114.9, 116.3, 118.8, 123.0, 123.2, 125.3, 129.3, 131.4 (2C), 132.2, 132.5, 138.3, 153.5 (2C) ppm. UV/Vis ($CHCl_3$): λ_{max} [log ε]: 283 sh [4.56], 294 [4.62], 307 [4.62], 325 [4.63], 413 [4.06], 442 sh [3.98] nm. HRMS (ESI): *m/z* calcd. for $C_{28}H_{29}N_3Si^+$ [M+H$^+$]: 436.2204, found 436.2206.

The column chromatography of crude product **10d** on Al_2O_3 (2 × 20 cm) with *n*-hexane/CH_2Cl_2 5:1 (v/v) as the eluent led to its full desilylation and formation of terminal alkyne **6d**. Compound **6d**

was obtained as a yellow solid, mp 138–139 °C (EtOH). ^{1}H NMR (250 MHz, $CDCl_3$): δ = 3.12 (s, 6 H), 3.16 (s, 6 H), 3.39 (s, 1 H), 7.26–7.39 (m, 4 H), 7.56–7.67 (m, 4 H) ppm. ^{13}C NMR (62.9 MHz, $CDCl_3$): δ = 44.9, 45.2, 82.9, 84.6, 93.1, 95.7, 111.0, 115.0, 115.4, 118.8, 123.1, 123.2, 125.3, 129.2, 131.4, 131.5, 132.2, 132.7, 138.4, 153.4, 153.7 ppm. UV/Vis ($CHCl_3$): λ_{max} [log ε]: 288 [4.60], 307 [4.62], 321 [4.64], 407 [4.08], 441 sh [4.08] nm. HRMS (ESI): *m/z* calcd. for $C_{25}H_{22}N_3^+$ [M+H$^+$]: 364.1808, found 364.1814.

***N*^{1},*N*^{1},*N*^{8},*N*^{8}-Tetramethyl-2-((4-nitrophenyl)ethynyl)-7-((trimethylsilyl)ethynyl)naphthalene-1,8-diamine (10e) and 2-ethynyl-*N*^{1},*N*^{1},*N*^{8},*N*^{8}-tetramethyl-7-((4-nitrophenyl)ethynyl)naphthalene-1,8-diamine (6e)**

Compound **10e** was obtained similarly to **10a** starting from iodide **7e** (97 mg, 0.2 mmol). Yield: 76 mg (83%). Compound **10e** was obtained as a red solid, mp 142–144 °C (EtOH). ^{1}H NMR (250 MHz, $CDCl_3$): δ = 0.27 (s, 9 H), 3.12 (s, 6 H), 3.17 (s, 6 H), 7.23–7.39 (m, 4 H), 7.60–7.68 (m, 2 H), 8.19–8.27 (m, 2 H) ppm. ^{13}C NMR (62.9 MHz, $CDCl_3$): δ = 0.0, 44.9, 45.2, 93.0, 97.0, 99.9, 106.2, 114.6, 116.3, 122.9, 123.2, 123.9, 125.2, 131.3(5), 131.3(9), 131.5, 132.7, 138.4, 146.7, 153.5, 153.6 ppm. UV/Vis ($CHCl_3$): λ_{max} [log ε]: 281 sh [4.54], 290 [4.60], 331 [4.47], 408 [4.03], 460 sh [3.86] nm. HRMS (ESI): *m/z* calcd. for $C_{27}H_{30}N_3O_2Si^+$ [M+H$^+$]: 456.2102, found 456.2103.

The column chromatography of crude product **10e** on Al_2O_3 (2 × 20 cm) with *n*-hexane/CH_2Cl_2 5:1 (v/v) as the eluent led to its full desilylation and formation of terminal alkyne **6e**. Compound **6e** was obtained as a red solid, mp 118–119 °C (EtOH). ^{1}H NMR (250 MHz, $CDCl_3$): δ = 3.12 (s, 6 H), 3.17 (s, 6 H), 3.39 (s, 1 H), 7.26–7.40 (m, 4 H), 7.60–7.68 (m, 2 H), 8.19–8.26 (m, 2 H) ppm. ^{13}C NMR (62.9 MHz, $CDCl_3$): δ = 45.0, 45.2, 82.9, 84.6, 93.0, 96.9, 114.8, 115.4, 123.1, 123.2, 123.9, 125.2, 131.3, 131.4(9), 131.5(2), 132.8, 138.5, 146.8, 153.6, 153.7 ppm. UV/Vis ($CHCl_3$): λ_{max} [log ε]: 275 sh [4.52], 284 [4.56], 328 [4.49], 404 [4.05], 456 sh [3.92] nm. HRMS (ESI): *m/z* calcd. for $C_{24}H_{22}N_3O_2^+$ [M+H$^+$]: 384.1707, found 384.1713.

7,7'-(Buta-1,3-diyne-1,4-diyl)bis(*N*^{1},*N*^{1},*N*^{8},*N*^{8}-tetramethyl-2-(phenylethynyl)naphthalene-1,8-diamine) (5a)

A mixture of alkyne **6a** (94 mg, 0.28 mmol), CuI (29 mg, 0.15 mmol), TMEDA (45 µL, 0.3 mmol) and diisopropylamine (5 mL) was stirred at room temperature for 20 h under air. The reaction mixture was then evaporated without heating to remove diisopropylamine. The residue was purified by flash column chromatography on Al_2O_3 (2 × 20 cm) using CH_2Cl_2 as the eluent. The orange fraction with R_f = 0.3 gave compound **5a** (59 mg, 63%) as a yellow-orange solid, mp 208−209 °C (EtOAc). ^{1}H NMR (600 MHz, $CDCl_3$): δ = 3.18(0) (s, 12 H), 3.18(1) (s, 12 H), 7.27 (dd, *J* = 8.3, 0.5 Hz, 2 H), 7.30 (dd, *J* = 8.2, 0.5 Hz, 2 H), 7.32–7.39 (m, 8 H), 7.41 (d, *J* = 8.3 Hz, 2 H), 7.53–7.55 (m, 2 H), 7.55–7.56 (m, 2 H) ppm. ^{13}C NMR (150 MHz, $CDCl_3$): δ = 45.0, 45.2, 80.2, 84.1, 90.8, 94.8, 114.7, 116.4, 123.0, 123.1, 124.3, 125.2, 128.1, 128.6, 131.1, 131.8, 132.1, 138.1, 152.7, 154.6 ppm. UV/Vis ($CHCl_3$): λ_{max} [log ε]: 308 sh [4.93], 323 [5.04], 337 sh [4.92], 432 [4.51] nm. HRMS (ESI): *m/z* calcd. for $C_{48}H_{43}N_4^+$ [M+H$^+$]: 675.3482, found 675.3482; calcd. for $C_{48}H_{44}N_4^{2+}$ [M+2H$^+$]: 338.1778, found 338.1771.

7,7'-(Buta-1,3-diyne-1,4-diyl)bis(2-((4-methoxyphenyl)ethynyl)-N^1,N^1,N^8,N^8-tetramethyl-naphthalene-1,8-diamine) (5b)

Compound **5b** was obtained similarly to **5a** starting from alkyne **6b** (103 mg, 0.28 mmol). Yield: 66 mg (64%). Compound **5b** was obtained as a yellow-orange solid, mp 204–205 °C (EtOAc). ^{1}H NMR (600 MHz, $CDCl_3$): δ = 3.16 (s, 12 H), 3.17 (s, 12 H), 3.84 (s, 6 H), 6.90 (m, 4 H), 7.27 (d, J = 8.3 Hz, 2 H), 7.29 (d, J = 8.3 Hz, 2 H), 7.35 (d, J = 8.3 Hz, 2 H), 7.39 (d, J = 8.3 Hz, 2 H), 7.47–7.49 (m, 4 H) ppm. ^{13}C NMR (150 MHz, $CDCl_3$): δ = 45.0, 45.2, 55.5, 80.2, 84.1, 89.4, 94.7, 114.3, 114.8, 116.5, 116.9, 123.1, 123.2, 125.5, 131.6, 132.1, 132.5, 137.9, 152.4, 154.6, 159.7 ppm. UV/Vis ($CHCl_3$): λ_{max} [log ε]: 327 [5.10], 338 sh [5.02], 423 [4.54] nm. HRMS (ESI): *m/z* calcd. for $C_{50}H_{47}N_4O_2^+$ [M+H$^+$]: 735.3694, found 735.3694; calcd. for $C_{50}H_{48}N_4O_2^{2+}$ [M+2H$^+$]: 368.1883, found 368.1883.

7,7'-(Buta-1,3-diyne-1,4-diyl)bis(N^1,N^1,N^8,N^8-tetramethyl-2-((4-(trifluoromethyl)phen-yl)ethynyl)naphthalene-1,8-diamine) (5c)

Compound **5c** was obtained similarly to **5a** starting from alkyne **6c** (101 mg, 0.25 mmol). Yield: 65 mg (64%). Compound **5c** was obtained as a yellow solid, mp 224–225 °C (EtOAc). ^{1}H NMR (600 MHz, $CDCl_3$): δ = 3.18(0) (s, 12 H), 3.18(3) (s, 12 H), 7.27 (dd, J = 8.3, 0.5 Hz, 2 H), 7.30 (dd, J = 8.3, 0.5 Hz, 2 H), 7.37–7.40 (m, 4 H), 7.62 (s, 8 H) ppm. ^{13}C NMR (150 MHz, $CDCl_3$): δ = 45.1, 45.2, 80.3, 84.1, 93.4, 93.5, 114.6, 115.3, 123.0, 123.1, 124.2 (q, J = 272.1 Hz), 124.9, 125.5 (q, J = 3.7 Hz), 128.1, 129.7 (q, J = 32.6 Hz), 131.2, 132.0, 132.2, 138.5, 153.2, 154.6 ppm. UV/Vis ($CHCl_3$): λ_{max} [log ε]: 315 sh [4.99], 327 [5.06], 340 sh [4.98], 437 [4.64] nm. HRMS (ESI): *m/z* calcd. for $C_{50}H_{41}F_6N_4^+$ [M+H$^+$]: 811.3230, found 811.3230; calcd. for $C_{50}H_{42}F_6N_4^{2+}$ [M+2H$^+$]: 406.1651, found 406.1659.

4,4'-((Buta-1,3-diyne-1,4-diylbis(1,8-bis(dimethylamino)naphthalene-7,2-diyl))bis(ethyne-2,1-diyl))-dibenzonitrile (5d)

Compound **5d** was obtained similarly to **5a** starting from alkyne **6d** (109 mg, 0.3 mmol). Yield: 72 mg (66%). Compound **5d** was obtained as an orange solid, mp 230–232 °C (decomp.) (DCE). ^{1}H NMR (600 MHz, $CDCl_3$): δ = 3.17 (s, 12 H), 3.18 (s, 12 H), 7.26 (dd, J = 8.3, 0.5 Hz, 2 H), 7.29 (dd, J = 8.3, 0.5 Hz, 2 H), 7.37 (d, J = 7.0 Hz, 2 H), 7.39 (d, J = 7.0 Hz, 2 H), 7.58–7.60 (m, 4 H), 7.63–7.66 (m, 4 H) ppm. ^{13}C NMR (150 MHz, $CDCl_3$): δ = 45.1(8), 45.2(4), 80.3, 84.0, 93.2, 95.8, 111.2, 114.4, 114.7, 118.8, 122.9, 123.0, 124.6, 129.2, 131.4, 131.9, 132.3, 132.5, 138.6, 153.4, 154.6 ppm. UV/Vis ($CHCl_3$): λ_{max} [log ε]: 318 [5.04], 326 [5.04], 338 [5.06], 385 sh [4.51], 449 [4.54] nm. HRMS (ESI): *m/z* calcd. for $C_{50}H_{41}N_6^+$ [M+H$^+$]: 725.3387, found 725.3393; calcd. for $C_{50}H_{42}N_6^{2+}$ [M+2H$^+$]: 363.1730, found 363.1726.

7,7'-(Buta-1,3-diyne-1,4-diyl)bis(N^1,N^1,N^8,N^8-tetramethyl-2-((4-nitrophenyl)ethyn-yl)naphthalene-1,8-diamine) (5e)

Compound **5e** was obtained similarly to **5a** starting from alkyne **6e** (115 mg, 0.3 mmol). Yield: 73 mg (64%). Compound **5e** was obtained as a dark red solid, mp 234–235 °C (decomp.) (DCE). ^{1}H NMR (600 MHz, $CDCl_3$): δ = 3.18 (s, 12 H), 3.20 (s, 12 H), 7.27 (d, J = 8.2 Hz, 2 H), 7.29 (d, J = 8.2 Hz, 2 H), 7.38–7.40 (m, 4 H), 7.63–7.66 (m, 4 H), 8.22–8.25 (m, 4 H) ppm. ^{13}C NMR (150 MHz, $CDCl_3$): δ = 45.2, 45.3, 80.4, 84.1, 93.2, 96.9, 114.4, 122.9, 123.0, 123.9, 124.5, 131.3, 131.5, 132.0,

132.6, 138.7, 146.9, 153.5, 154.6 ppm. UV/Vis ($CHCl_3$): λ_{max} [log ε]: 310 [4.83], 332 [4.86], 453 [4.58] nm. HRMS (ESI): *m/z* calcd. for $C_{48}H_{41}N_6O_4^+$ [M+H$^+$]: 765.3184, found 765.3180.

2,2'-(Buta-1,3-diyne-1,4-diyl)bis(7-(arylethynyl)-8-(dimethylamino)-*N,N*-dimethylnaphthalen-1-aminium) bis(tetrafluoroborates) (11) (general procedure)

To a solution of compound **5** (0.025 mmol) in CH_2Cl_2 (5–6 mL) 40% aqueous HBF_4 (2.5-fold excess) was added. The reaction mixture was thoroughly stirred for several minutes and diluted with a double volume of Et_2O. The resulting precipitate was filtered off, washed with Et_2O and dried in a vacuum giving rise to the desired salt **11**.

2,2'-(Buta-1,3-diyne-1,4-diyl)bis(8-(dimethylamino)-N,N-dimethyl-7-(phenylethynyl)naphthalen-1-aminium) bis(tetrafluoroborate) (**11a**) was obtained in 79% as a gray solid (decomp. > 225 °C). ^{1}H NMR (600 MHz, DMSO-d$_6$): 3.45 (d, *J* = 2.1 Hz, 12 H), 3.54 (d, *J* = 2.9 Hz, 12 H), 7.54–7.58 (m, 6 H), 7.75–7.79 (m, 4 H), 7.96 (d, *J* = 8.5 Hz, 2 H), 7.98 (d, *J* = 8.5 Hz, 2 H), 8.21–8.27 (m, 4 H), 19.76 (s, 2 H) ppm. ^{13}C NMR (62.9 MHz, DMSO-d$_6$): 42.8, 43.0, 80.8, 82.9, 84.9, 100.8, 116.6, 118.5, 120.7, 120.9, 129.0, 129.6, 129.8, 130.2, 131.4, 132.6, 133.0, 134.9, 143.0, 146.8 ppm. UV/Vis (CH_3CN): λ_{max} [log ε]: 286 sh [4.67], 306 [4.77], 316 [4.77], 328 sh [4.72], 345 [4.49], 371 [4.39], 382 sh [4.28] nm.

2,2'-(Buta-1,3-diyne-1,4-diyl)bis(8-(dimethylamino)-7-((4-methoxyphenyl)ethynyl)-N,N-dimethylnaphthalen-1-aminium) bis(tetrafluoroborate) (**11b**) was obtained in 78% as a pale gray solid (decomp. > 222 °C). ^{1}H NMR (600 MHz, DMSO-d$_6$): 3.44 (d, *J* = 2.1 Hz, 12 H), 3.53 (d, *J* = 3.0 Hz, 12 H), 3.85 (s, 6 H), 7.08–7.12 (m, 4 H), 7.69–7.73 (m, 4 H), 7.92 (d, *J* = 8.5 Hz, 2 H), 7.96 (d, *J* = 8.5 Hz, 2 H), 8.21 (d, *J* = 4.2 Hz, 2 H), 8.22 (d, *J* = 4.2 Hz, 2 H), 19.73 (s, 2 H) ppm. ^{13}C NMR (62.9 MHz, DMSO-d$_6$): 42.7, 43.0, 55.5, 80.9, 82.9, 84.0, 101.4, 112.8, 114.8, 116.7, 118.9, 120.8, 129.5, 129.7, 132.5, 133.0, 133.2, 134.7, 142.4, 146.8, 160.7 ppm. UV/Vis (CH_3CN): λ_{max} [log ε]: 273 [4.56], 283 [4.56], 309 sh [4.64], 326 [4.75], 341 [4.78], 370 [4.48], 387 sh [4.31] nm.

2,2'-(Buta-1,3-diyne-1,4-diyl)bis(8-(dimethylamino)-N,N-dimethyl-7-((4-(trifluoromethyl)-phenyl)ethynyl)naphthalen-1-aminium) bis(tetrafluoroborate) (**11c**) was obtained in 86% as a pale yellow solid (decomp. > 228 °C). ^{1}H NMR (600 MHz, DMSO-d$_6$): 3.47 (d, *J* = 2.2 Hz, 12 H), 3.53 (d, *J* = 2.8 Hz, 12 H), 7.92 (d, *J* = 8.2 Hz, 4 H), 7.96–8.03 (m, 8 H), 8.25 (d, *J* = 8.7 Hz, 2 H), 8.27 (d, *J* = 8.7 Hz, 2 H), 19.73 (s, 2 H) ppm. ^{13}C NMR (62.9 MHz, DMSO-d$_6$): 42.9, 43.1, 80.8, 83.0, 87.1, 98.9, 116.7, 118.0, 120.8, 123.8 (q, *J* = 272.5 Hz), 125.2, 125.9 (q, *J* = 3.1 Hz), 129.6, 129.8, 129.9 (q, *J* = 32.1 Hz), 132.3, 132.9, 133.1, 135.1, 143.7, 146.8 ppm. UV/Vis (CH_3CN): λ_{max} [log ε]: 290 sh [4.74], 312 [4.80], 320 [4.80], 331 sh [4.73], 347 sh [4.51], 371 [4.38], 382 sh [4.30] nm.

2,2'-(Buta-1,3-diyne-1,4-diyl)bis(7-((4-cyanophenyl)ethynyl)-8-(dimethylamino)-N,N-dimethyl-naphthalen-1-aminium) bis(tetrafluoroborate) (**11d**) was obtained in 76% as a beige solid (decomp. > 227 °C). ^{1}H NMR (600 MHz, DMSO-d$_6$): 3.46 (d, *J* = 2.2 Hz, 12 H), 3.52 (d, *J* = 2.8 Hz, 12 H), 7.95–7.98 (m, 4 H), 7.99–8.01 (m, 4 H), 8.02–8.06 (m, 4 H), 8.25 (d, *J* = 8.8 Hz, 2 H), 8.27 (d, *J* = 8.8 Hz, 2 H), 19.76 (s, 2 H) ppm. ^{13}C NMR (62.9 MHz, DMSO-d$_6$): 43.0, 43.1, 80.8, 83.0, 88.3, 98.8, 112.3, 116.7, 117.9, 118.2, 120.7, 125.7, 129.6, 129.8, 132.3, 132.8, 132.9(5), 133.0(4), 135.2, 143.8, 146.8 ppm. UV/Vis (CH_3CN): λ_{max} [log ε]: 285 sh [4.60], 305 sh [4.73], 321 [4.76], 339 [4.71], 371 [4.33], 383 sh [4.24] nm.

2,2'-(Buta-1,3-diyne-1,4-diyl)bis(8-(dimethylamino)-N,N-dimethyl-7-((4-nitrophenyl)ethynyl)-naphthalen-1-aminium) bis(tetrafluoroborate) (**11e**) was obtained in 80% as a gray solid (decomp. > 230 °C). ^{1}H NMR (600 MHz, DMSO-d_6): 3.47 (d, *J* = 2.3 Hz, 12 H), 3.53 (d, *J* = 2.8 Hz, 12 H), 8.01 (d, *J* = 8.5 Hz, 2 H), 8.02 (d, *J* = 8.5 Hz, 2 H), 8.04–8.07 (m, 4 H), 8.26 (d, *J* = 8.8 Hz, 2 H), 8.28 (d, *J* = 8.8 Hz, 2 H), 8.36–8.40 (m, 4 H), 19.77 (s, 2 H) ppm. ^{13}C NMR (62.9 MHz, DMSO-d_6): 43.0, 43.1, 80.8, 83.1, 89.0, 98.5, 116.6, 117.8, 120.8, 124.0, 127.6, 129.7, 129.8, 132.8, 133.0, 133.1, 135.2, 144.0, 146.8, 147.8 ppm. UV/Vis (CH_3CN): λ_{max} [log ε]: 284 sh [4.36], 307 sh [4.46], 325 [4.54], 347 [4.56], 369 sh [4.37], 385 sh [4.19] nm.

Copies of NMR spectra

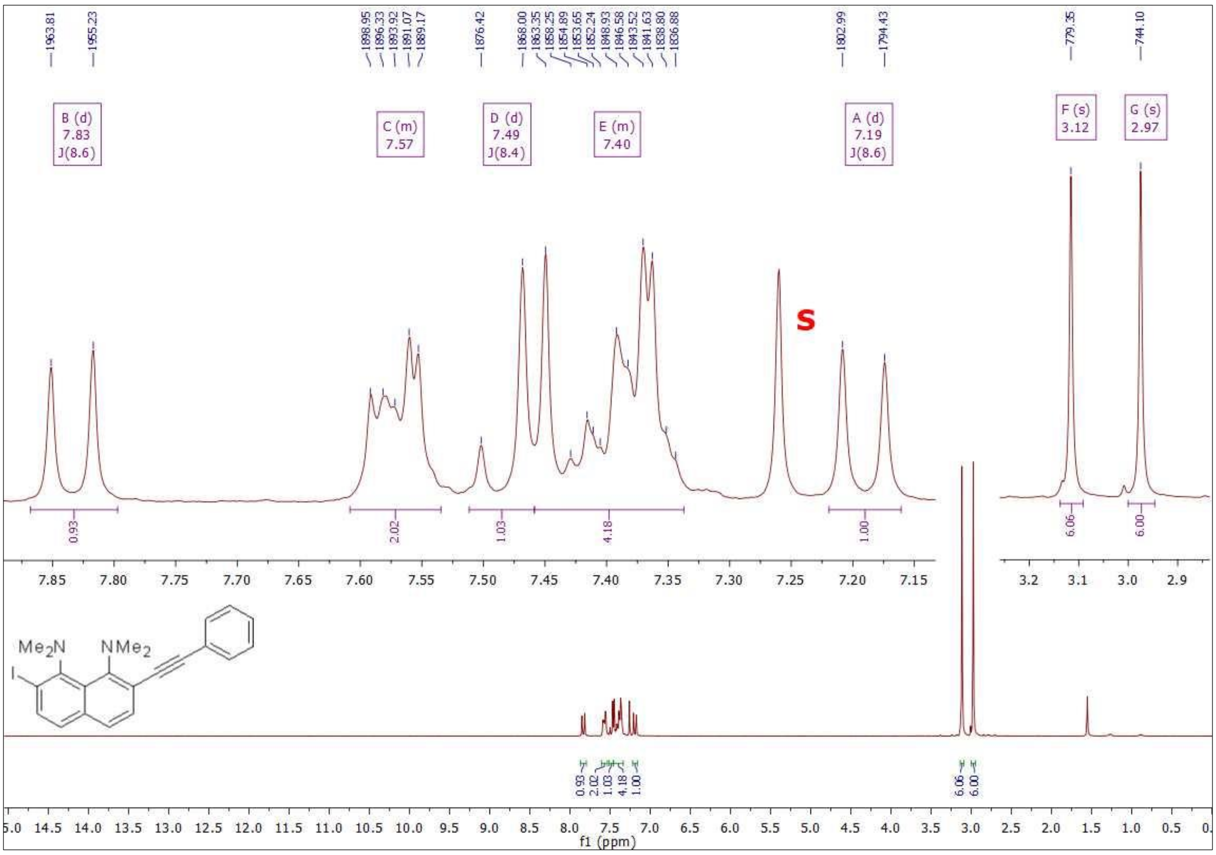

Figure S1. ^{1}H NMR spectrum of compound **7a** (250 MHz, $CDCl_3$).

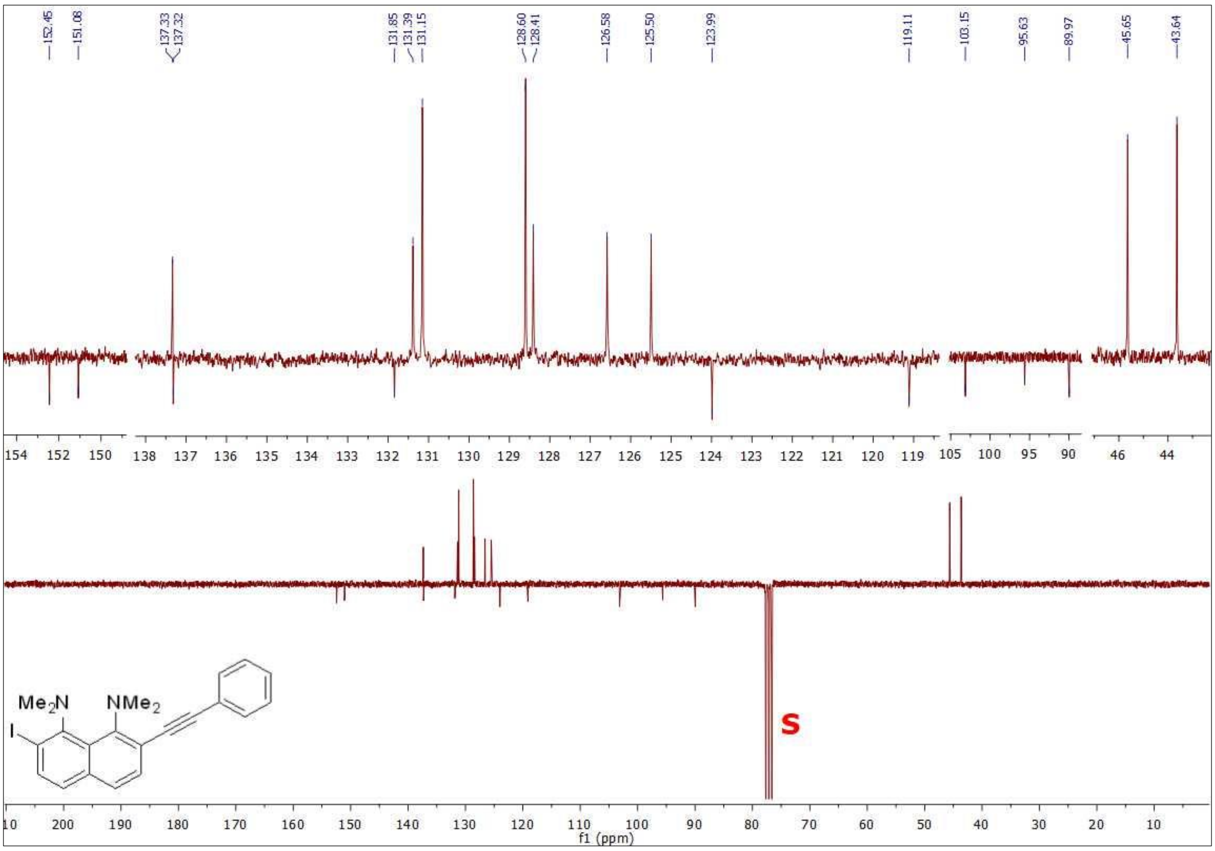

Figure S2. $^{13}C\{^{1}H\}$ APT-NMR spectrum of compound **7a** (62.9 MHz, $CDCl_3$).

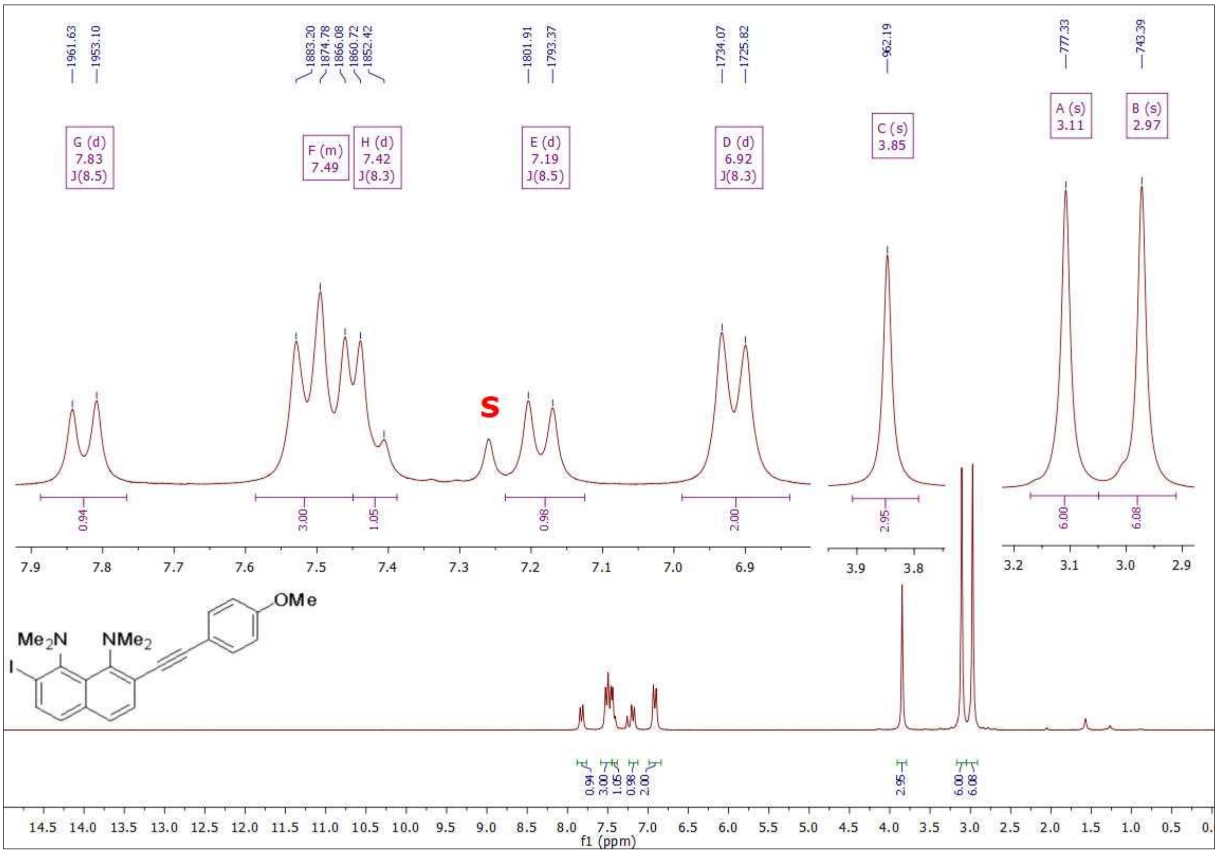

Figure S3. ^{1}H NMR spectrum of compound **7b** (250 MHz, $CDCl_3$).

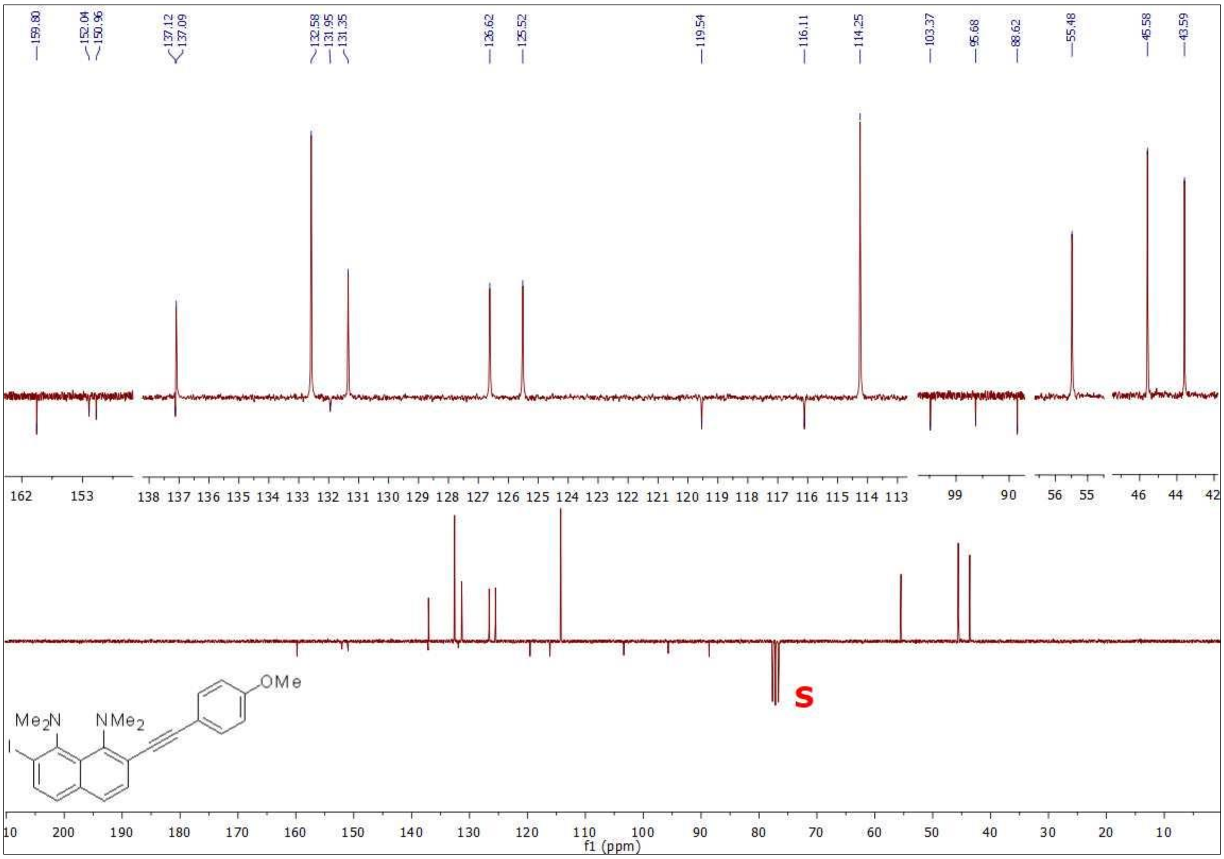

Figure S4. $^{13}C\{^{1}H\}$ APT-NMR spectrum of compound **7b** (62.9 MHz, $CDCl_3$).

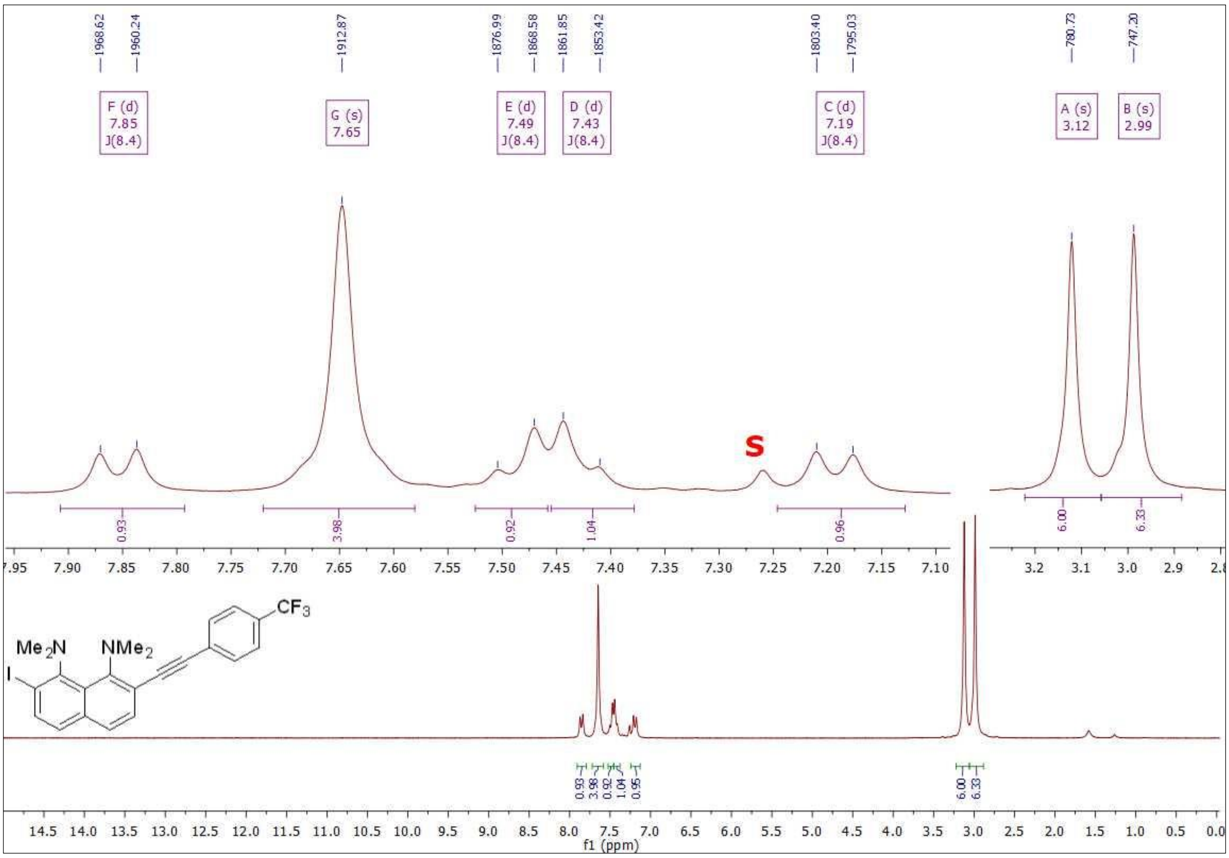

Figure S5. ^{1}H NMR spectrum of compound **7c** (250 MHz, $CDCl_3$).

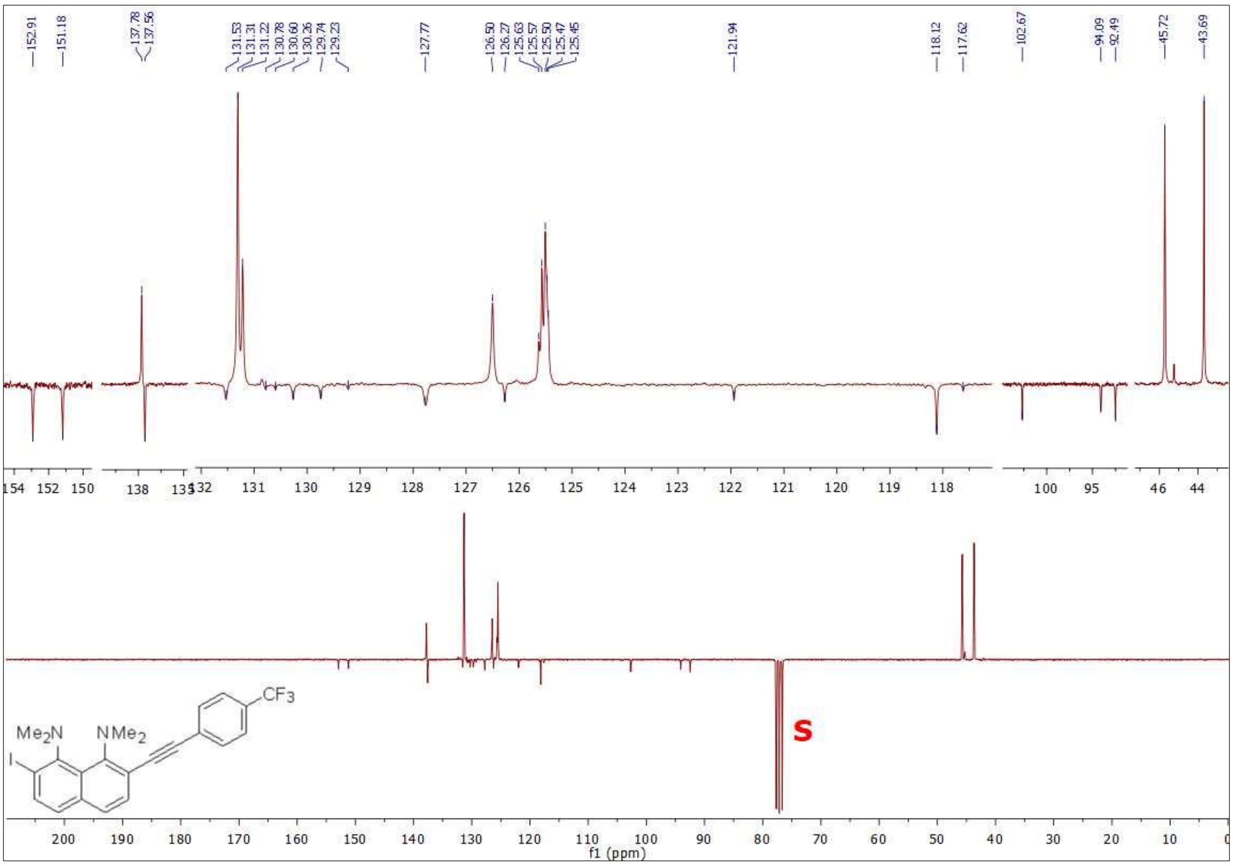

Figure S6. $^{13}C\{^{1}H\}$ APT-NMR spectrum of compound **7c** (62.9 MHz, $CDCl_3$).

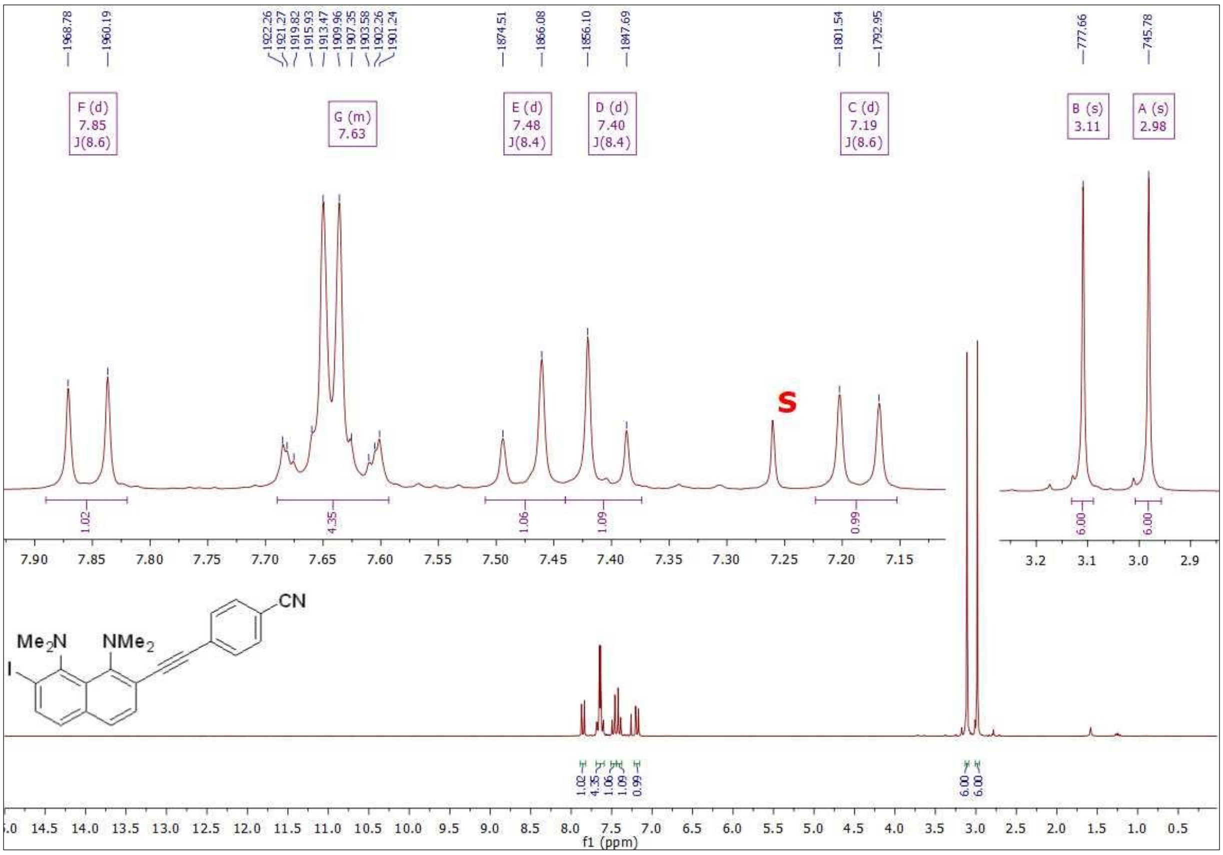

Figure S7. ^{1}H NMR spectrum of compound **7d** (250 MHz, $CDCl_3$).

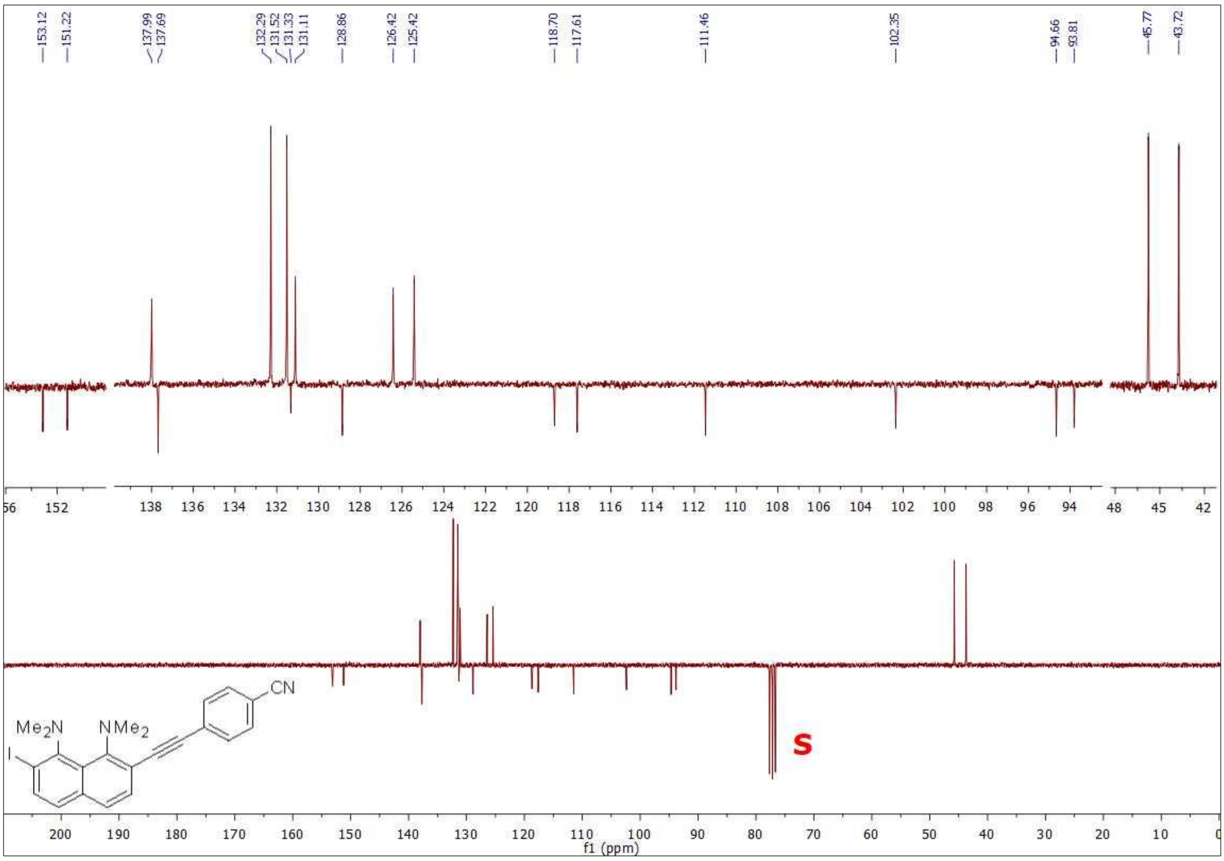

Figure S8. $^{13}C\{^{1}H\}$ APT-NMR spectrum of compound 7**d** (62.9 MHz, $CDCl_3$).

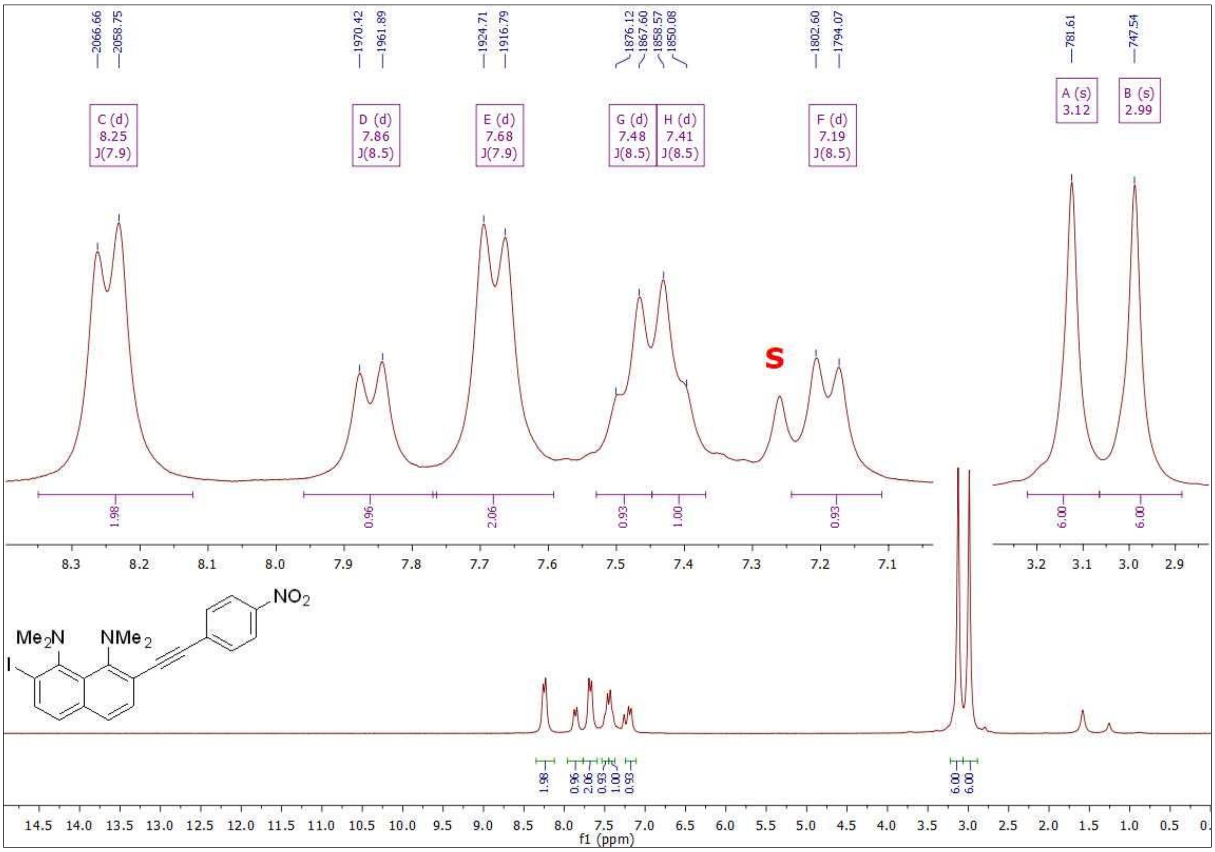

Figure S9. ^{1}H NMR spectrum of compound **7e** (250 MHz, $CDCl_3$).

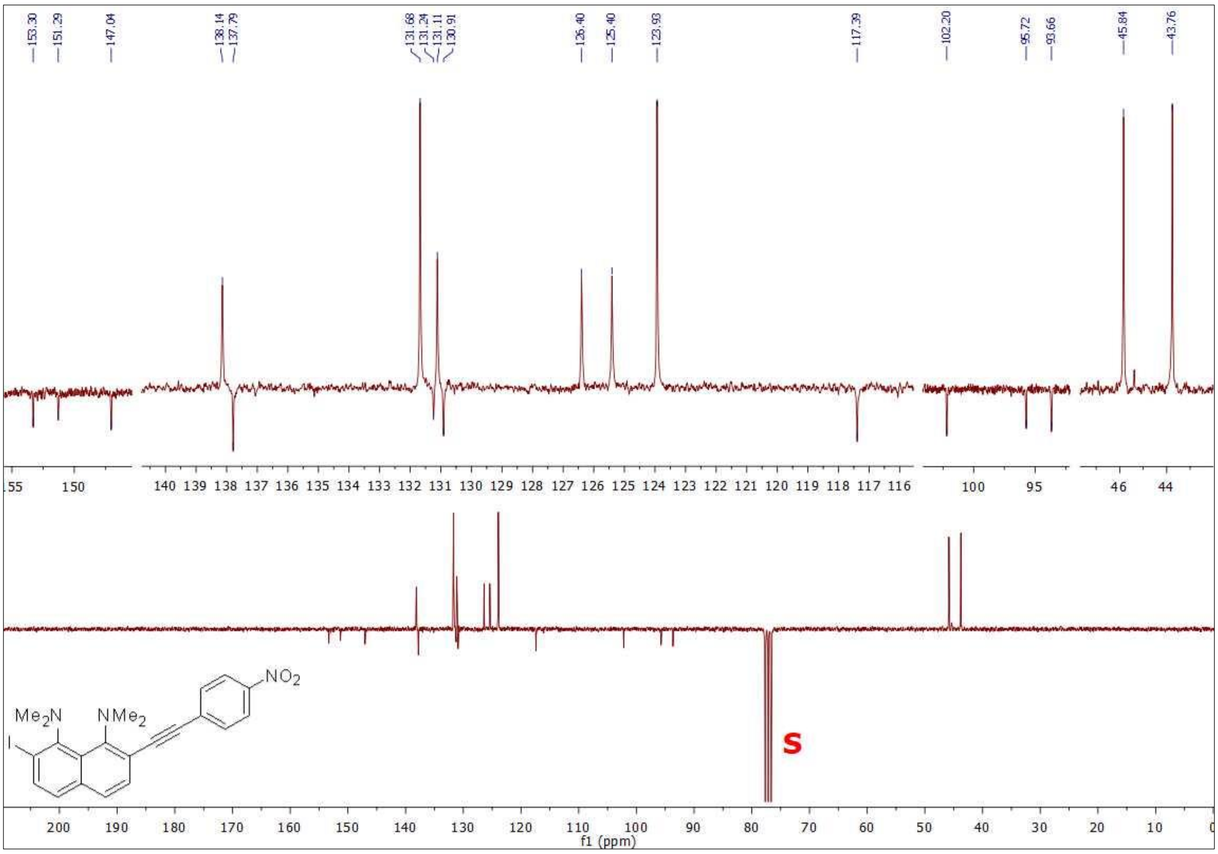

Figure S10. ^{13}C{^{1}H} APT-NMR spectrum of compound **7e** (62.9 MHz, $CDCl_3$).

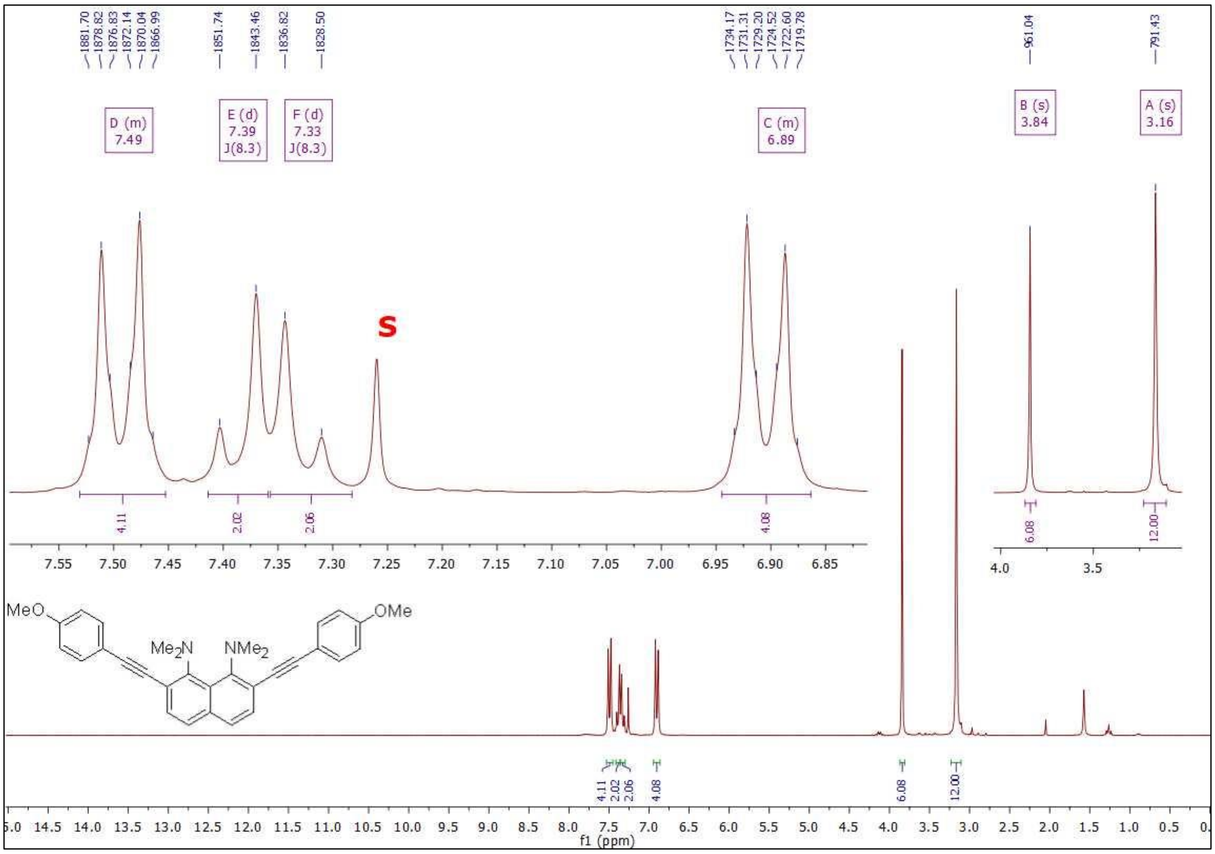

Figure S11. ^{1}H NMR spectrum of compound **9b** (250 MHz, $CDCl_3$).

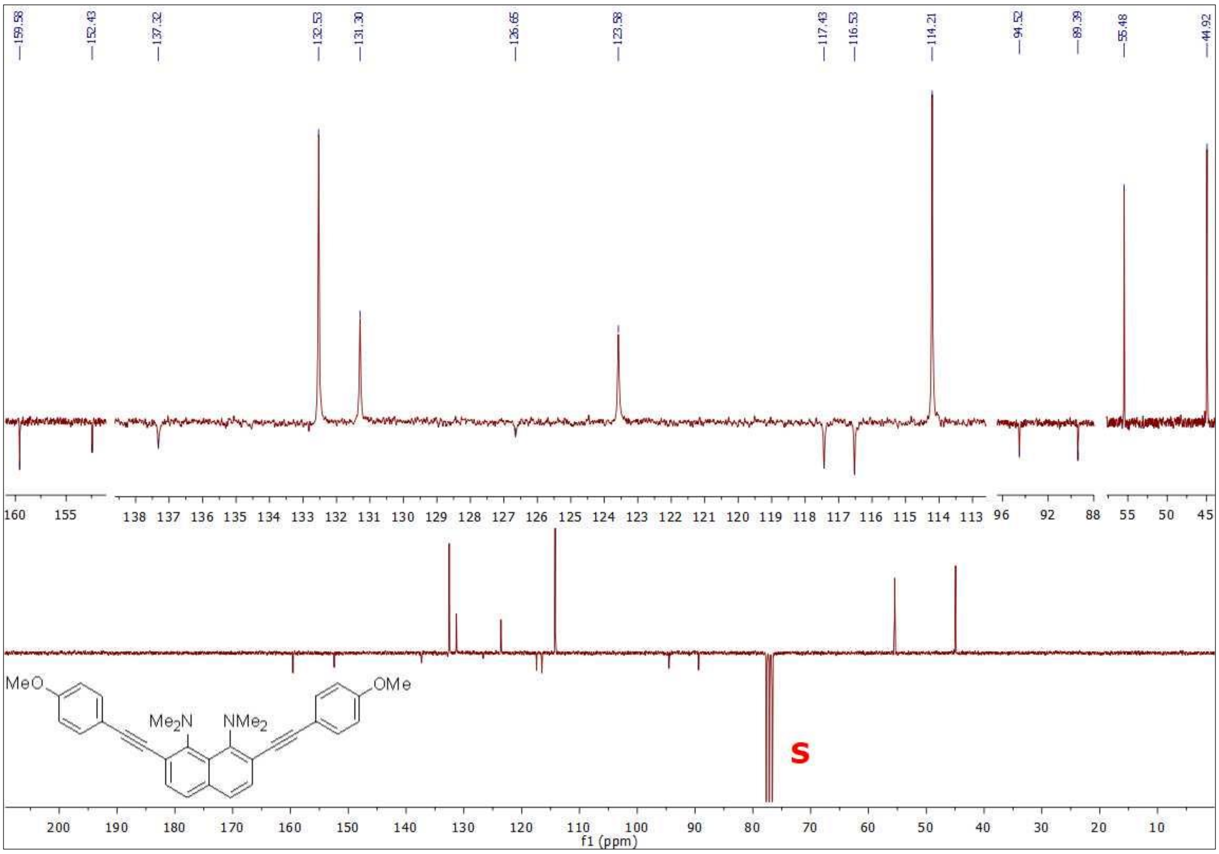

Figure S12. $^{13}C\{^{1}H\}$ APT-NMR spectrum of compound **9b** (62.9 MHz, $CDCl_3$).

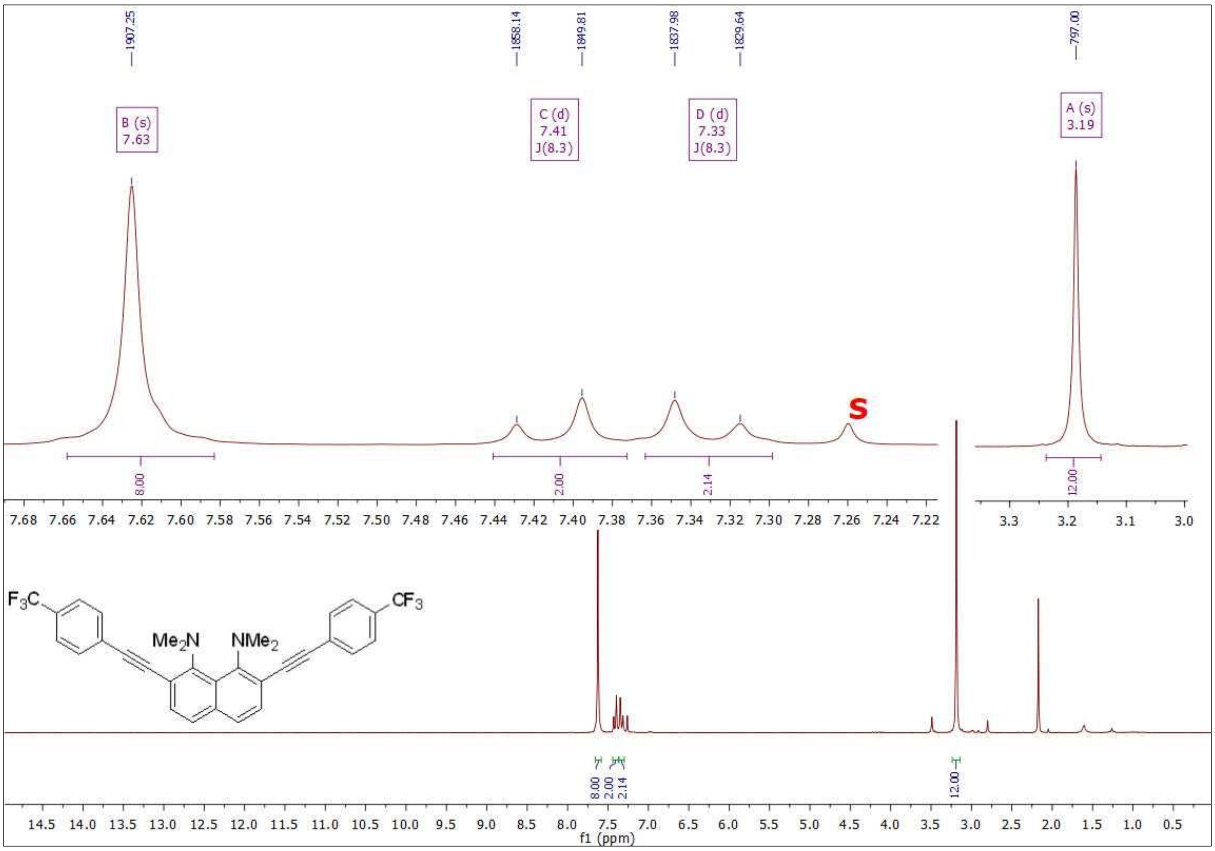

Figure S13. ^{1}H NMR spectrum of compound **9c** (250 MHz, $CDCl_3$).

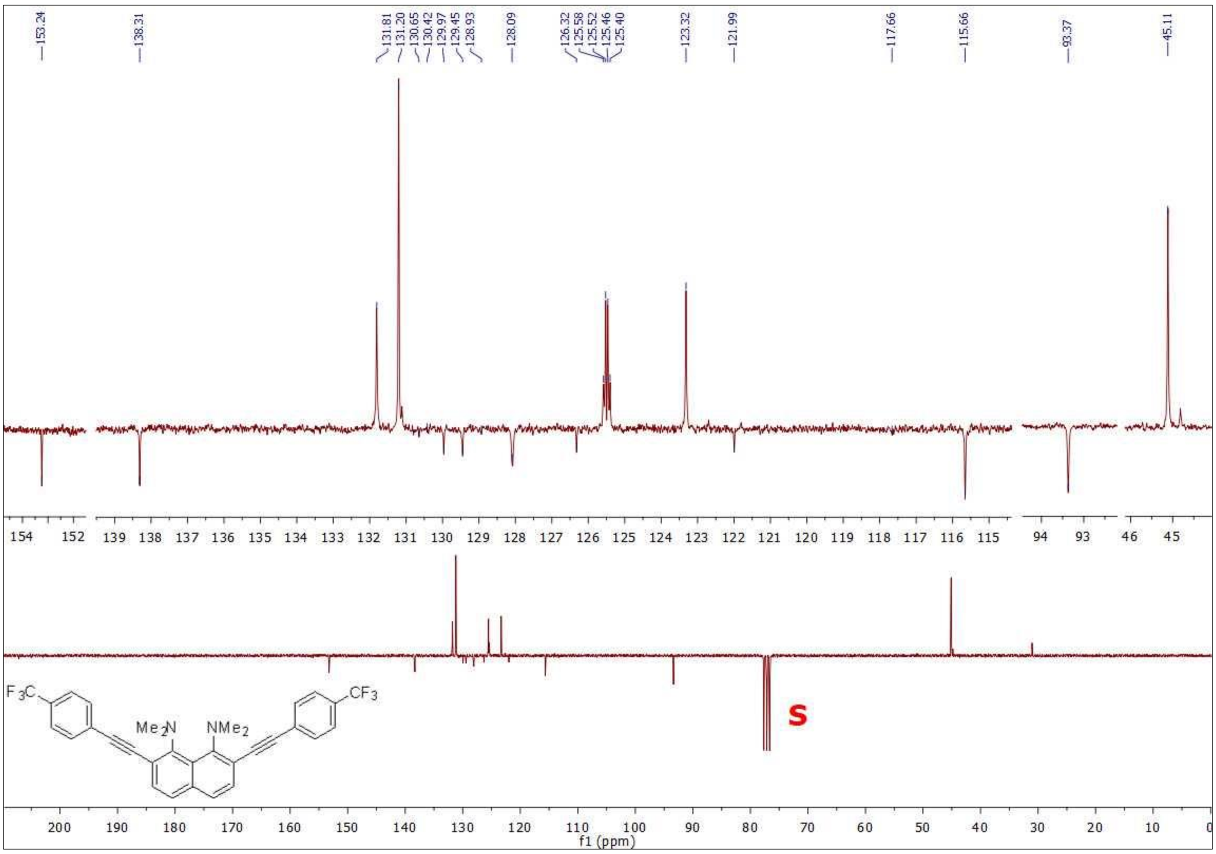

Figure S14. $^{13}C\{^{1}H\}$ APT-NMR spectrum of compound **9c** (62.9 MHz, $CDCl_3$).

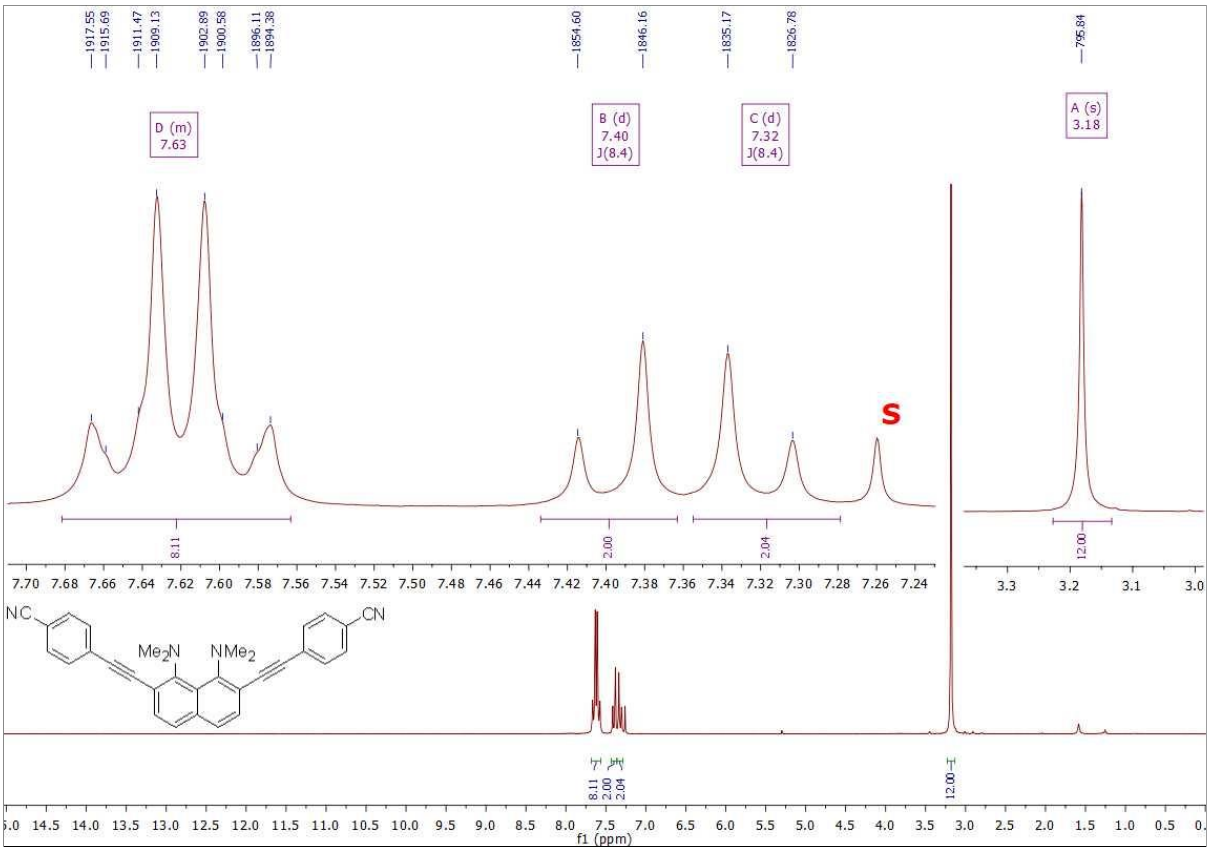

Figure S15. ^{1}H NMR spectrum of compound **9d** (250 MHz, $CDCl_3$).

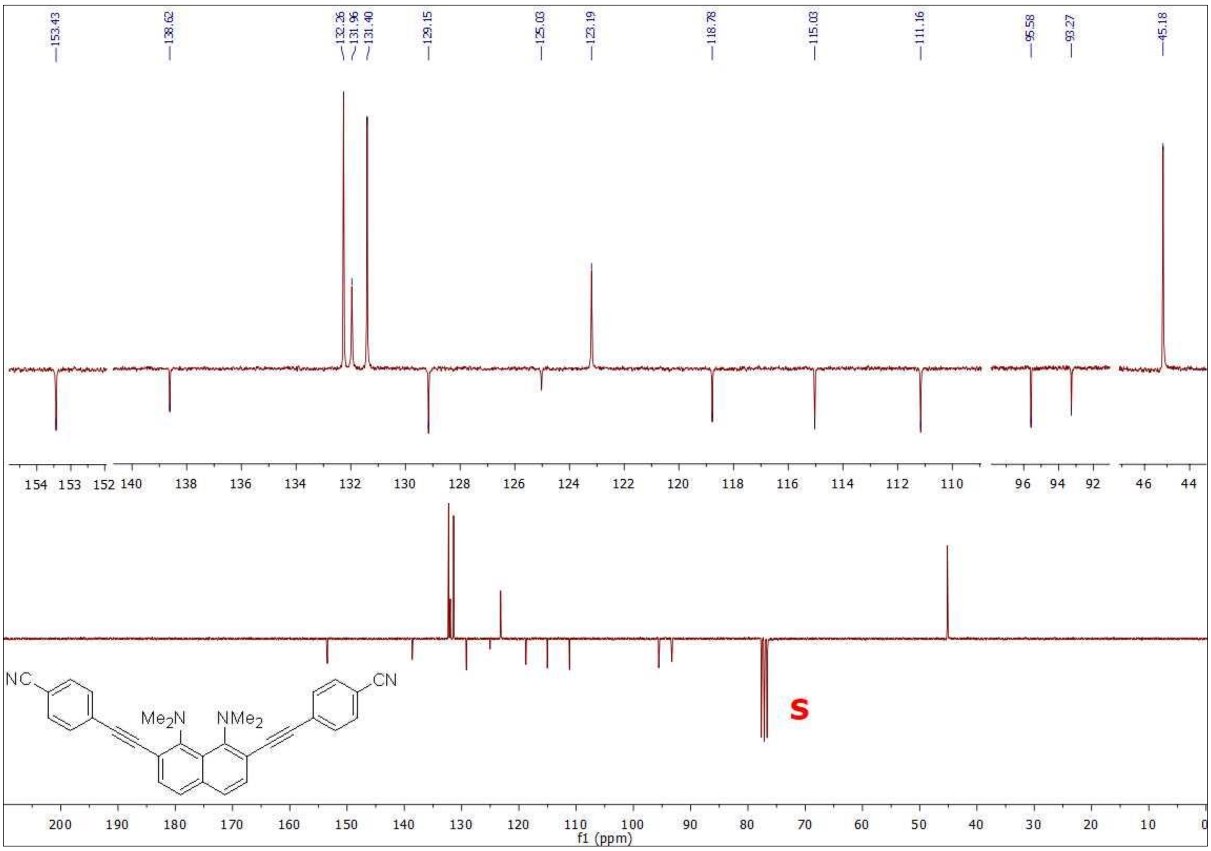

Figure S16. $^{13}C\{^{1}H\}$ APT-NMR spectrum of compound **9d** (62.9 MHz, $CDCl_3$).

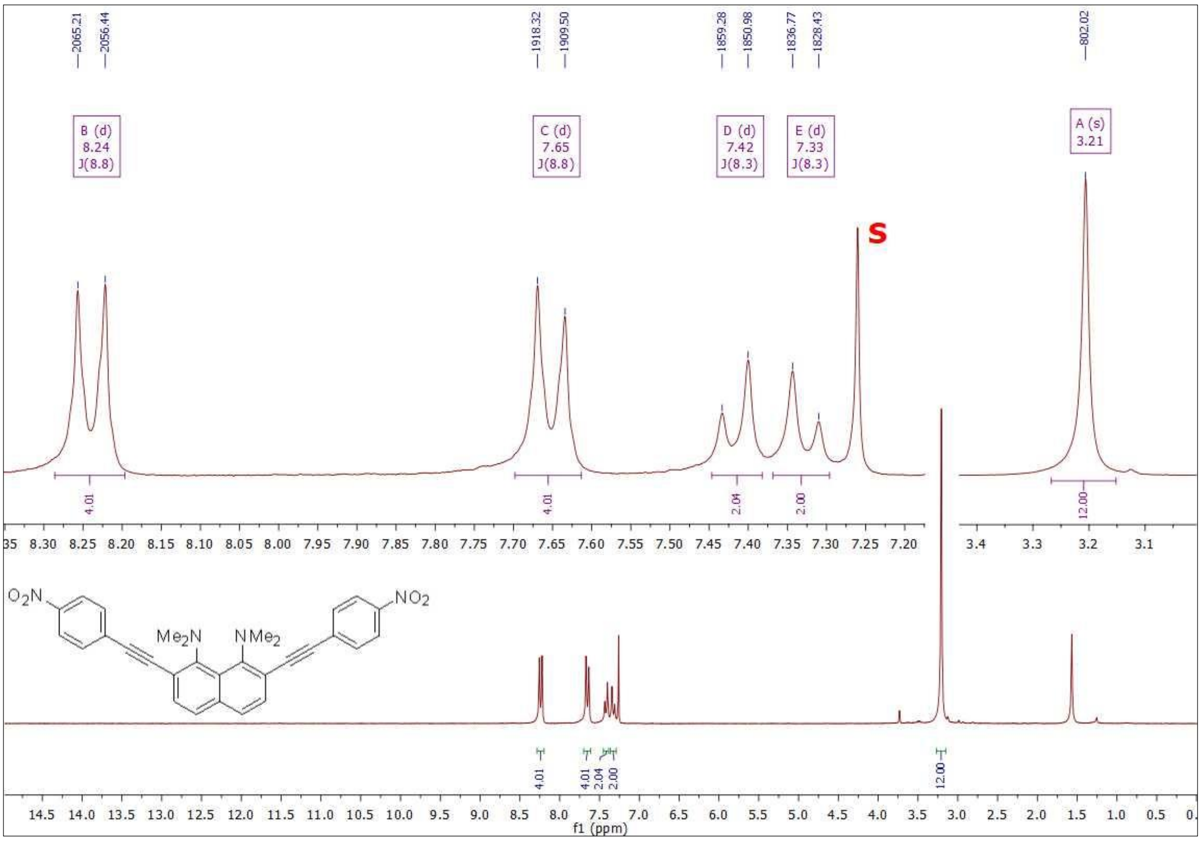

Figure S17. ^{1}H NMR spectrum of compound **9e** (250 MHz, $CDCl_3$).

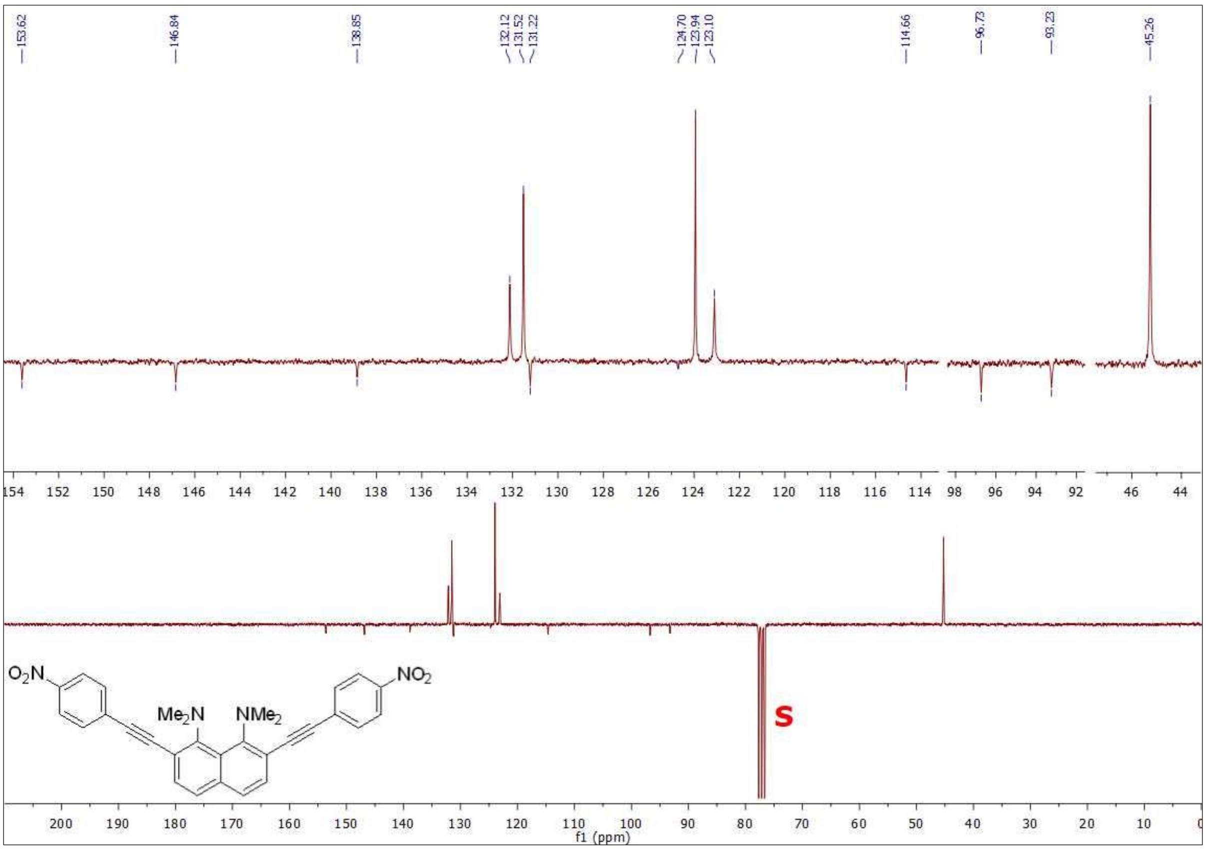

Figure S18. $^{13}C\{^1H\}$ APT-NMR spectrum of compound **9e** (62.9 MHz, $CDCl_3$).

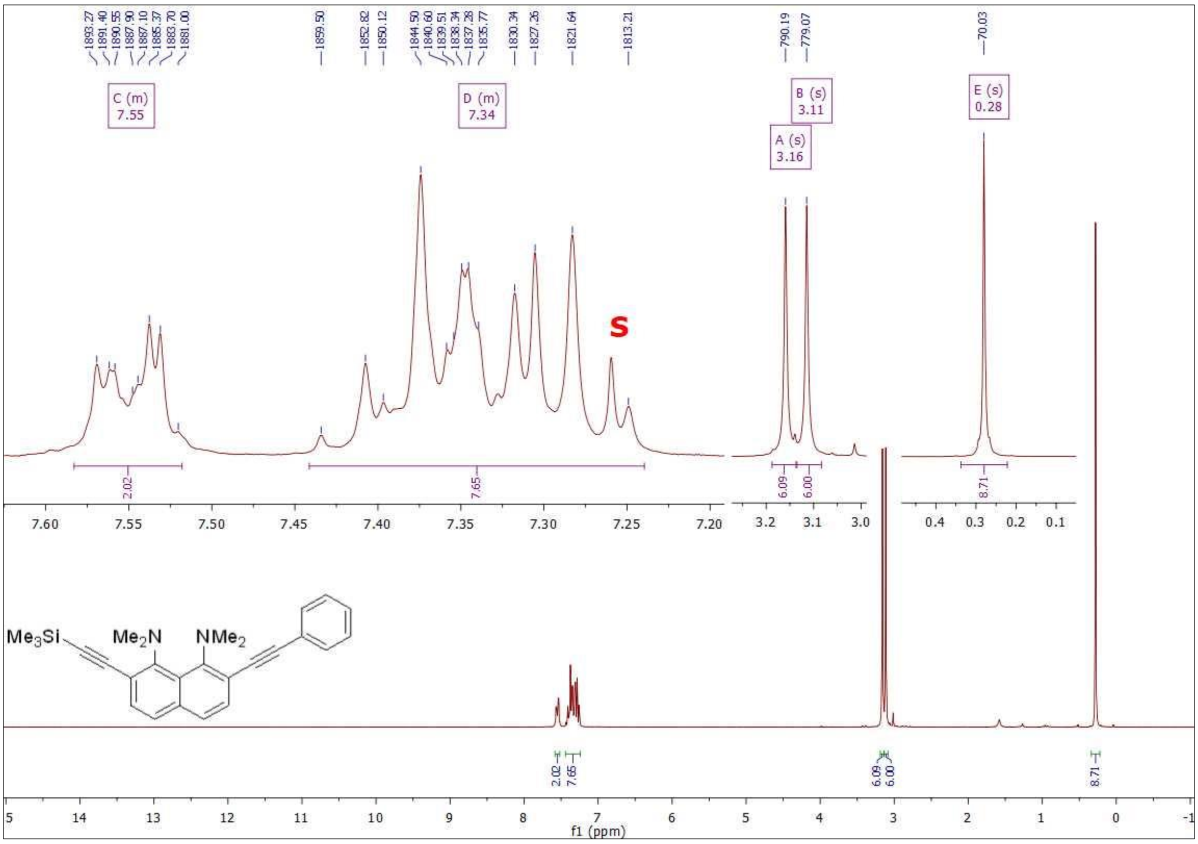

Figure S19. ^{1}H NMR spectrum of compound **10a** (250 MHz, $CDCl_3$).

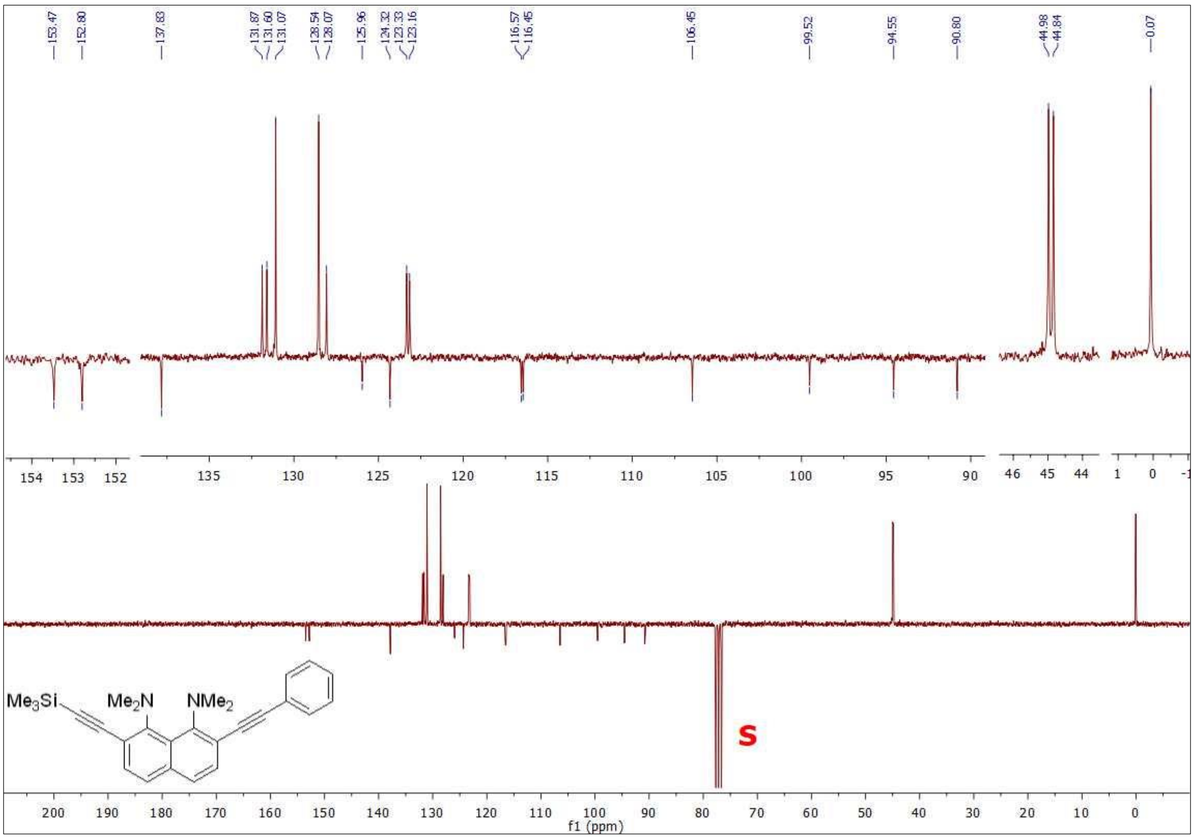

Figure S20. $^{13}C\{^{1}H\}$ APT-NMR spectrum of compound **10a** (62.9 MHz, $CDCl_3$).

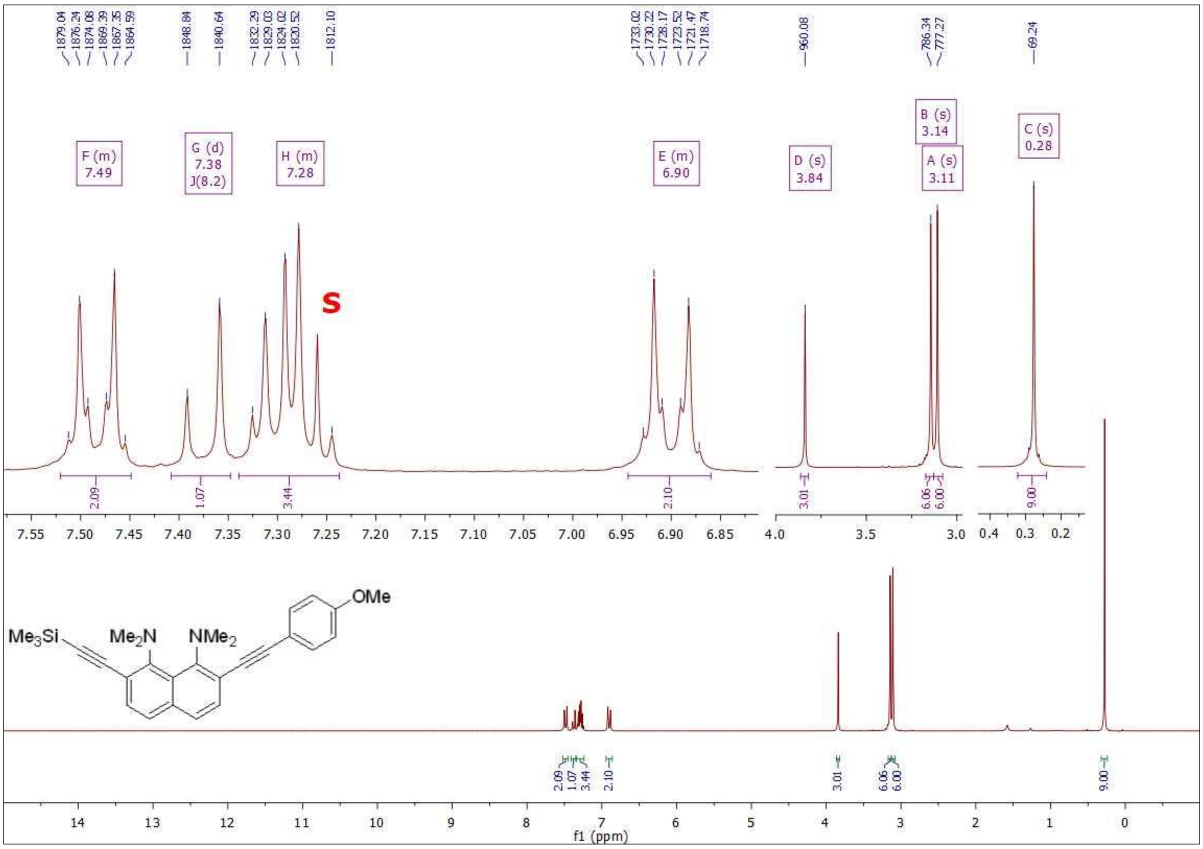

Figure S21. ^{1}H NMR spectrum of compound **10b** (250 MHz, $CDCl_3$).

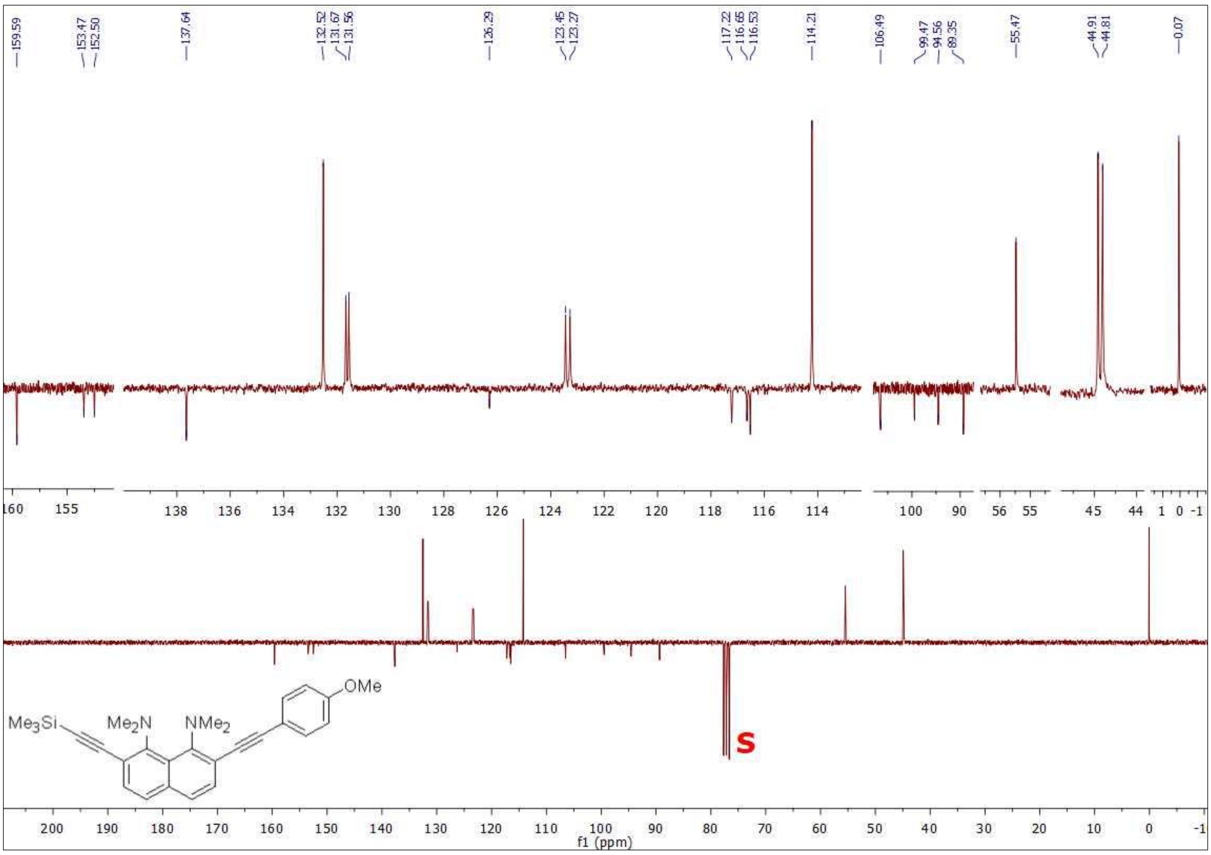

Figure S22. $^{13}C\{^{1}H\}$ APT-NMR spectrum of compound **10b** (62.9 MHz, $CDCl_3$).

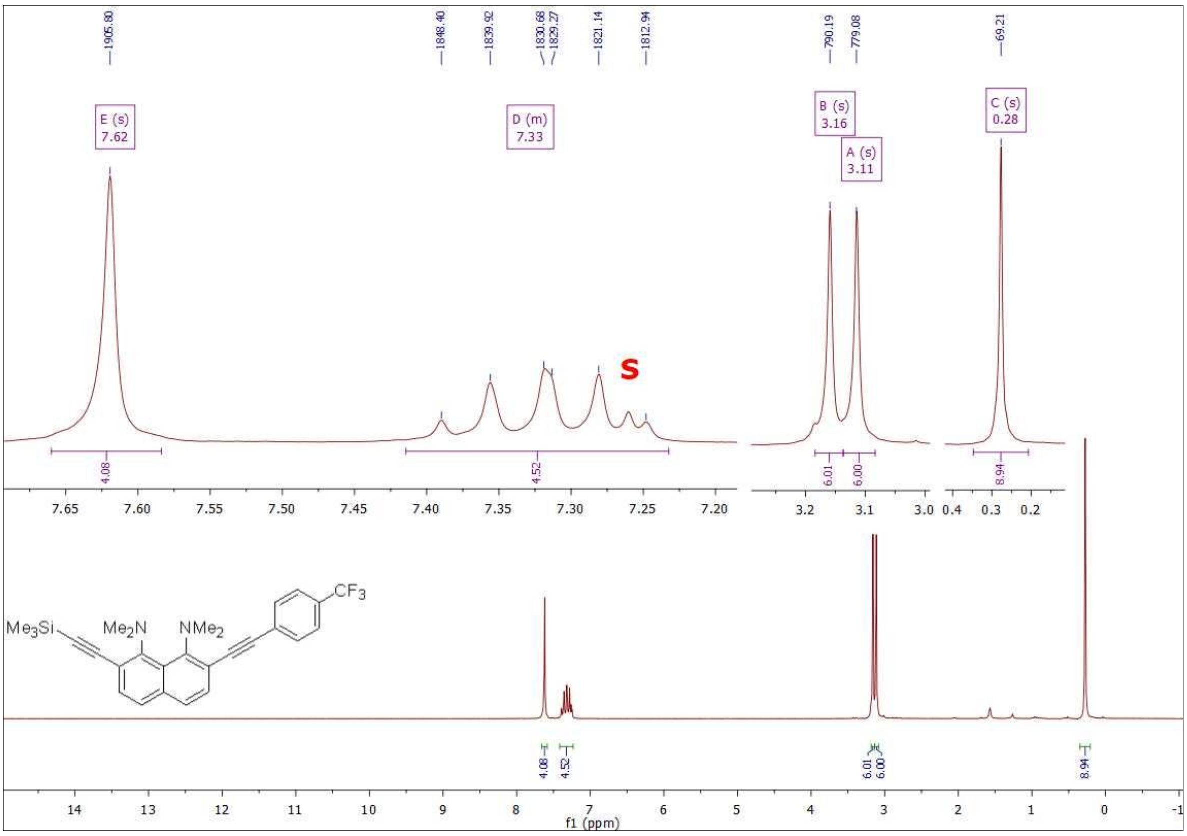

Figure S23. ^{1}H NMR spectrum of compound **10c** (250 MHz, $CDCl_3$).

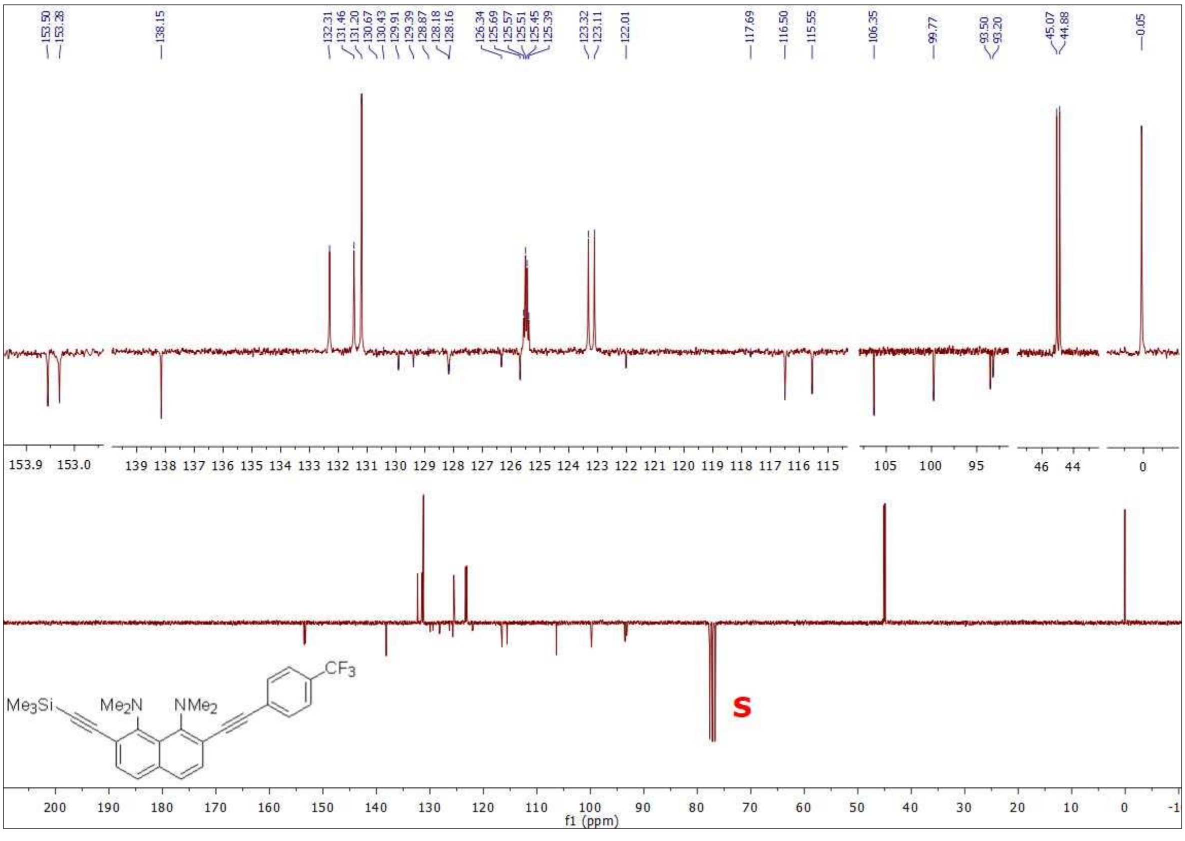

Figure S24. $^{13}C\{^{1}H\}$ APT-NMR spectrum of compound **10c** (62.9 MHz, $CDCl_3$).

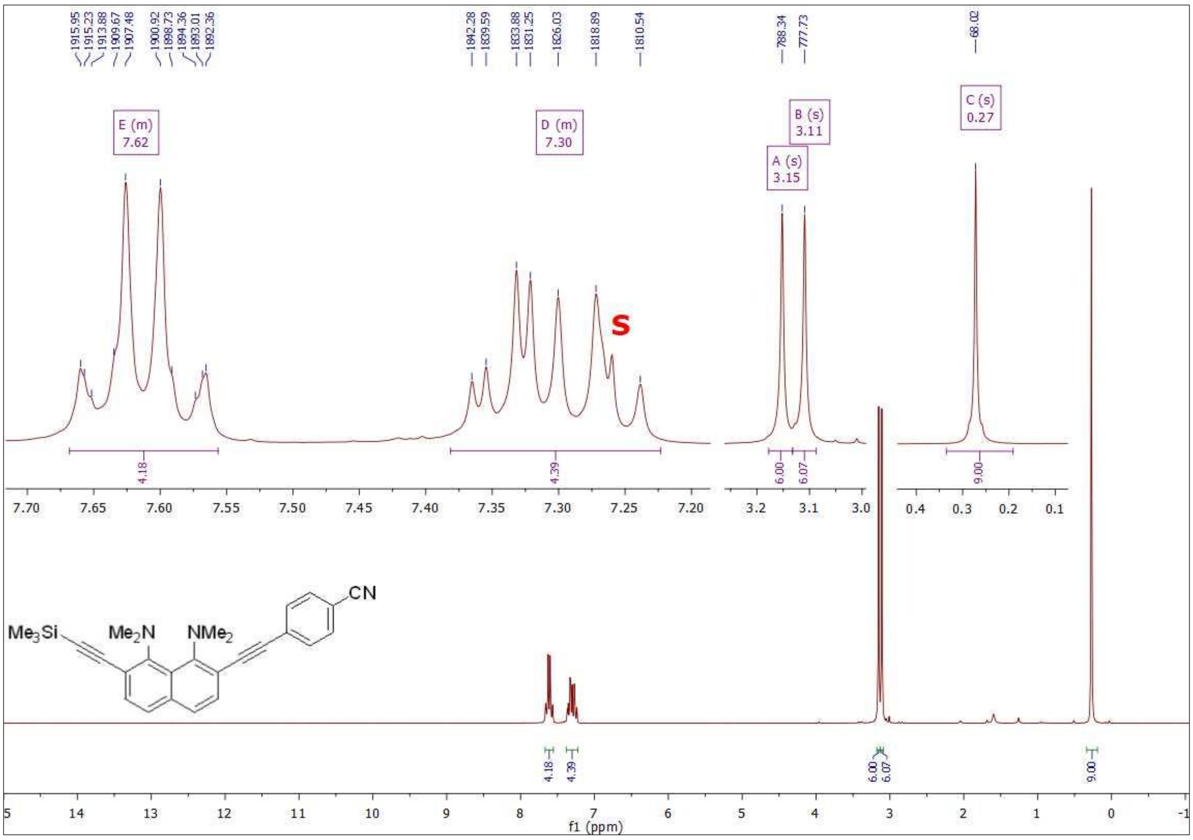

Figure S25. ^{1}H NMR spectrum of compound **10d** (250 MHz, $CDCl_3$).

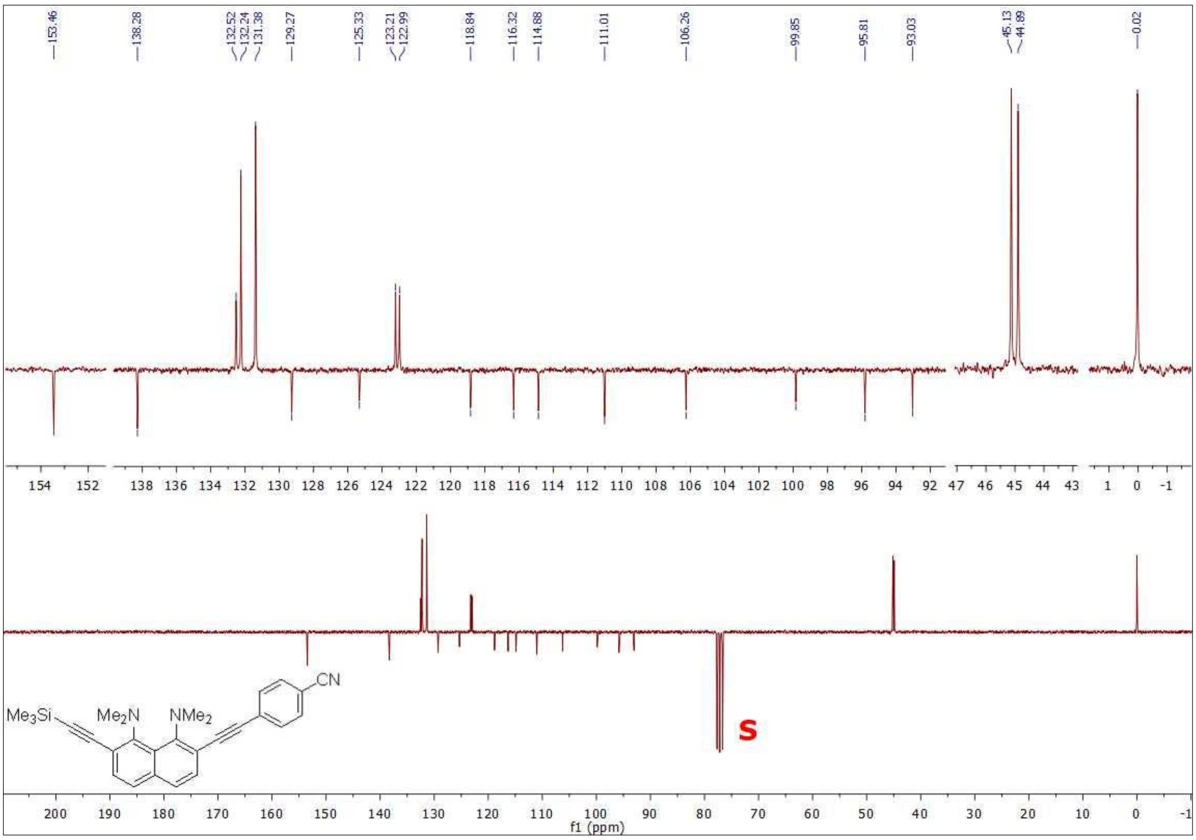

Figure S26. $^{13}C\{^{1}H\}$ APT-NMR spectrum of compound **10d** (62.9 MHz, $CDCl_3$).

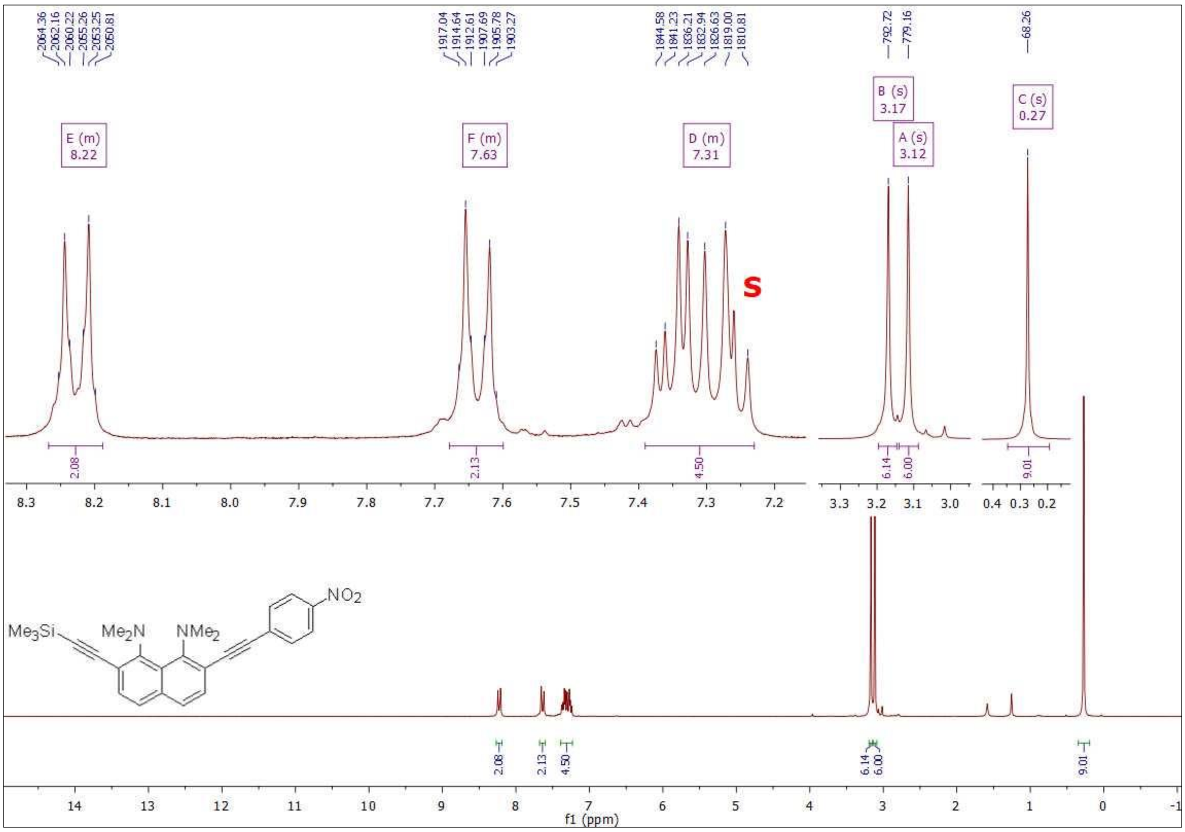

Figure S27. ^{1}H NMR spectrum of compound **10e** (250 MHz, $CDCl_3$).

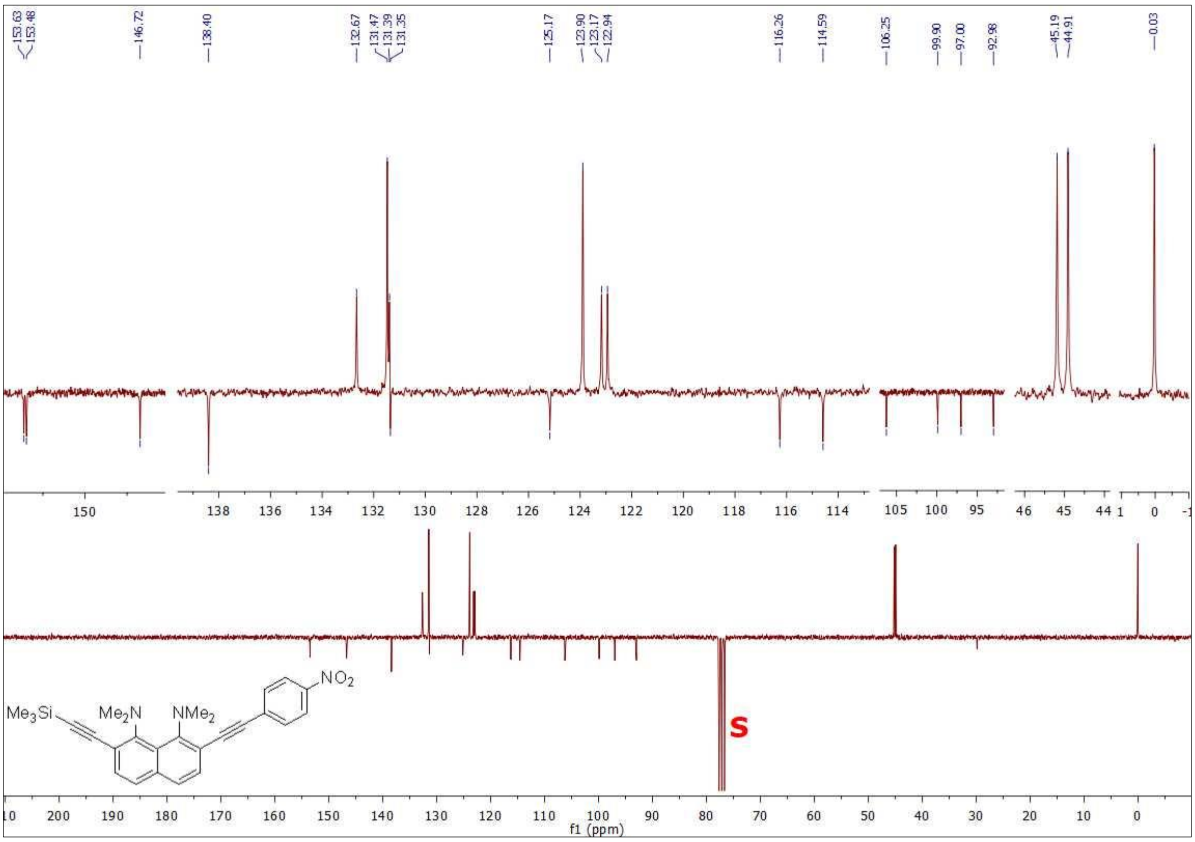

Figure S28. $^{13}C\{^{1}H\}$ APT-NMR spectrum of compound **10e** (62.9 MHz, $CDCl_3$).

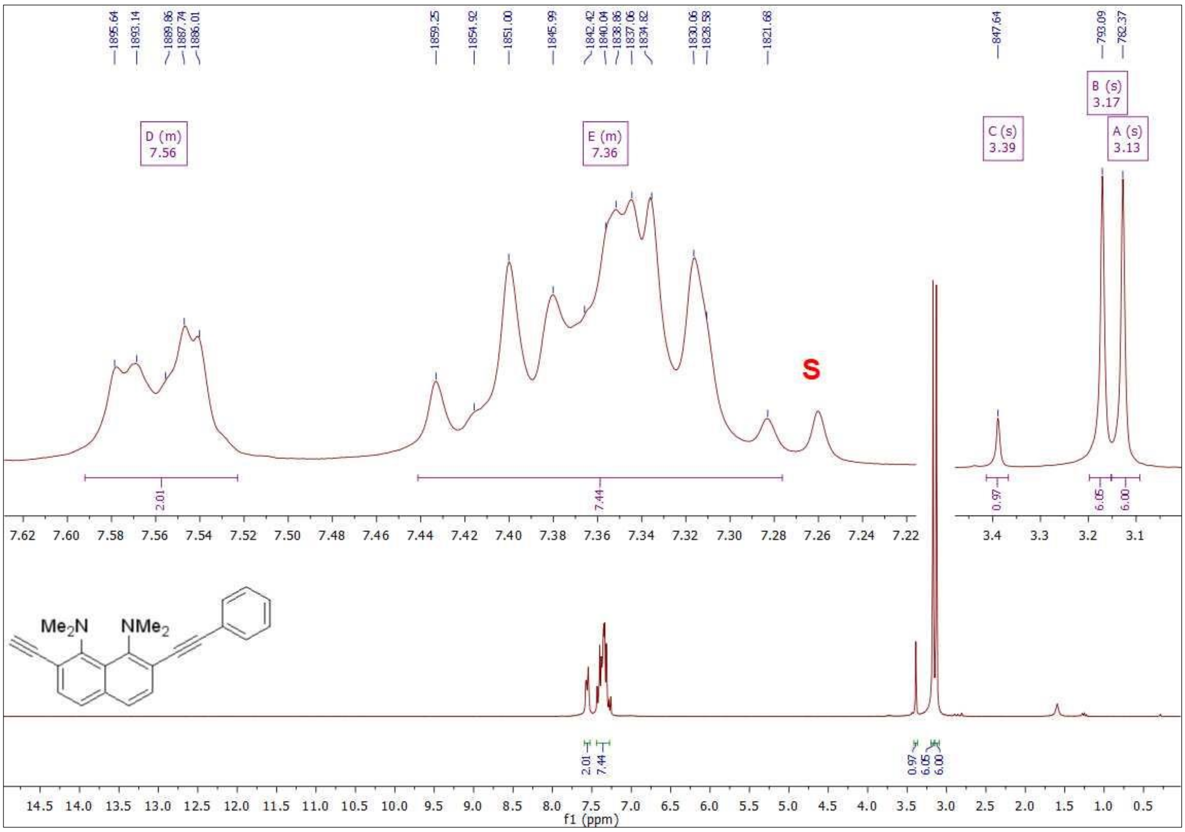

Figure S29. ^{1}H NMR spectrum of compound **6a** (250 MHz, $CDCl_3$).

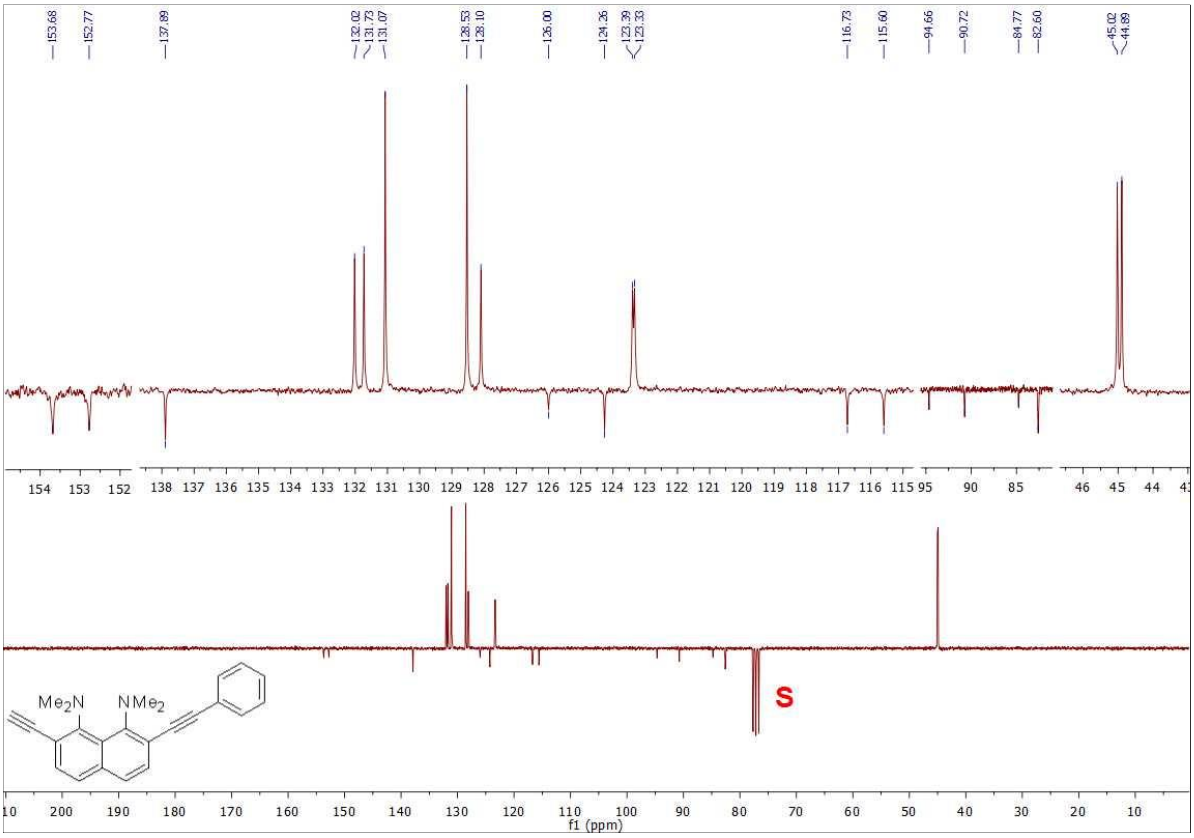

Figure S30. $^{13}C\{^{1}H\}$ APT-NMR spectrum of compound **6a** (62.9 MHz, $CDCl_3$).

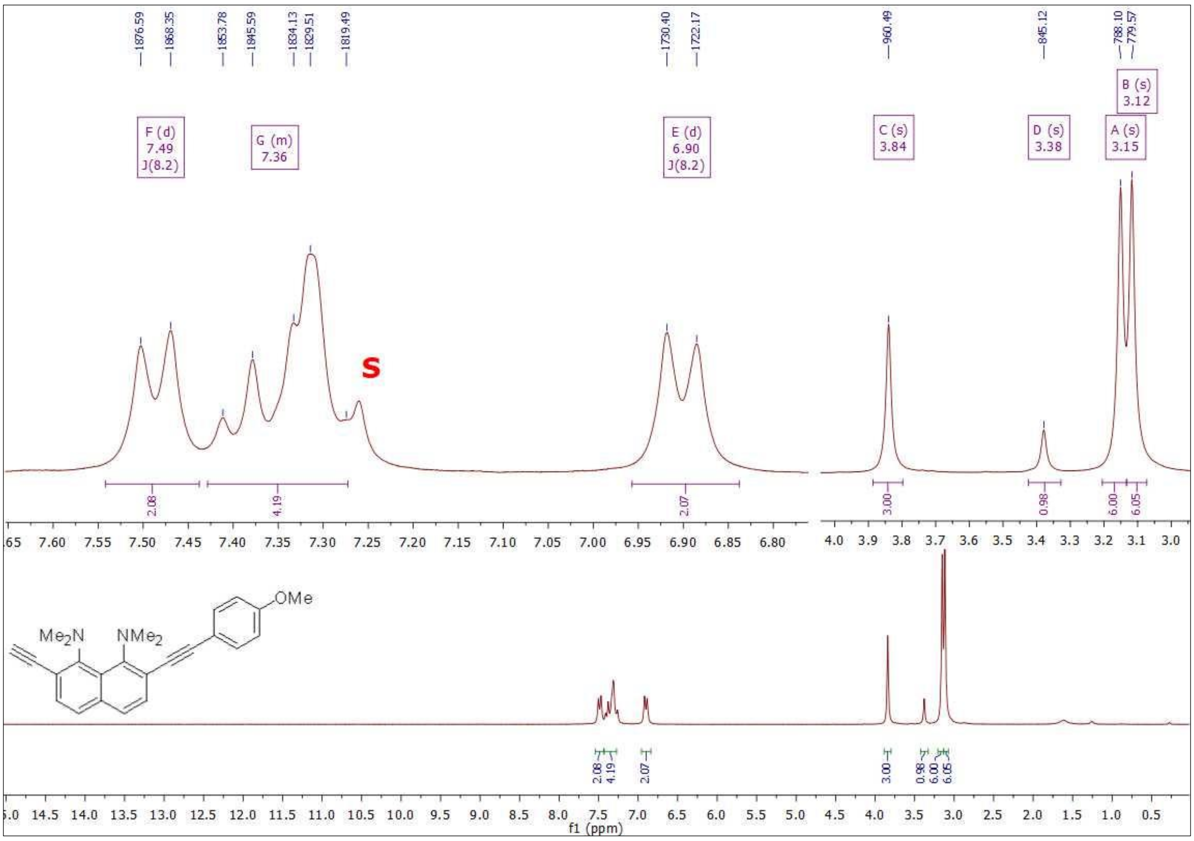

Figure S31. ^{1}H NMR spectrum of compound **6b** (250 MHz, $CDCl_3$).

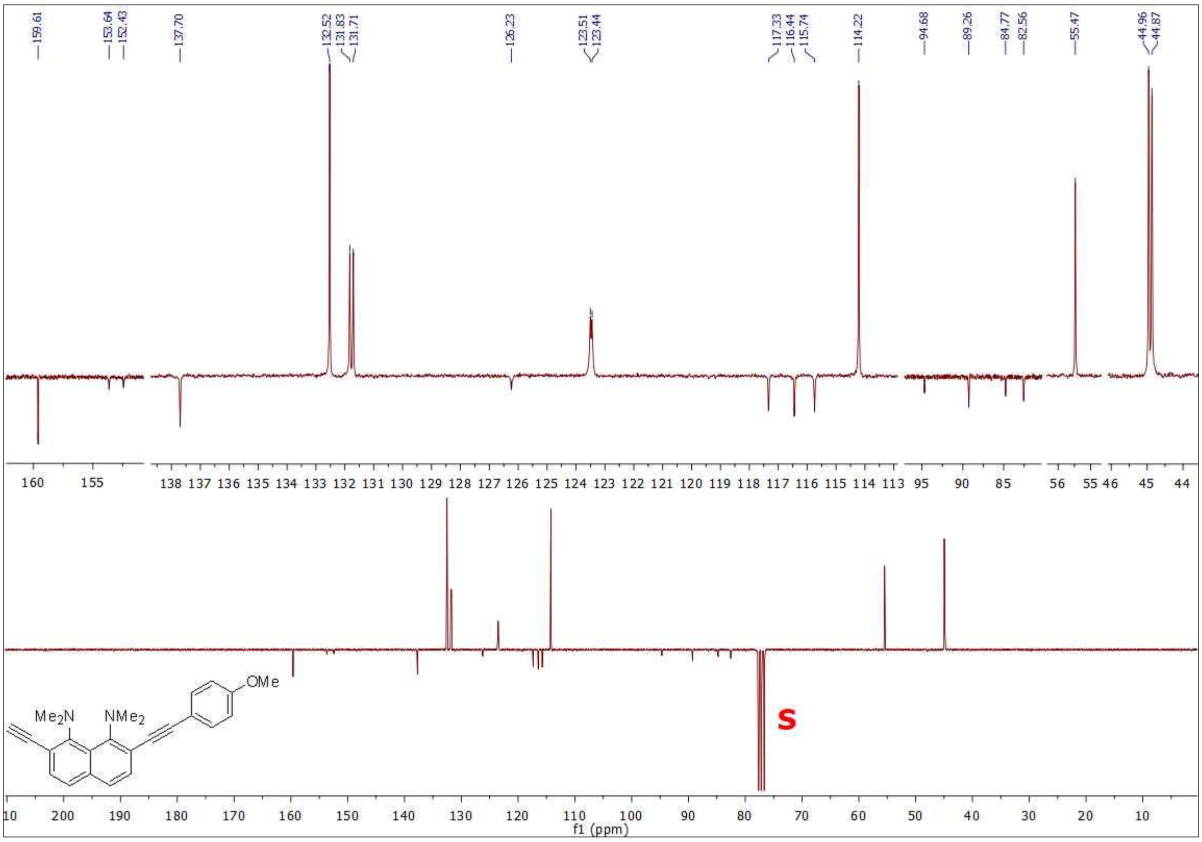

Figure S32. $^{13}C\{^{1}H\}$ APT-NMR spectrum of compound **6b** (62.9 MHz, $CDCl_3$).

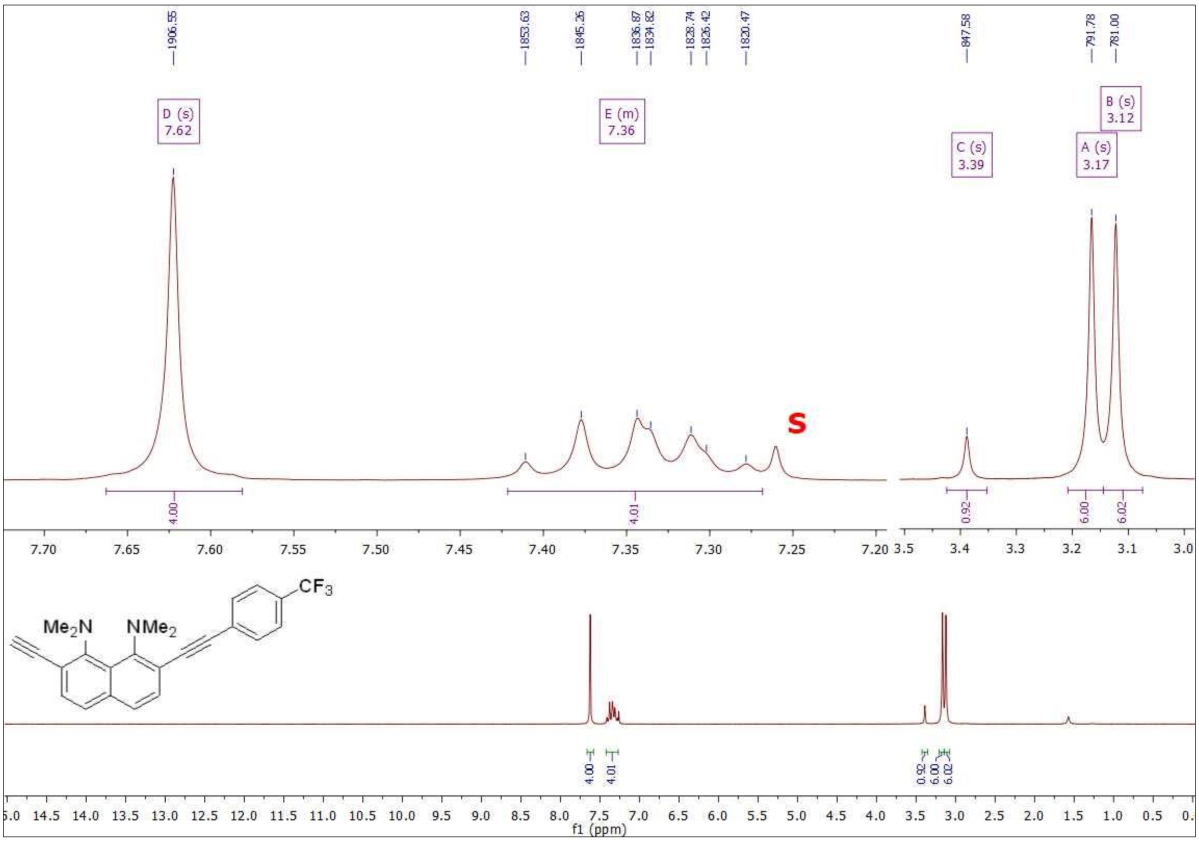

Figure S33. ^{1}H NMR spectrum of compound **6c** (250 MHz, $CDCl_3$).

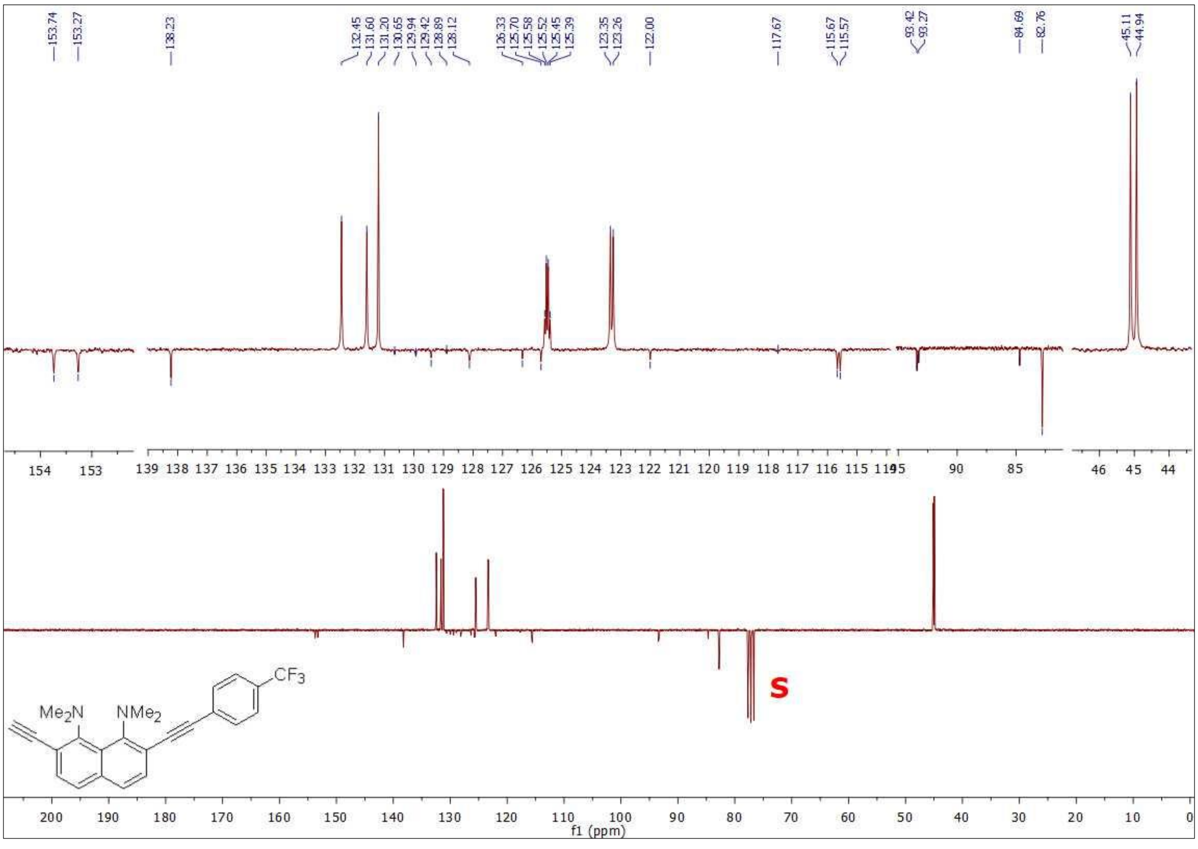

Figure S34. $^{13}C\{^{1}H\}$ APT-NMR spectrum of compound **6c** (62.9 MHz, $CDCl_3$).

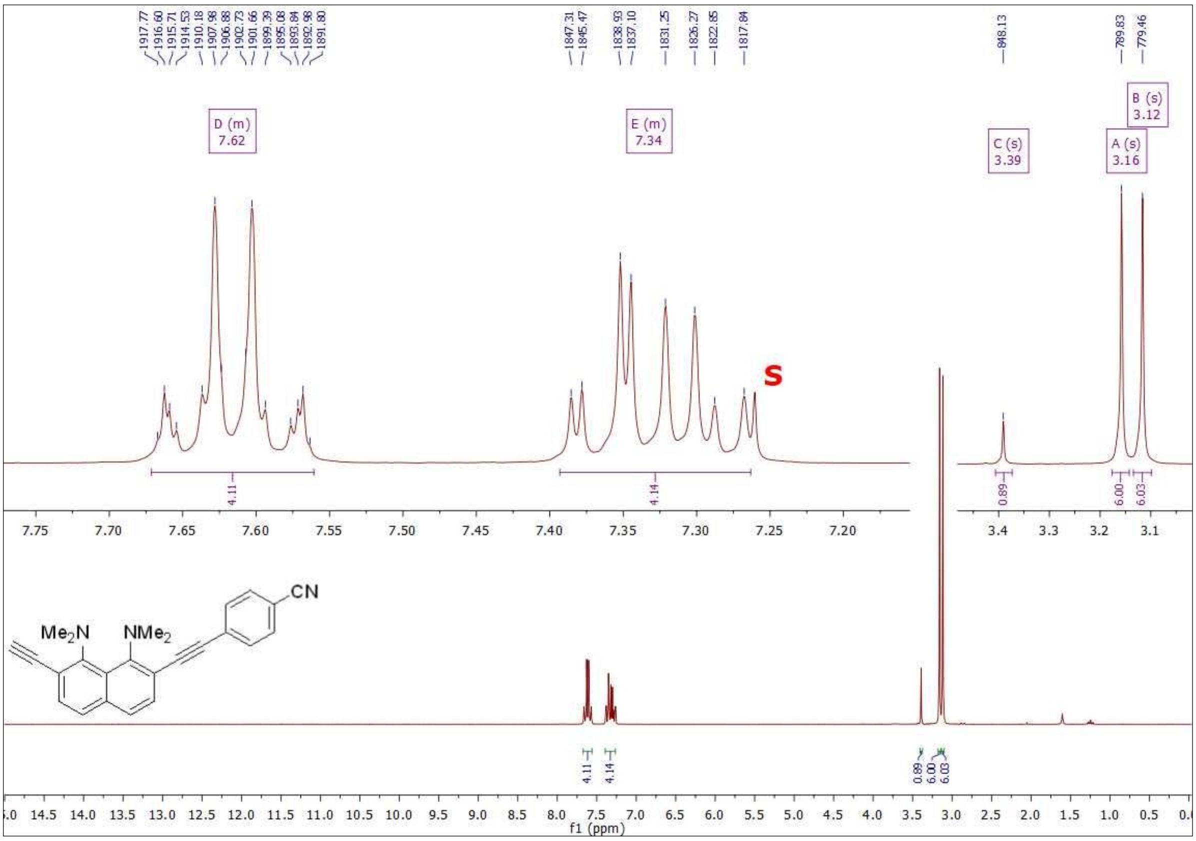

Figure S35. ^{1}H NMR spectrum of compound **6d** (250 MHz, $CDCl_3$).

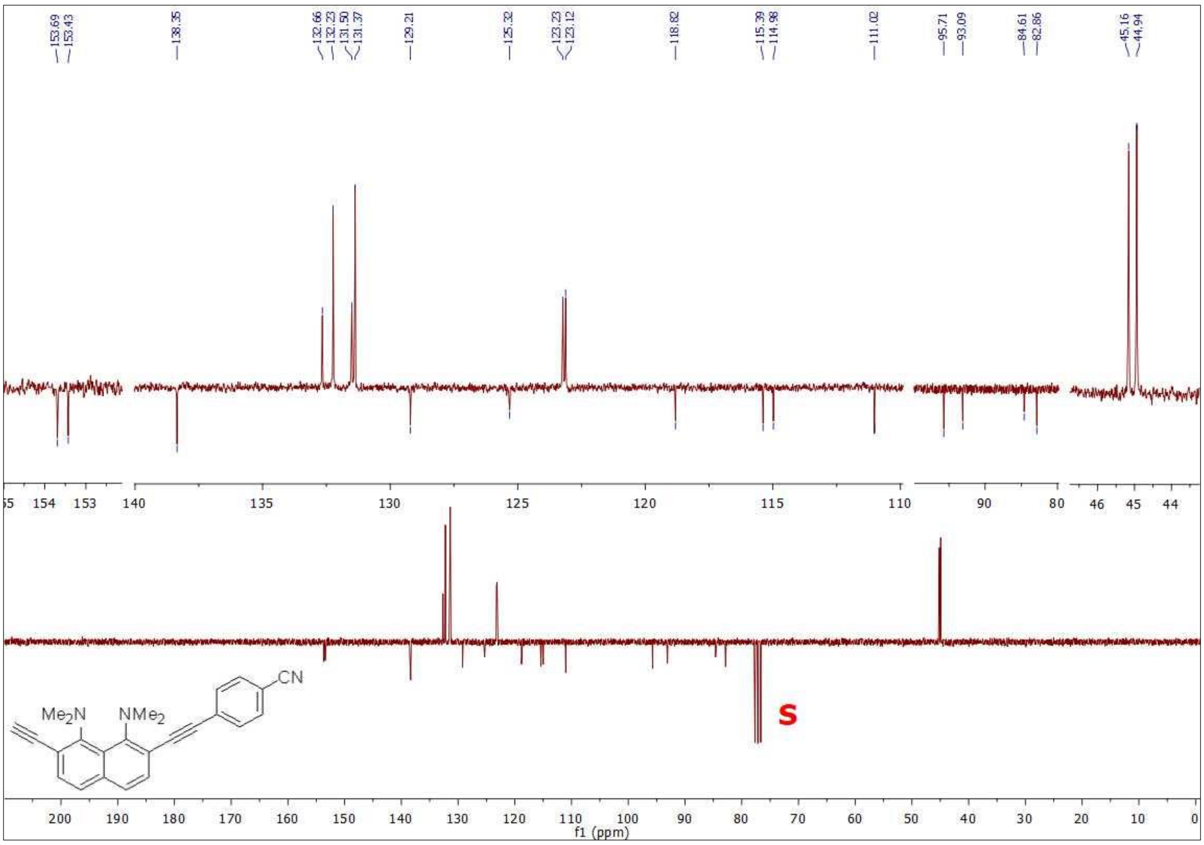

Figure S36. $^{13}C\{^{1}H\}$ APT-NMR spectrum of compound **6d** (62.9 MHz, $CDCl_3$).

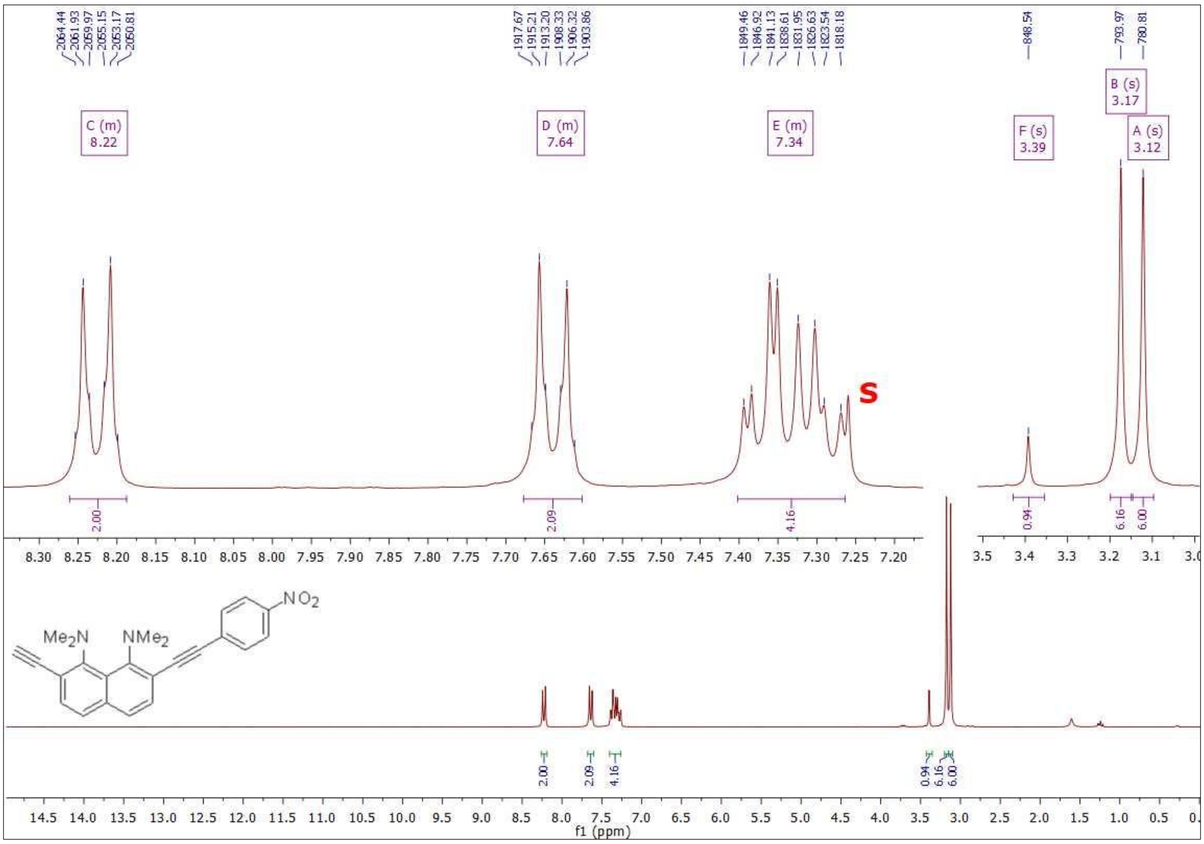

Figure S37. ^{1}H NMR spectrum of compound **6e** (250 MHz, $CDCl_3$).

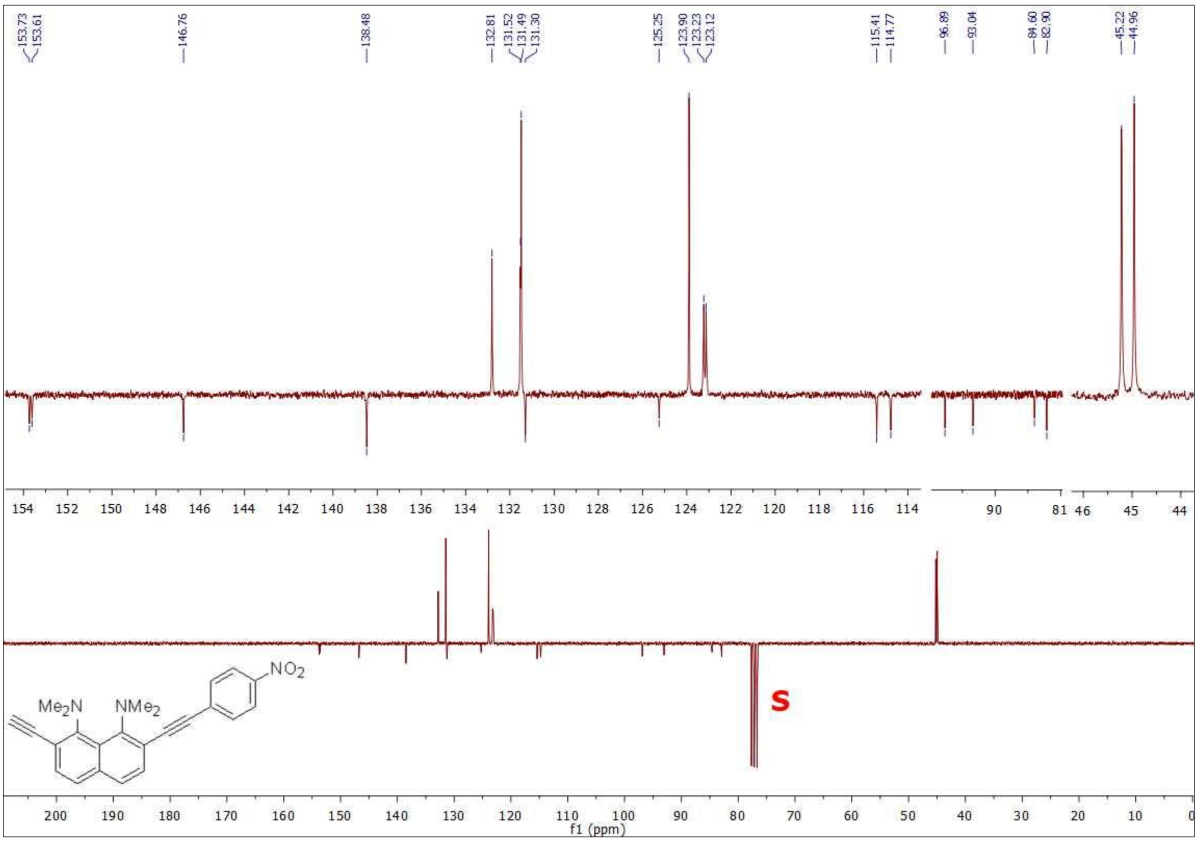

Figure S38. $^{13}C\{^{1}H\}$ APT-NMR spectrum of compound **6e** (62.9 MHz, $CDCl_3$).

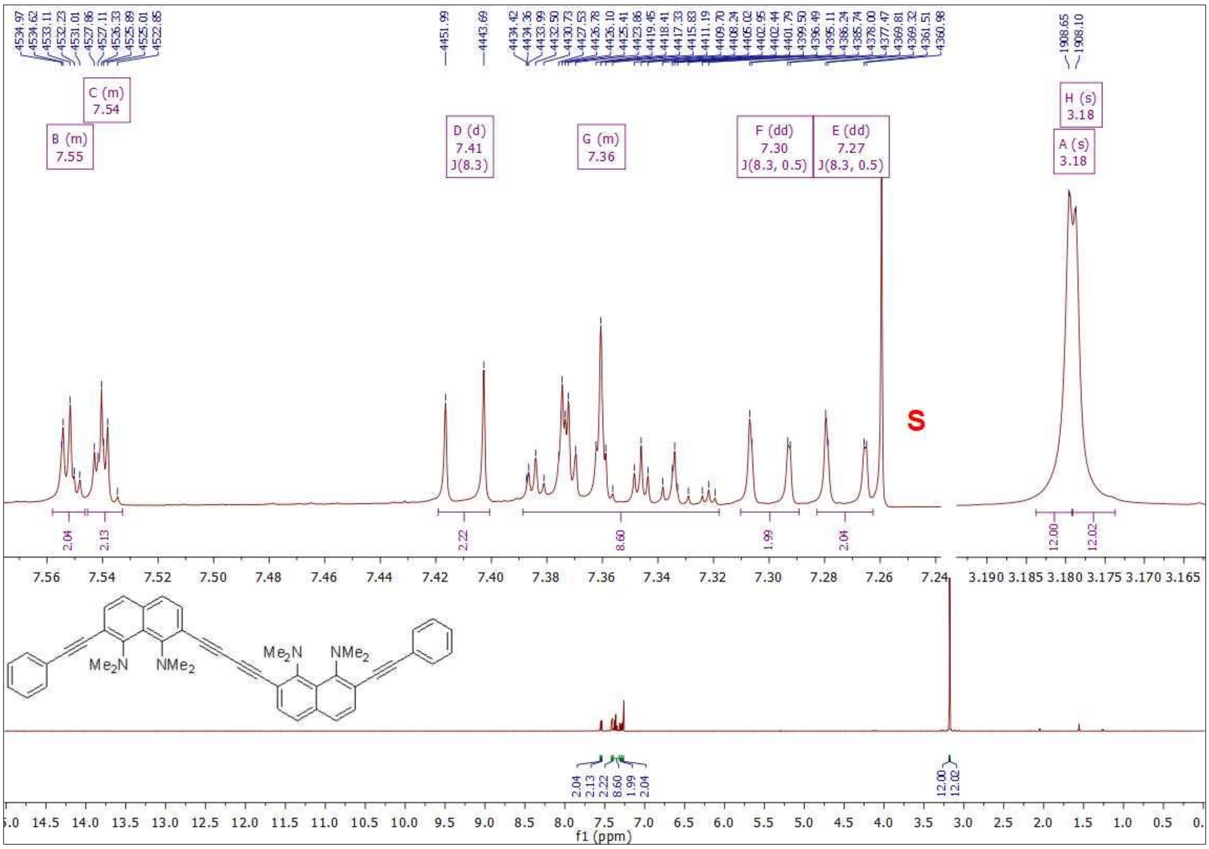

Figure S39. ^{1}H NMR spectrum of compound **5a** (600 MHz, $CDCl_3$).

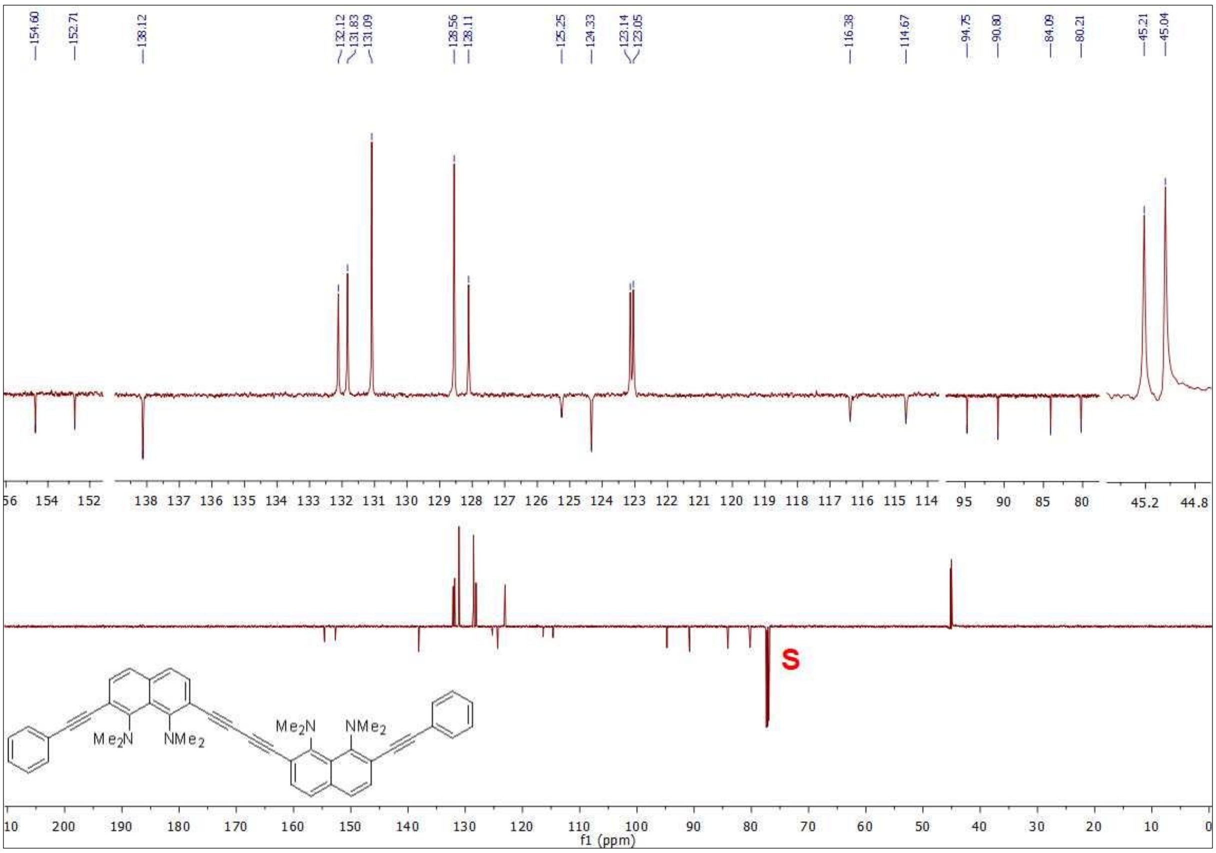

Figure S40. $^{13}C\{^1H\}$ APT-NMR spectrum of compound **5a** (150 MHz, $CDCl_3$).

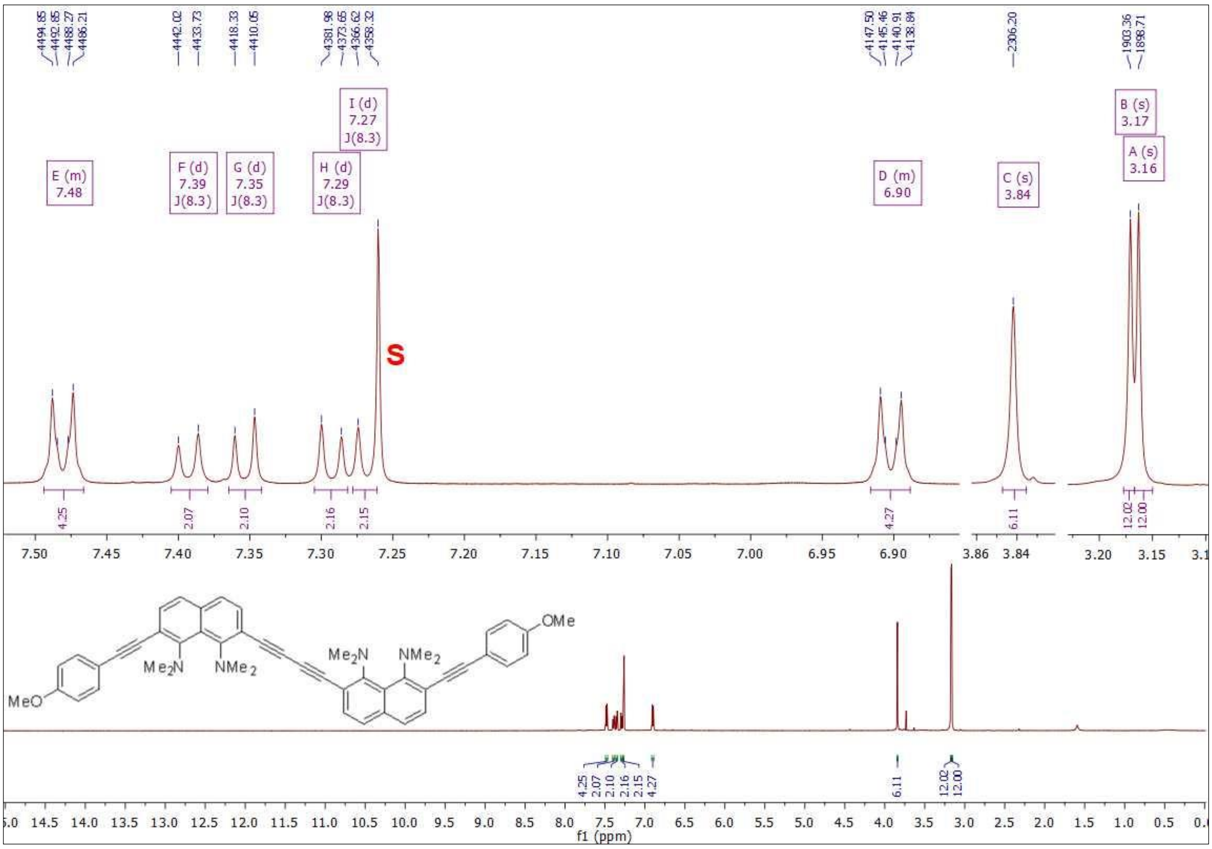

Figure S41. ^{1}H NMR spectrum of compound **5b** (600 MHz, $CDCl_3$).

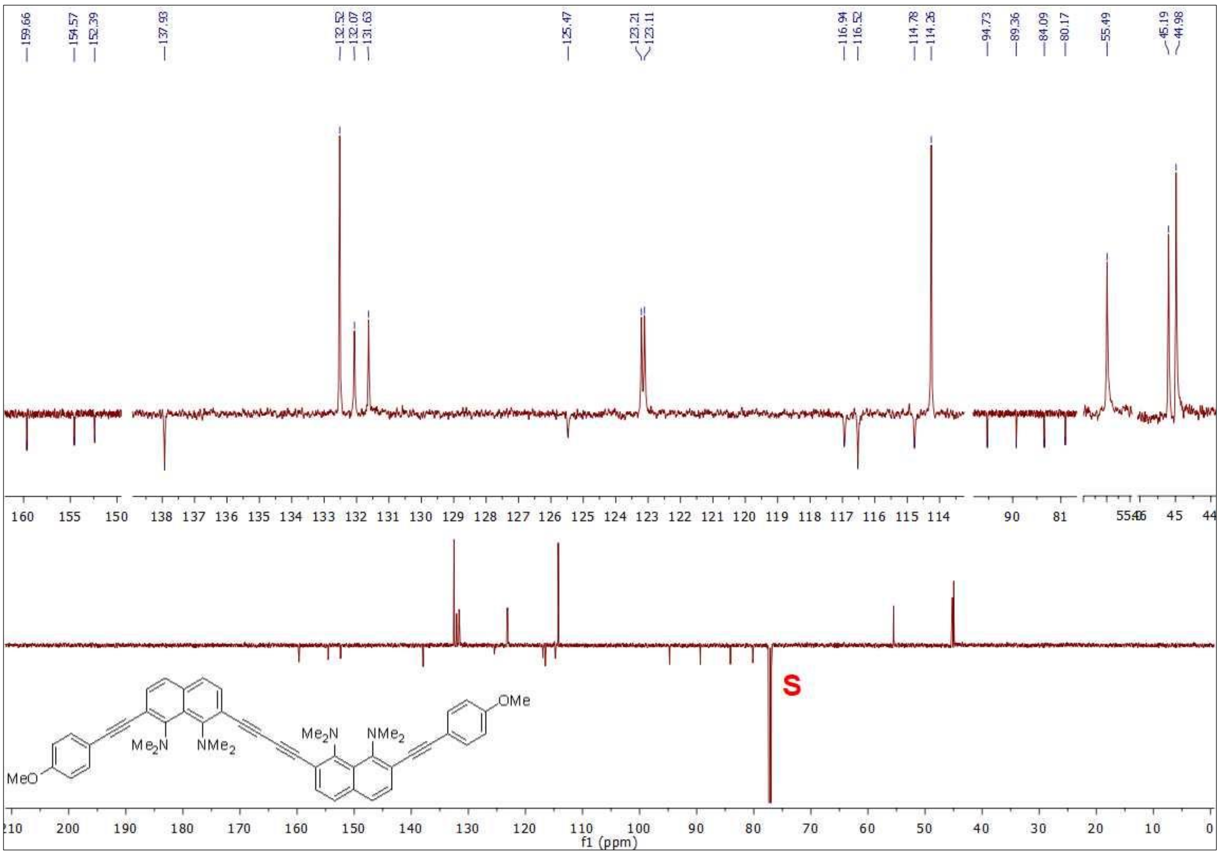

Figure S42. $^{13}C\{^{1}H\}$ APT-NMR spectrum of compound **5b** (150 MHz, $CDCl_3$).

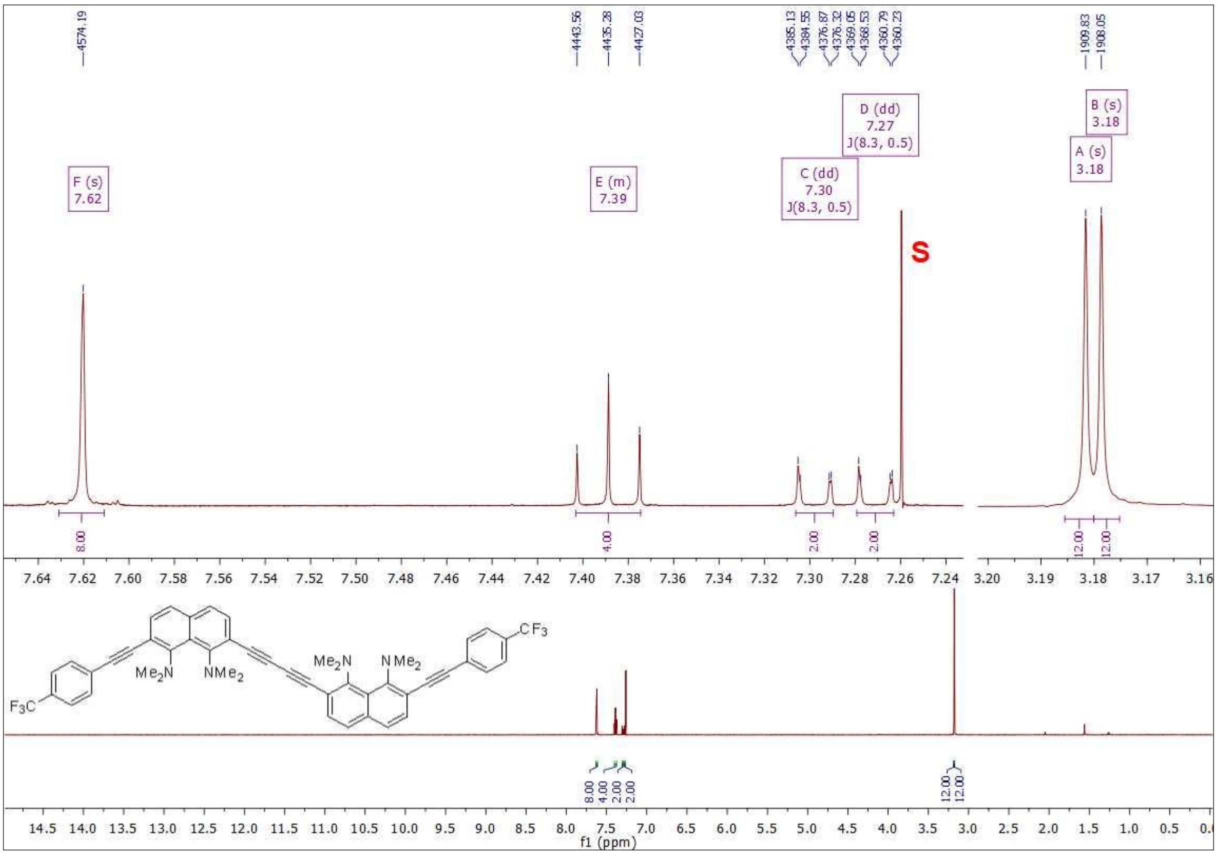

Figure S43. ^{1}H NMR spectrum of compound **5c** (600 MHz, $CDCl_3$).

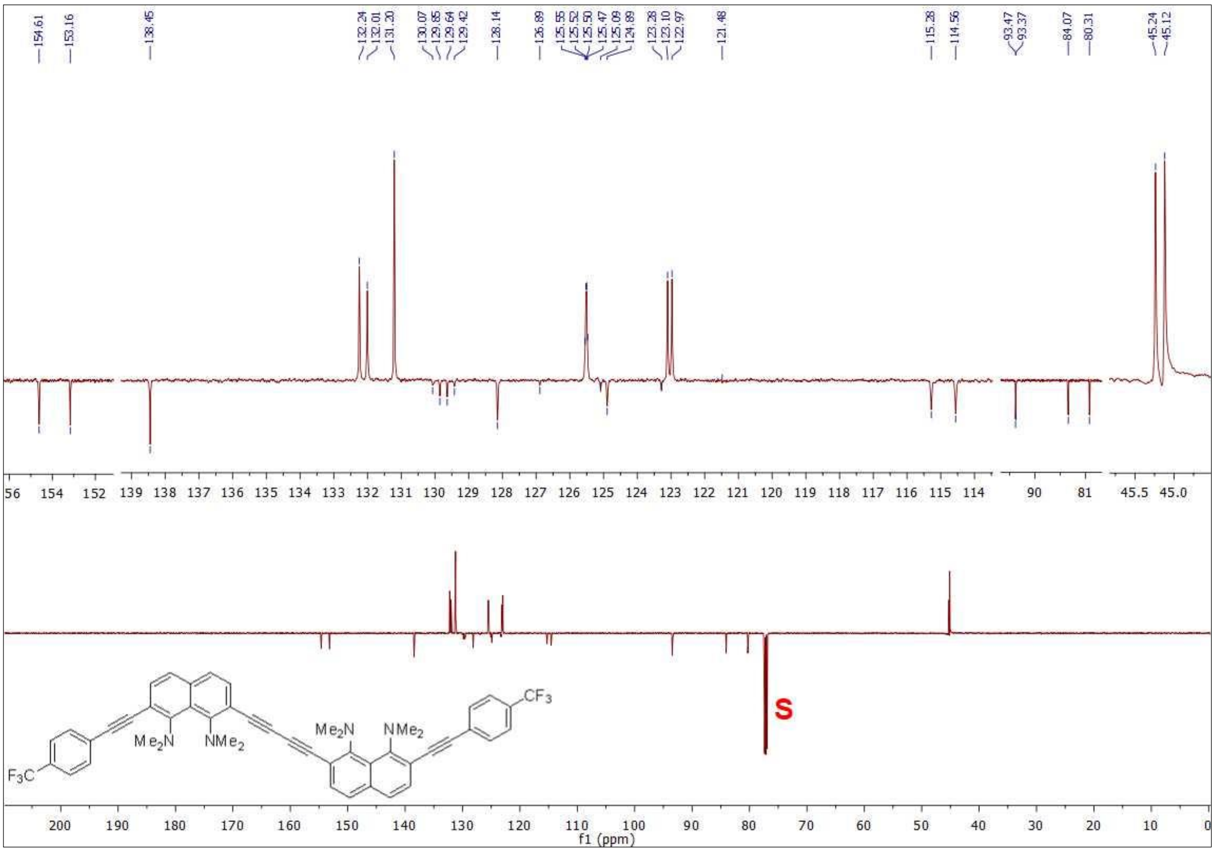

Figure S44. $^{13}C\{^{1}H\}$ APT-NMR spectrum of compound **5c** (150 MHz, $CDCl_3$).

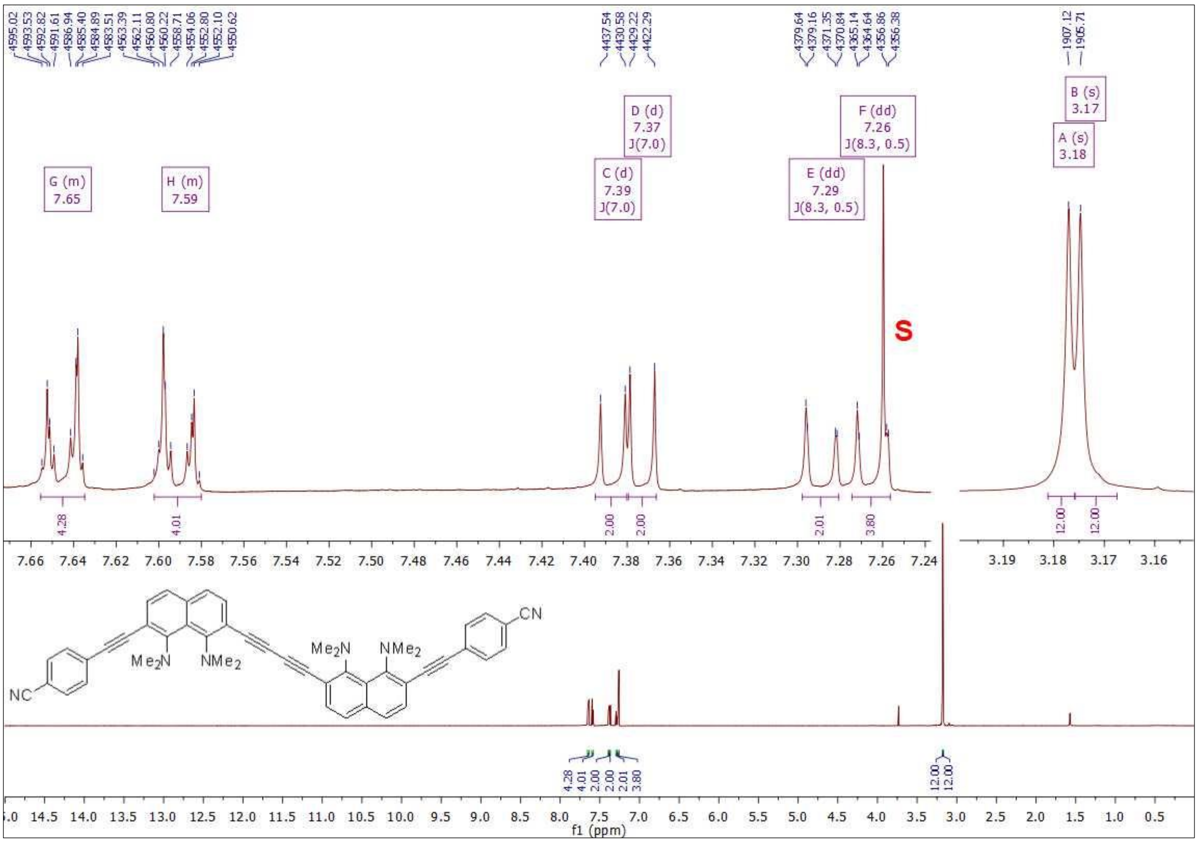

Figure S45. ^{1}H NMR spectrum of compound **5d** (600 MHz, $CDCl_3$).

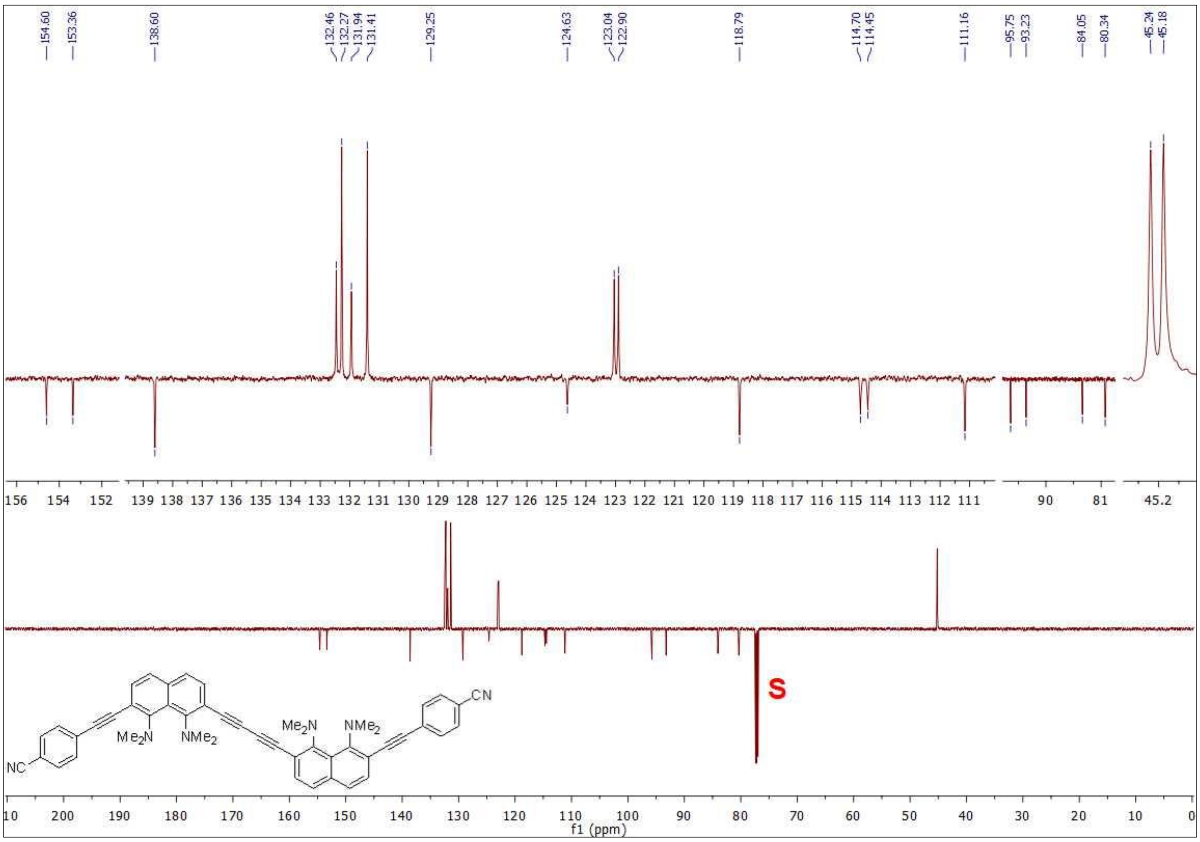

Figure S46. $^{13}C\{^{1}H\}$ APT-NMR spectrum of compound **5d** (150 MHz, $CDCl_3$).

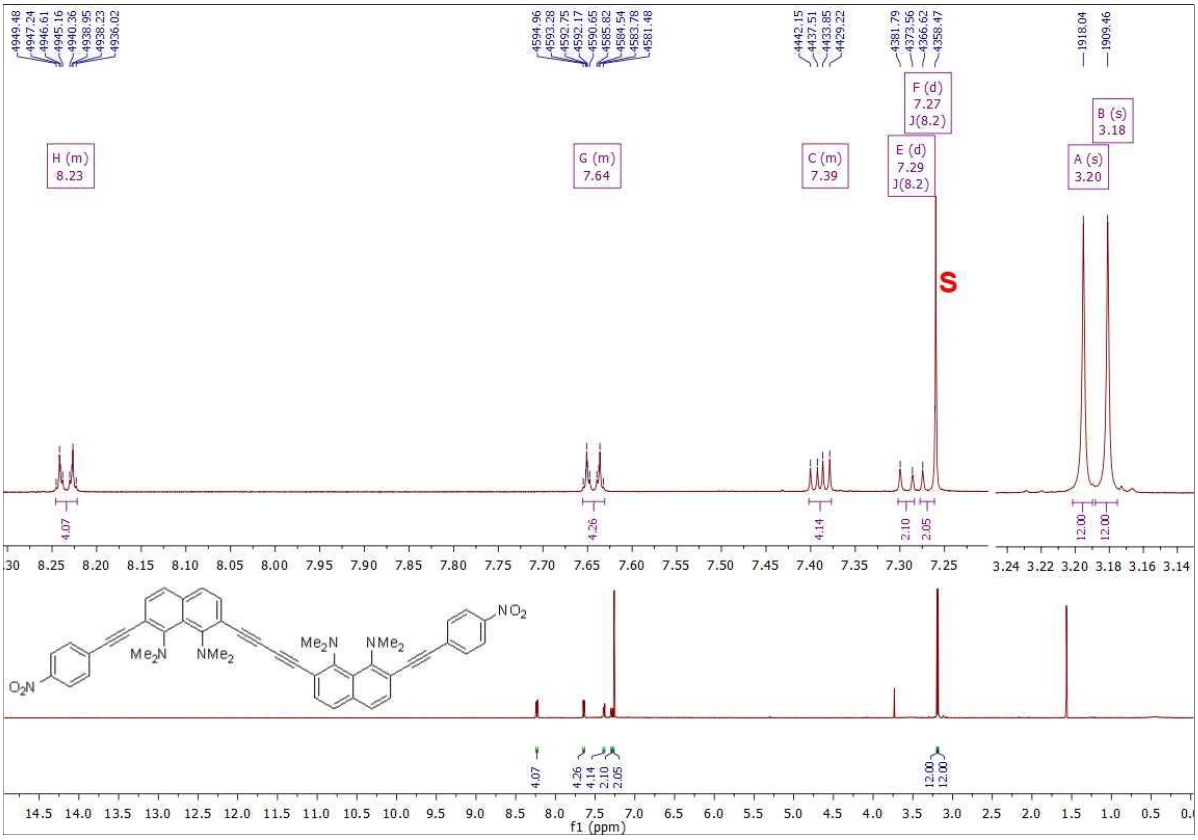

Figure S47. ^{1}H NMR spectrum of compound **5e** (600 MHz, $CDCl_3$).

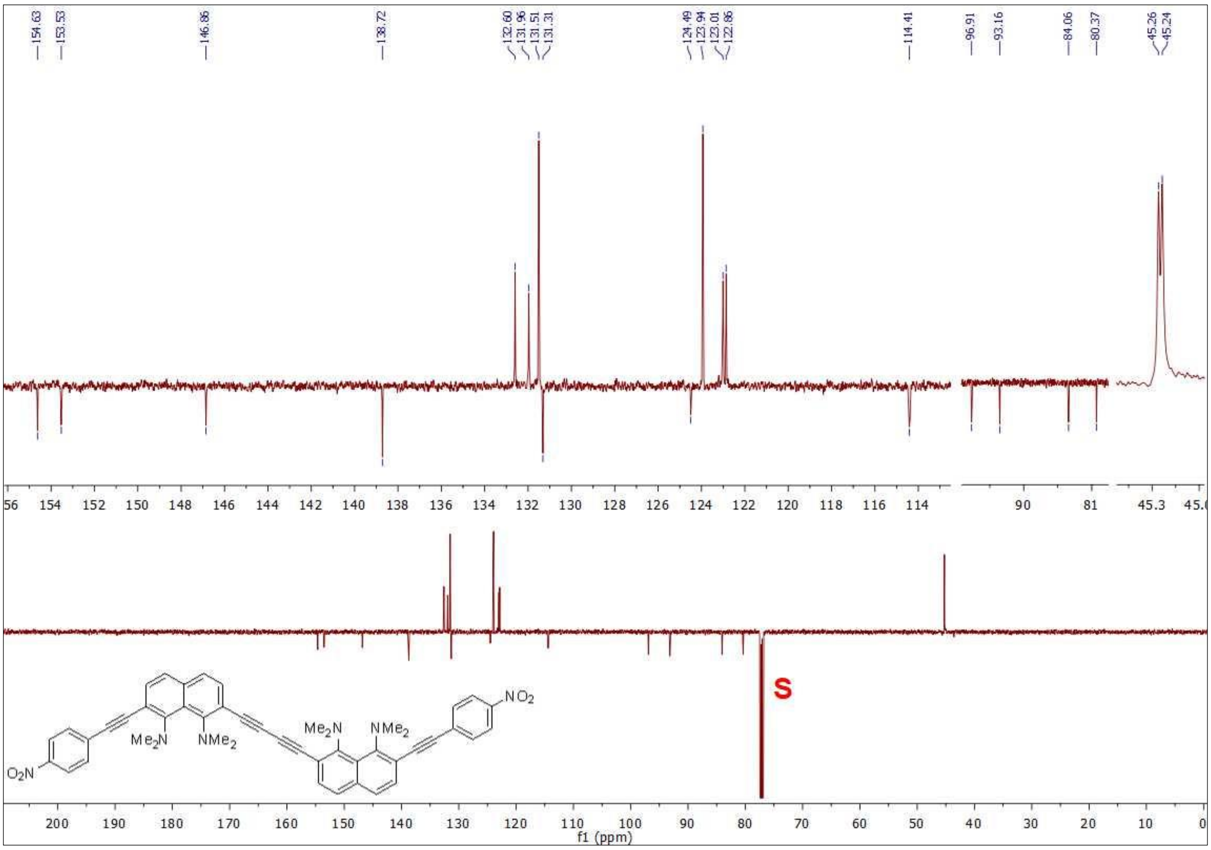

Figure S48. $^{13}C\{^1H\}$ APT-NMR spectrum of compound **5e** (150 MHz, $CDCl_3$).

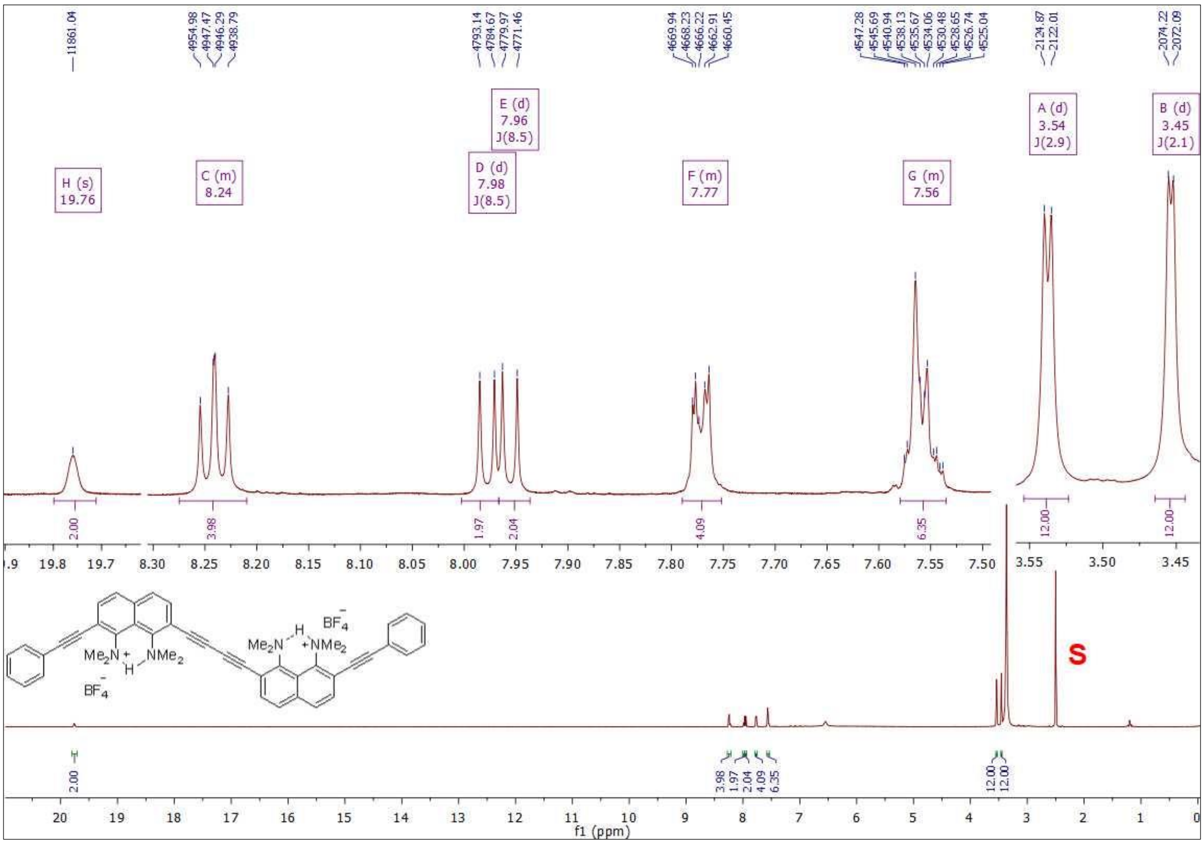

Figure S49. ^{1}H NMR spectrum of compound **11a** (600 MHz, DMSO-d$_6$).

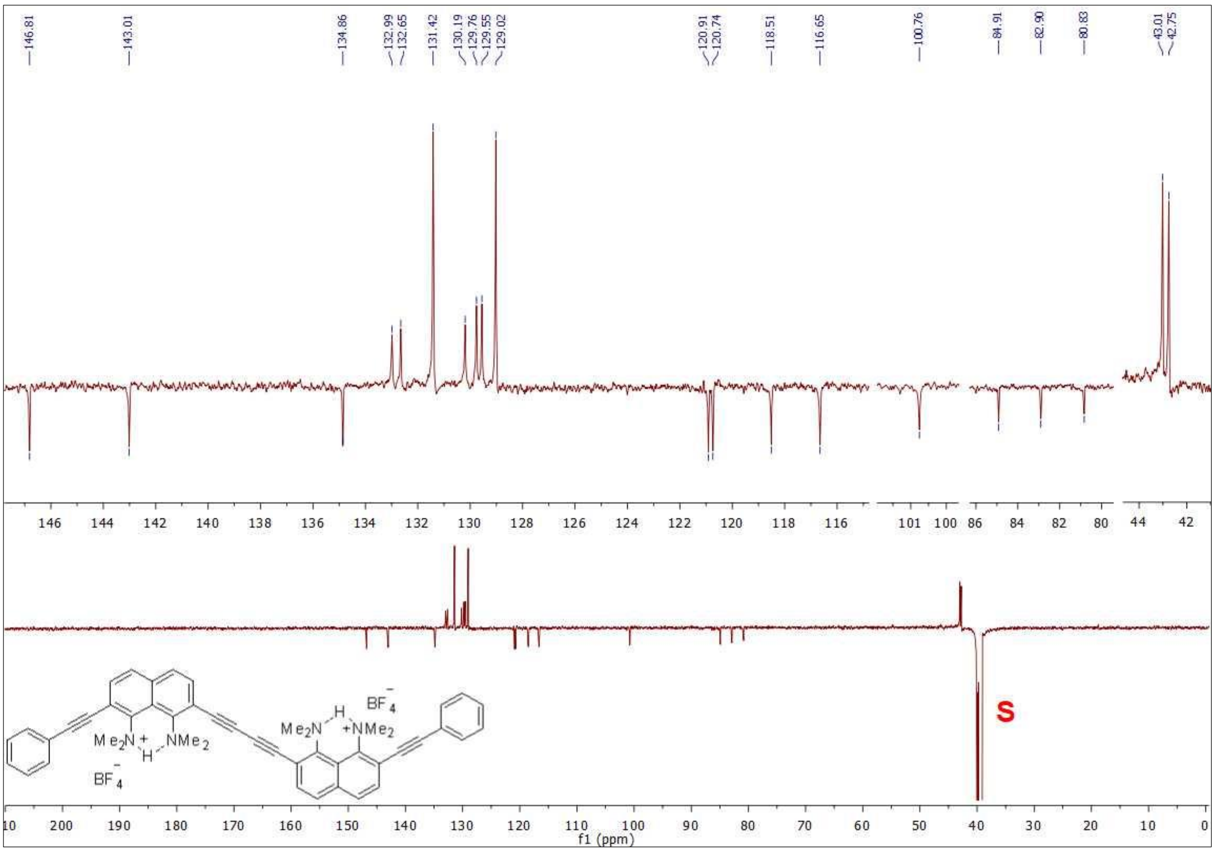

Figure S50. ^{13}C{^{1}H} APT-NMR spectrum of compound **11a** (150 MHz, DMSO-d$_6$).

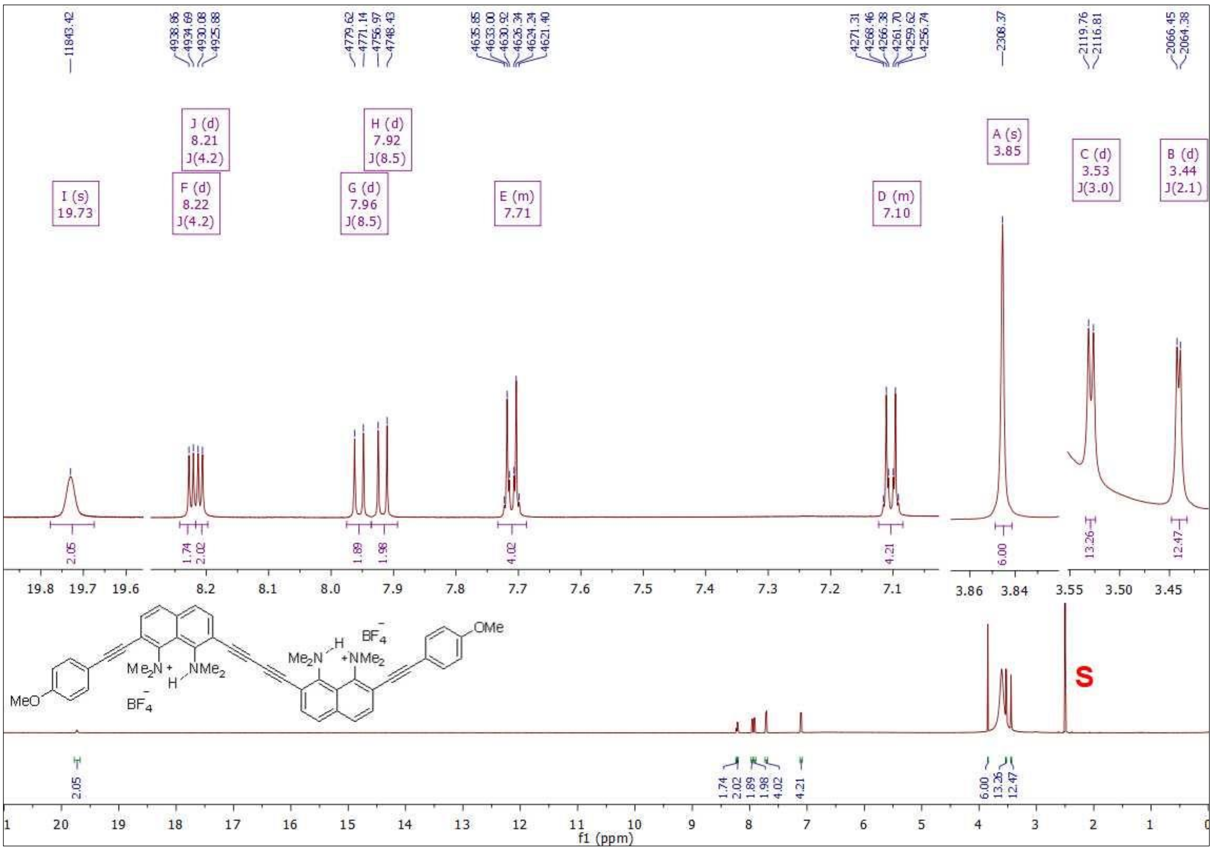

Figure S51. ^{1}H NMR spectrum of compound **11b** (600 MHz, DMSO-d_6).

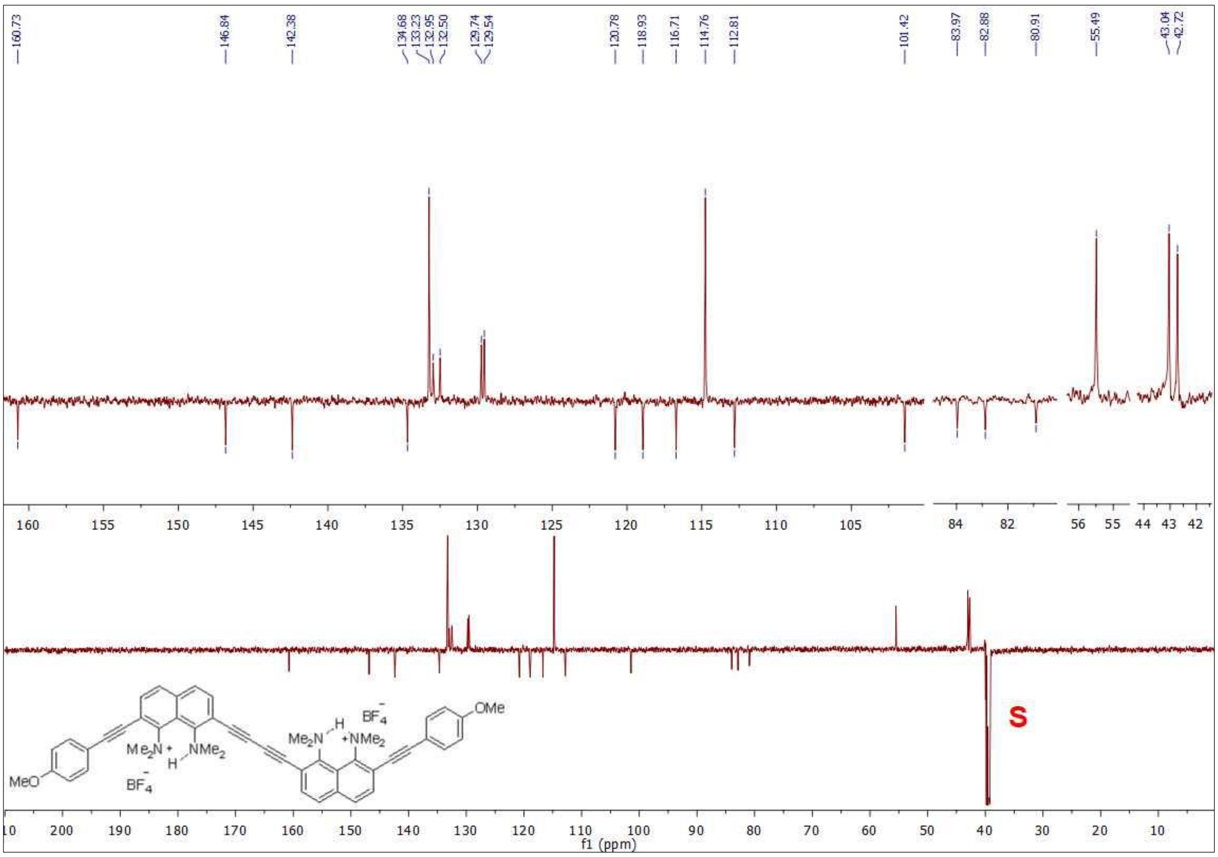

Figure S52. $^{13}C\{^{1}H\}$ APT-NMR spectrum of compound **11b** (150 MHz, DMSO-d_6).

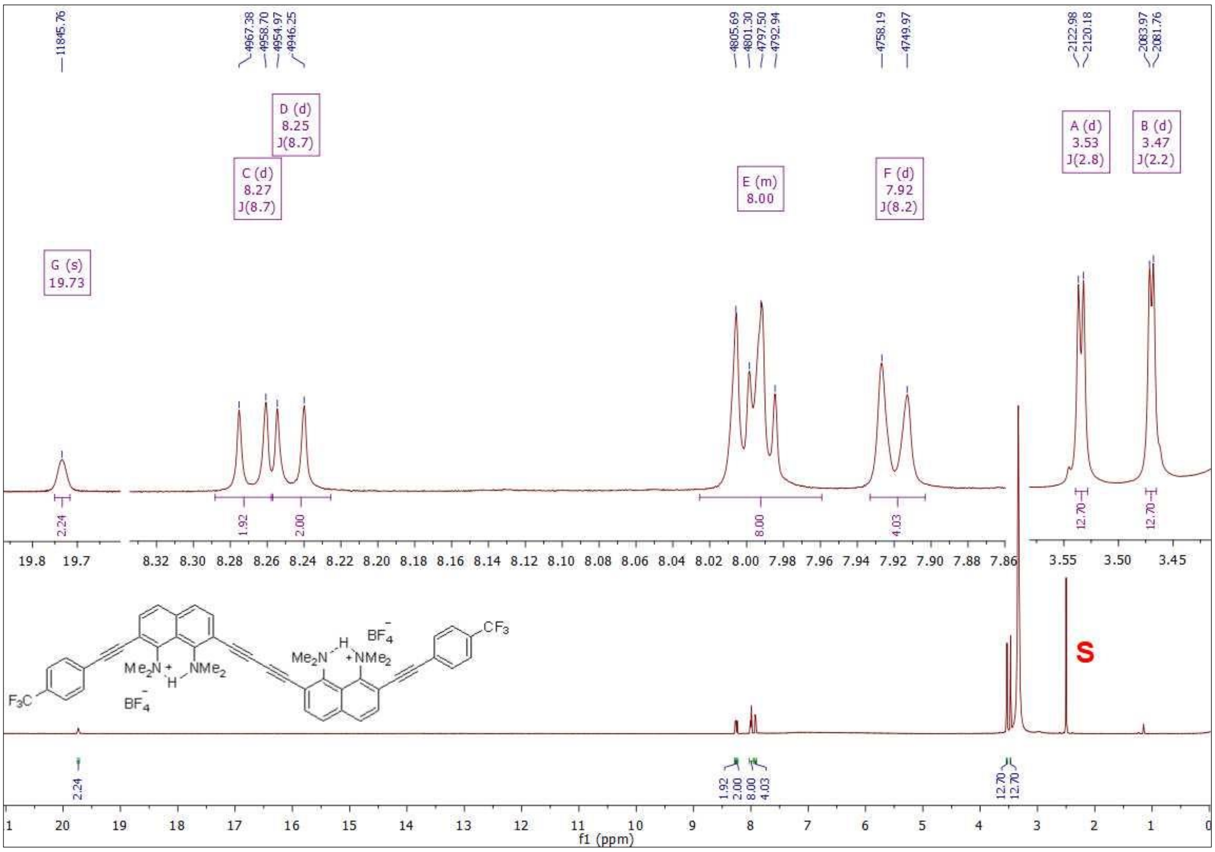

Figure S53. ^{1}H NMR spectrum of compound **11c** (600 MHz, DMSO-d_6).

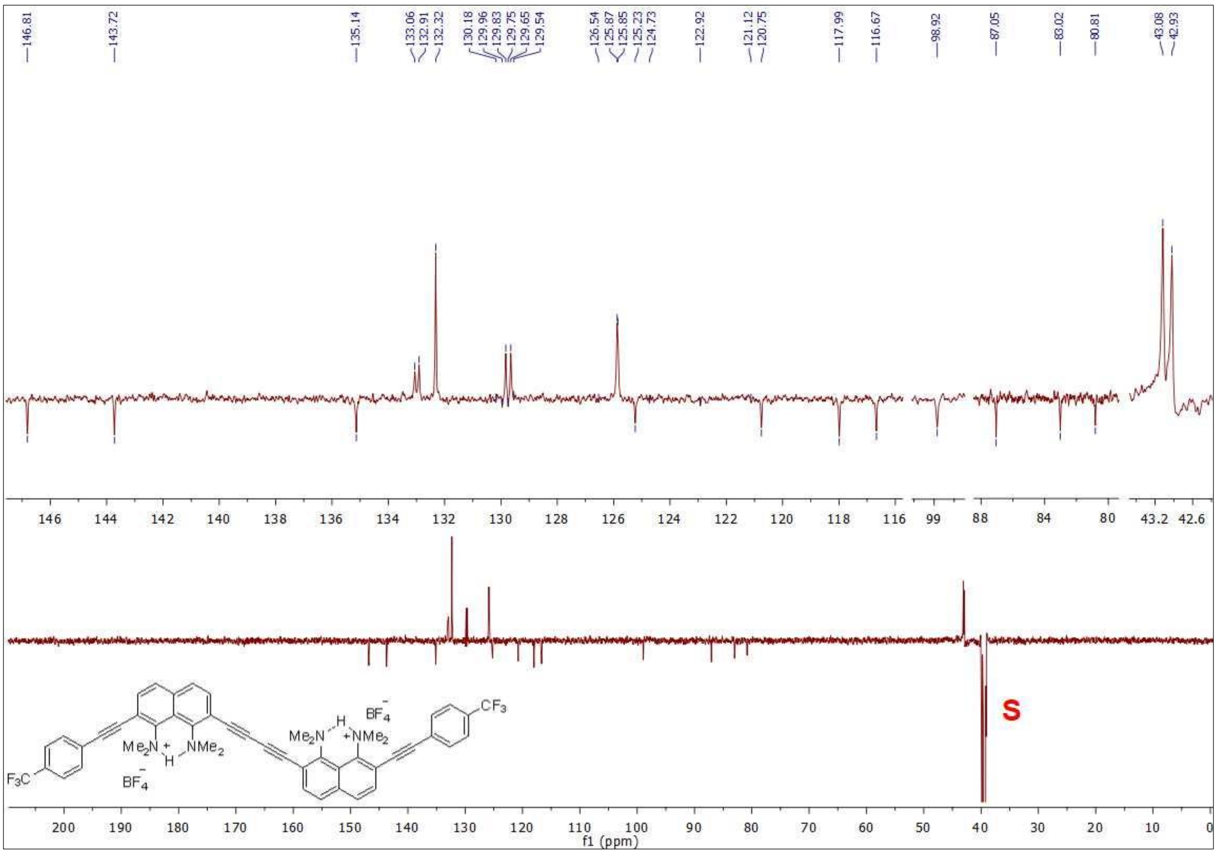

Figure S54. ^{13}C{^{1}H} APT-NMR spectrum of compound **11c** (150 MHz, DMSO-d_6).

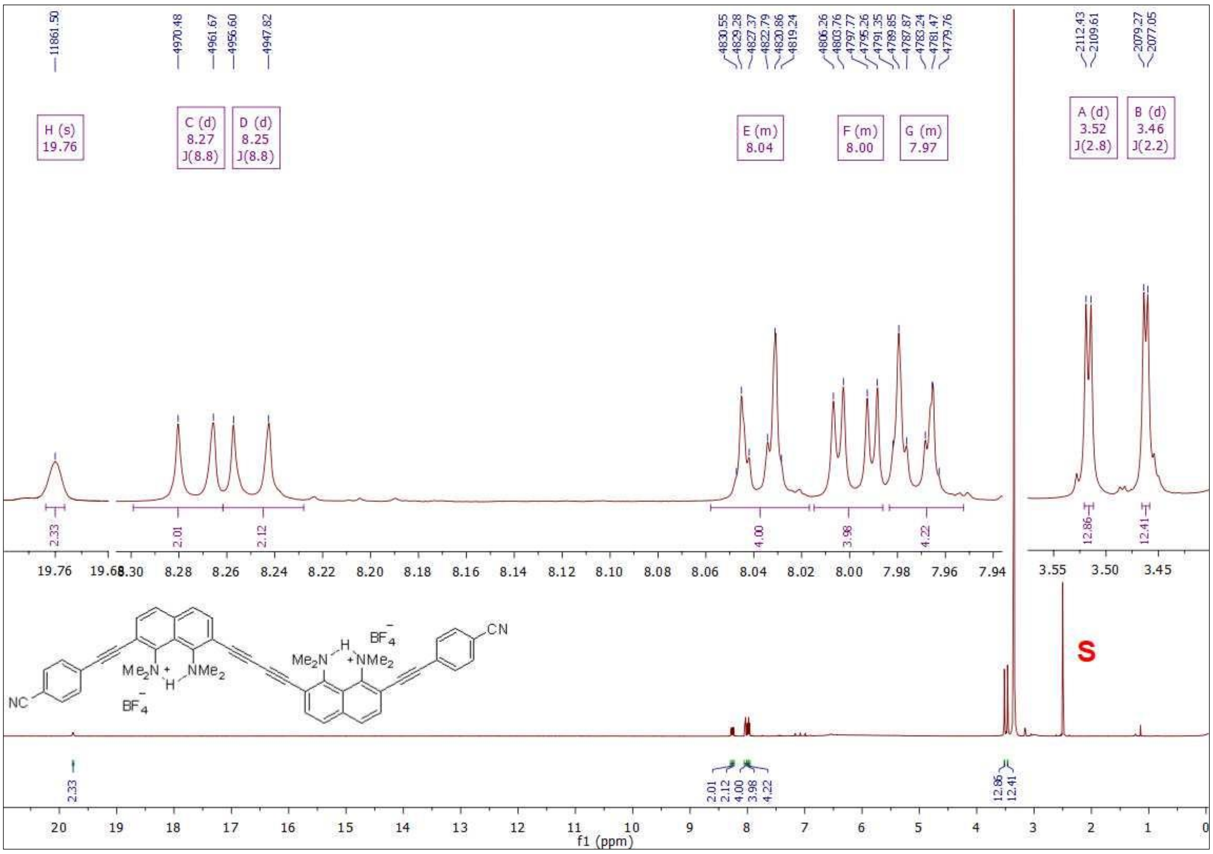

Figure S55. ^{1}H NMR spectrum of compound **11d** (600 MHz, DMSO-d$_6$).

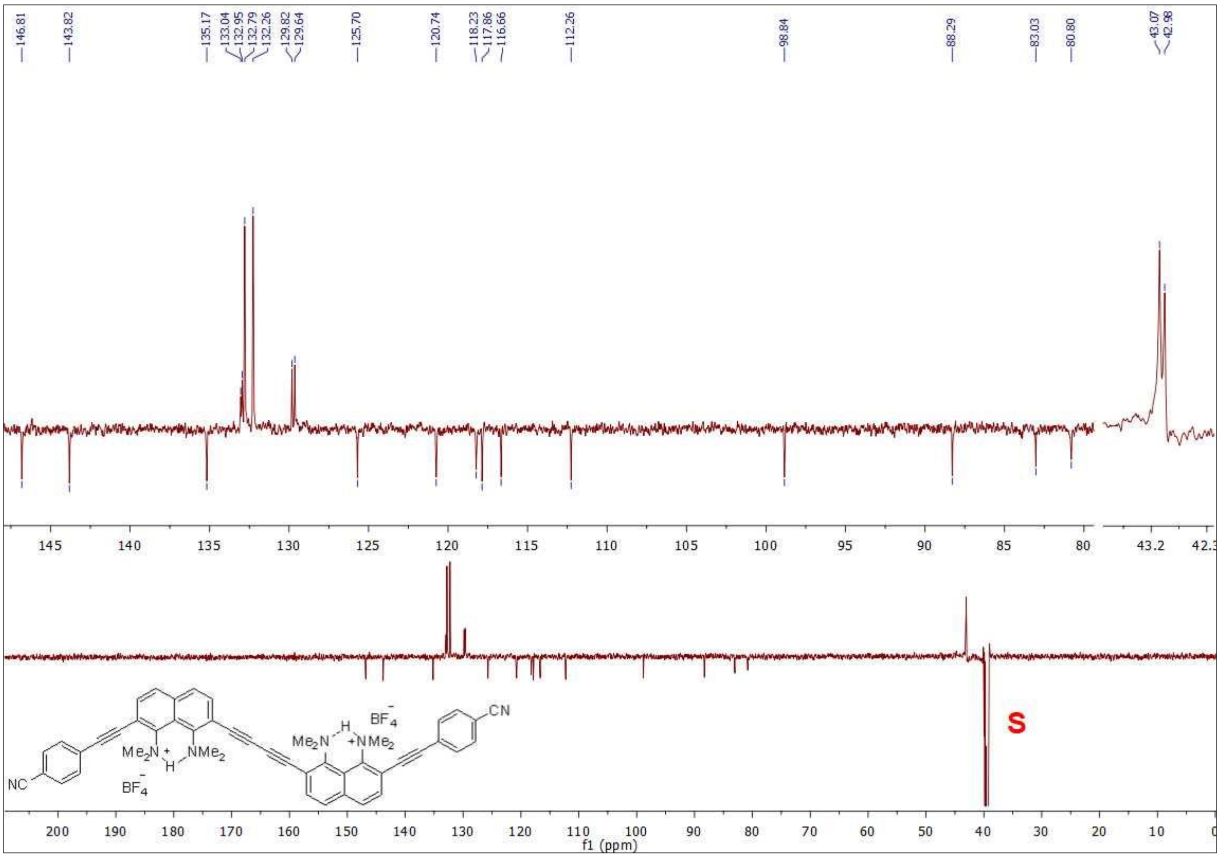

Figure S56. ^{13}C{^{1}H} APT-NMR spectrum of compound **11d** (150 MHz, DMSO-d$_6$).

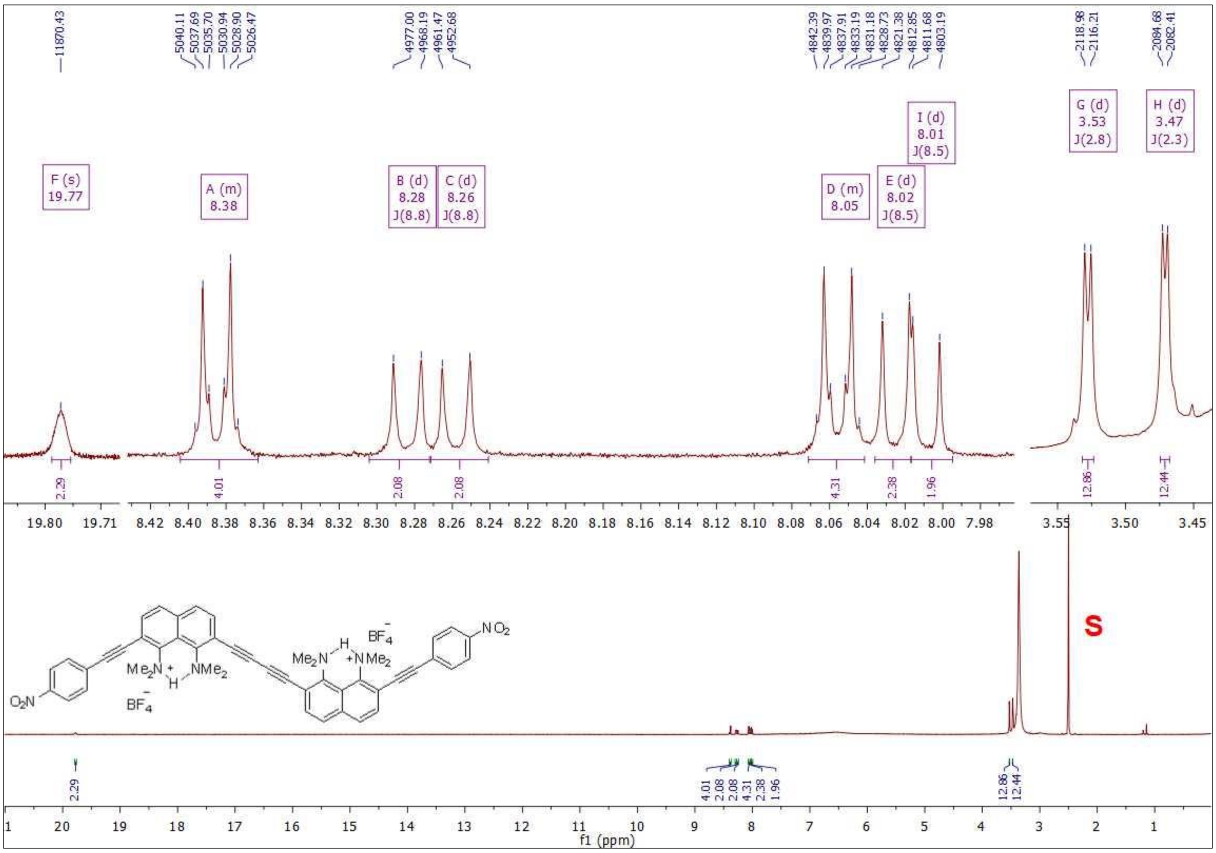

Figure S57. ^{1}H NMR spectrum of compound **11e** (600 MHz, DMSO-d_6).

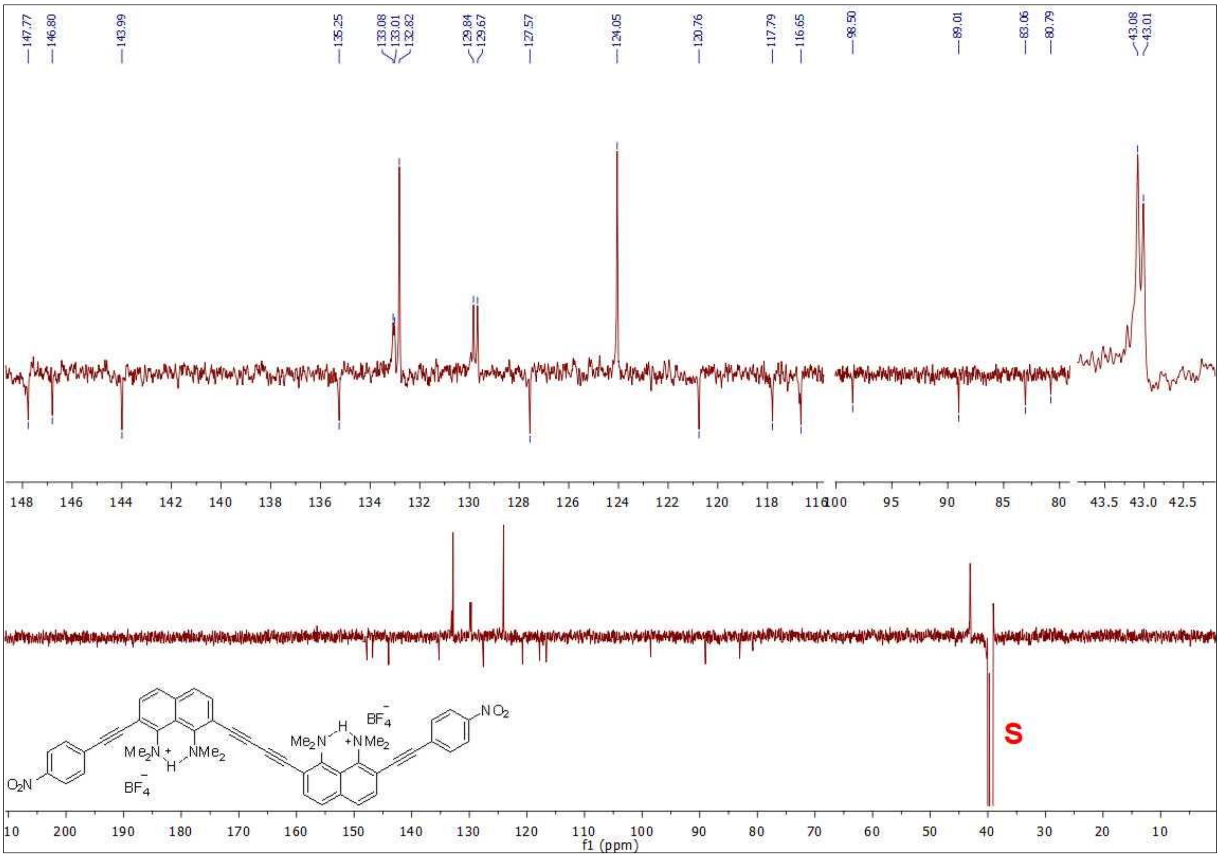

Figure S58. $^{13}C\{^{1}H\}$ APT-NMR spectrum of compound **11e** (150 MHz, DMSO-d_6).

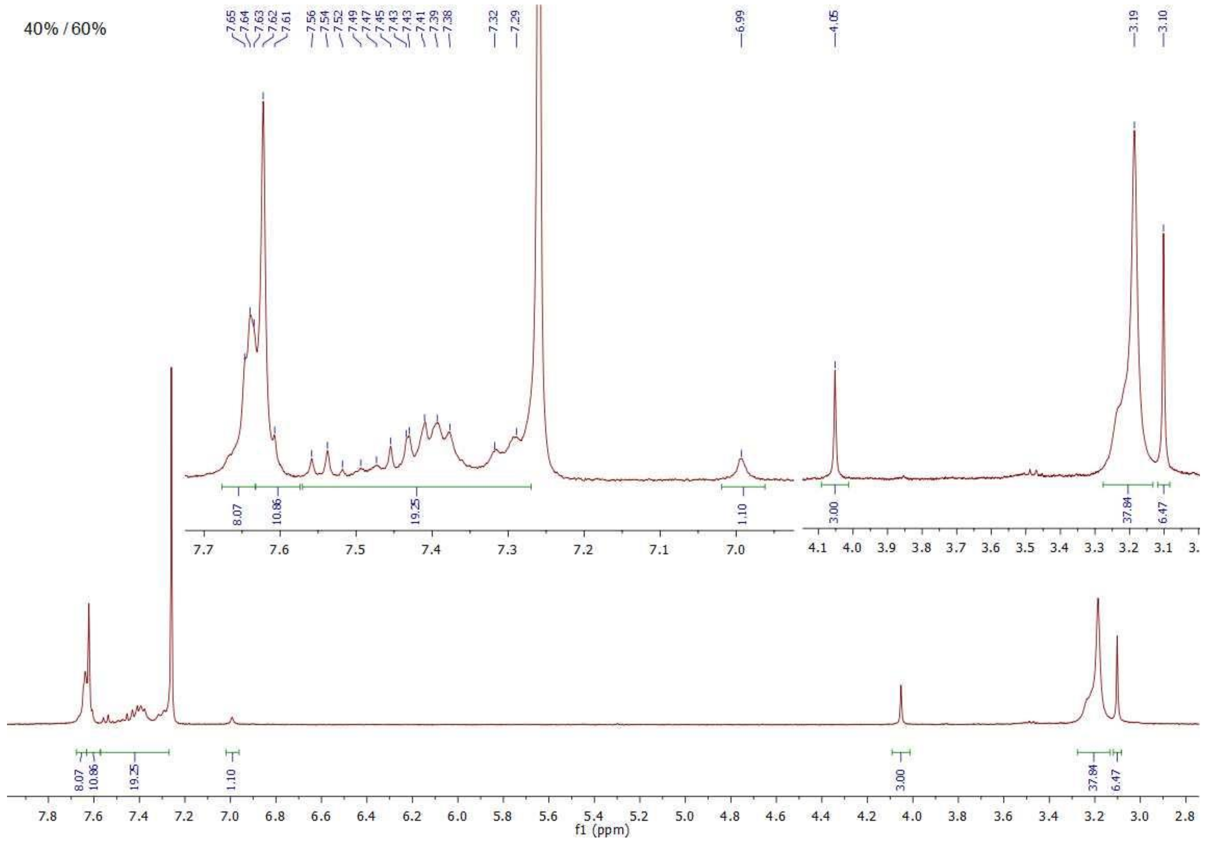

Figure S59. ^{1}H NMR spectrum of a mixture of compounds **5c** and **12** formed after keeping a solution of **5c** in chloroform/ethanol 1:1 for a month (250 MHz, $CDCl_3$).

X-ray data

Crystal structure determination: X-Ray measurements were conducted with four-circle SuperNova and XtaLAB Synergy diffractometers, single source at offset/far, AtlasS2 and HyPix3000 detectors (CuK_α radiation). Atomic coordinates, bond lengths, bond angles and thermal parameters have been deposited at the Cambridge Crystallographic Data Centre (CCDC) and allocated the deposition numbers CCDC 2215907 (**5b**), CCDC 2215905 (**5d**), CCDC 2215904 (**5e**), CCDC 2215908 (**9e**), CCDC 2215909 (**11c**) and CCDC 2215906 (**12**). These data can be obtained free of charge from the Cambridge Crystallographic Data Centre via www.ccdc.cam.ac.uk/data_request/cif.

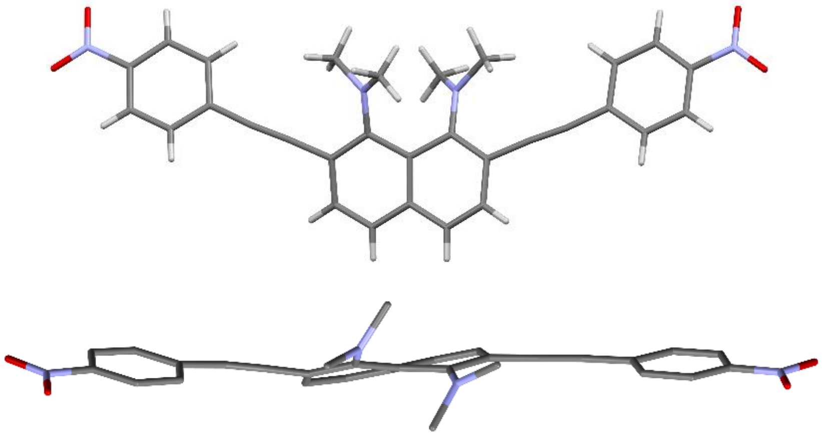

Figure S60. Molecular structure of compound **9e:** frontal (top) and side views (bottom, hydrogen atoms omitted).

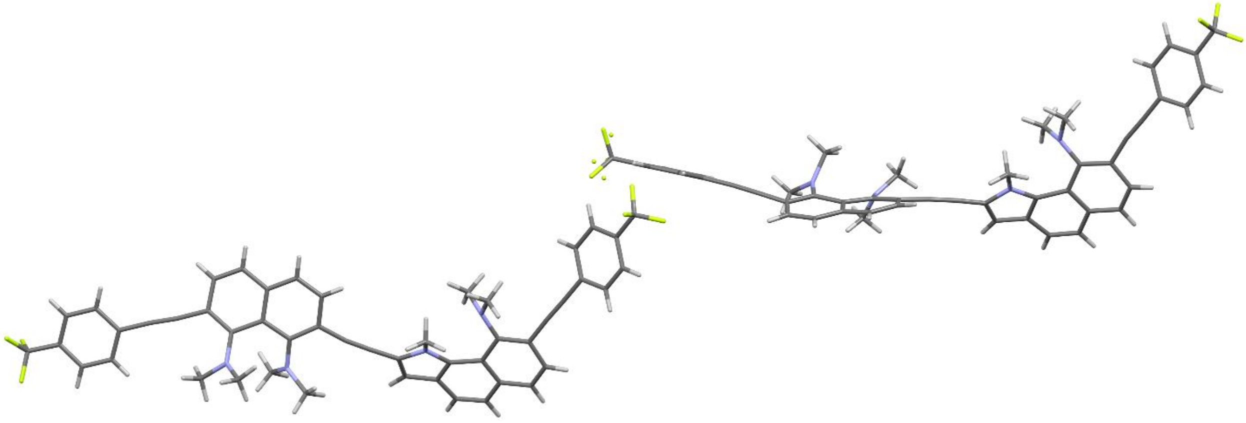

Figure S61. Molecular structure of compound **12** (two independent molecules in the unit cell).

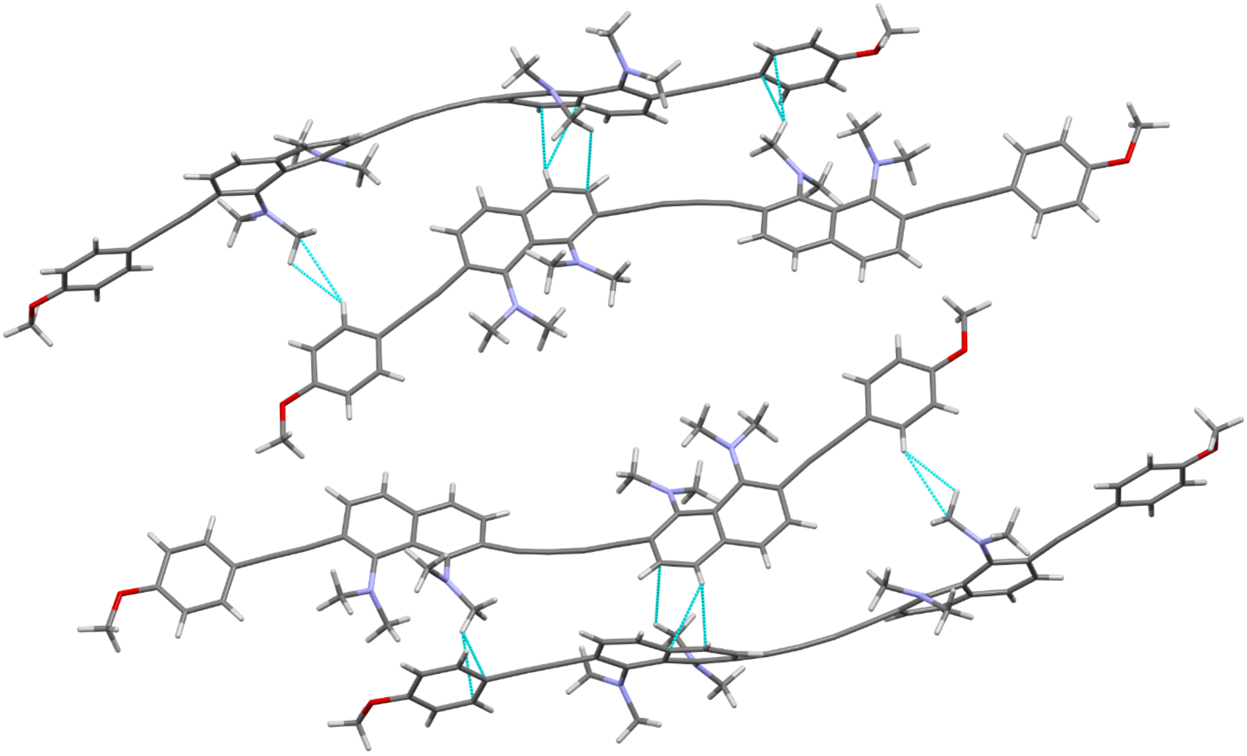

Figure S62. Short contacts in the unit cell of **5b**.

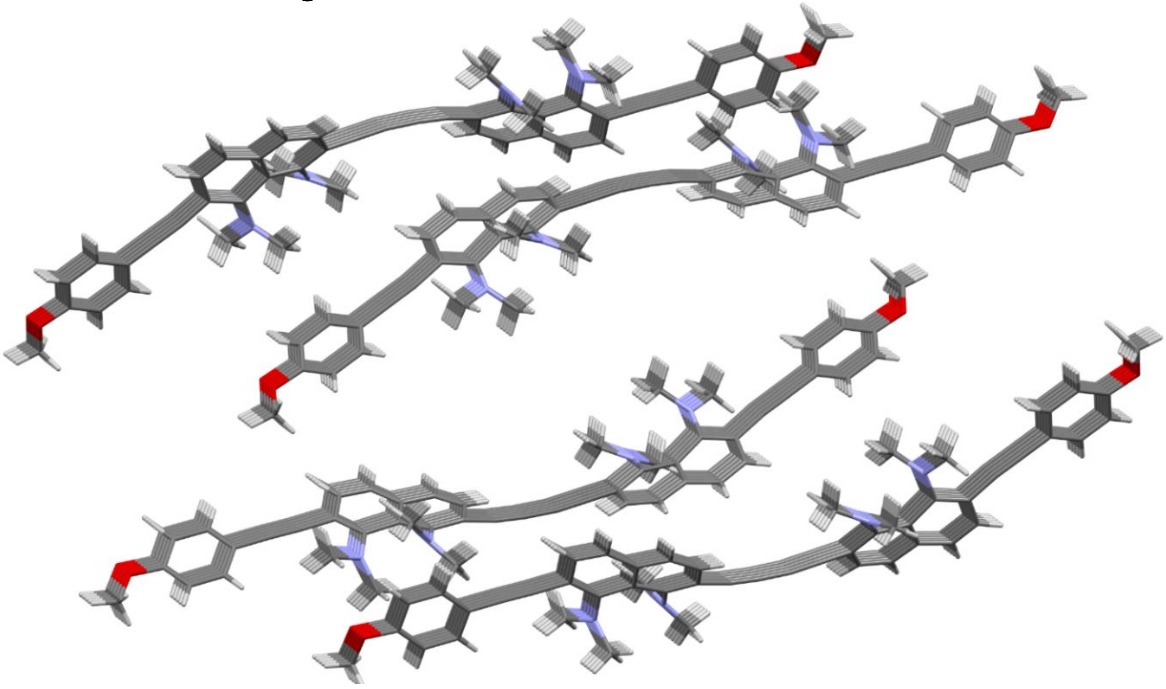

Figure S63. Crystal packing of **5b** (view along *b* axis).

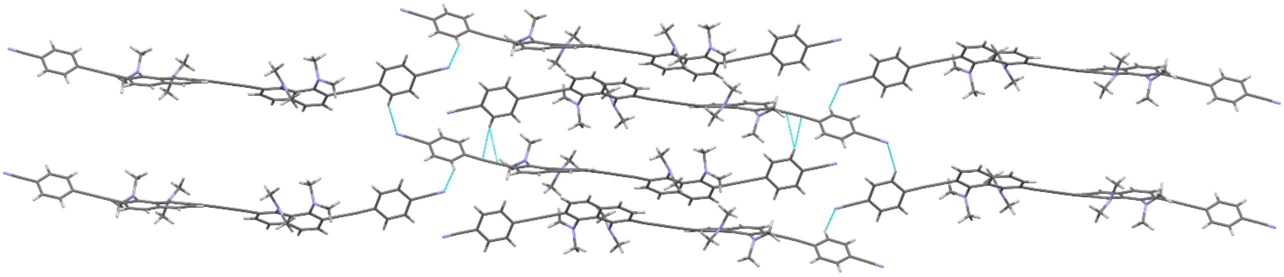

Figure S64. Crystal packing of **5d** (short contacts).

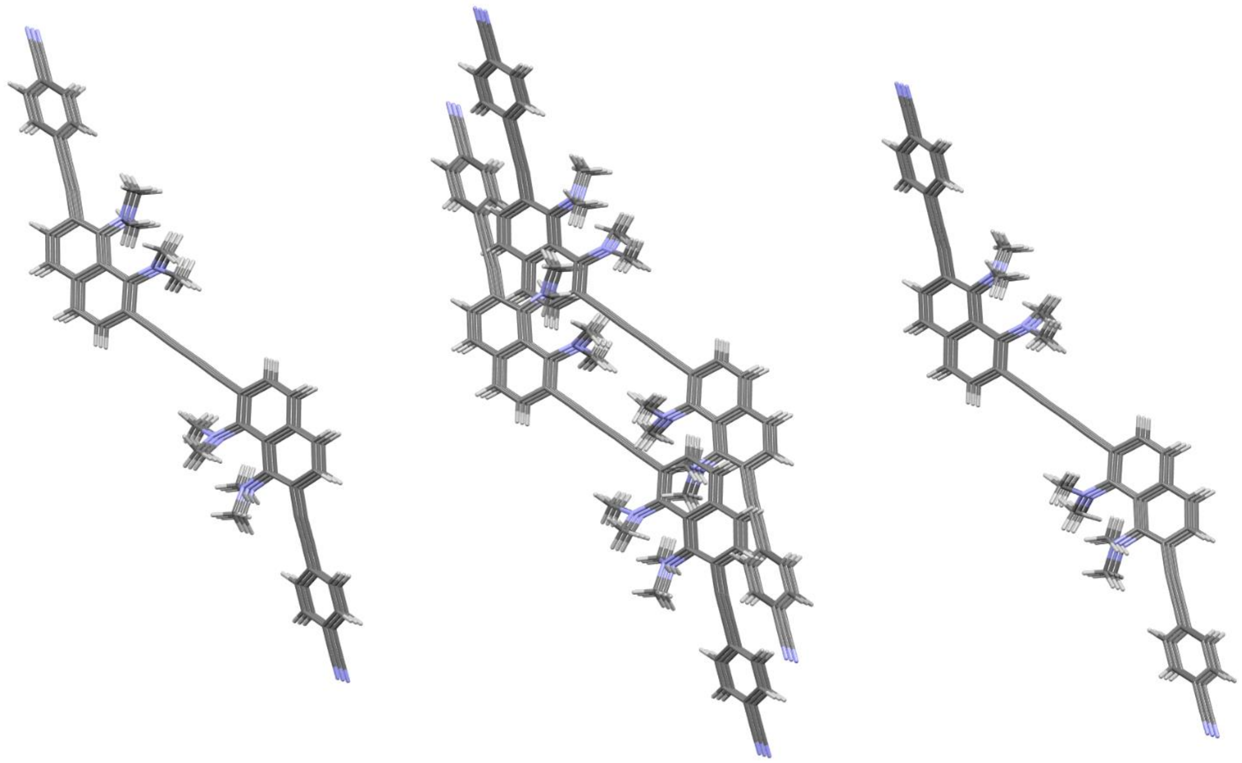

Figure S65. Crystal packing of **5d** (view along *b* axis).

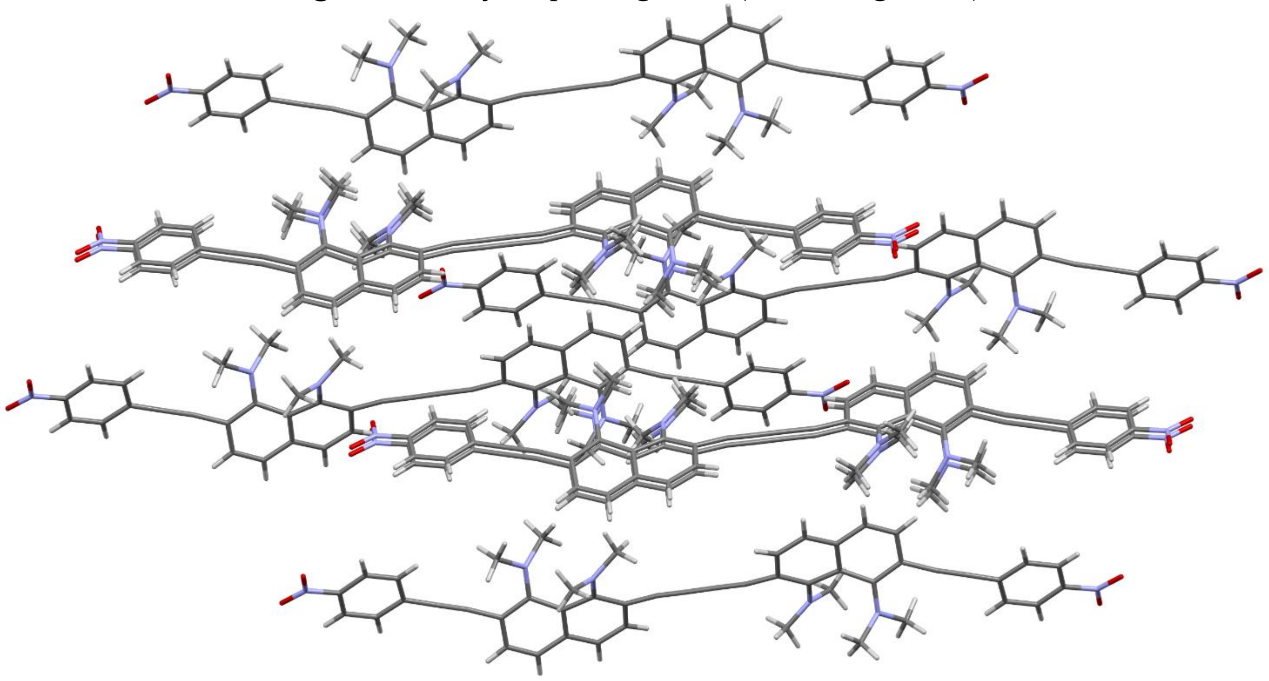

Figure S66. Crystal packing of **5e** (view along *b* axis).

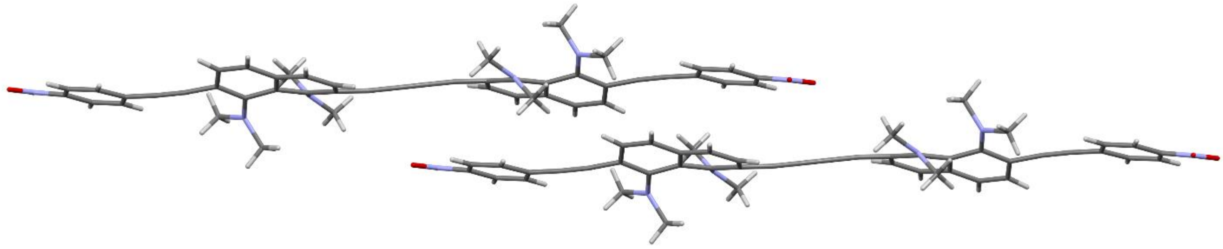

Figure S67. Molecules **5e** tend to approach π-donor DMAN and π-acceptor *p*-nitrophenyl fragments with the shortest distance between the two molecules equal to 2.810 Å.

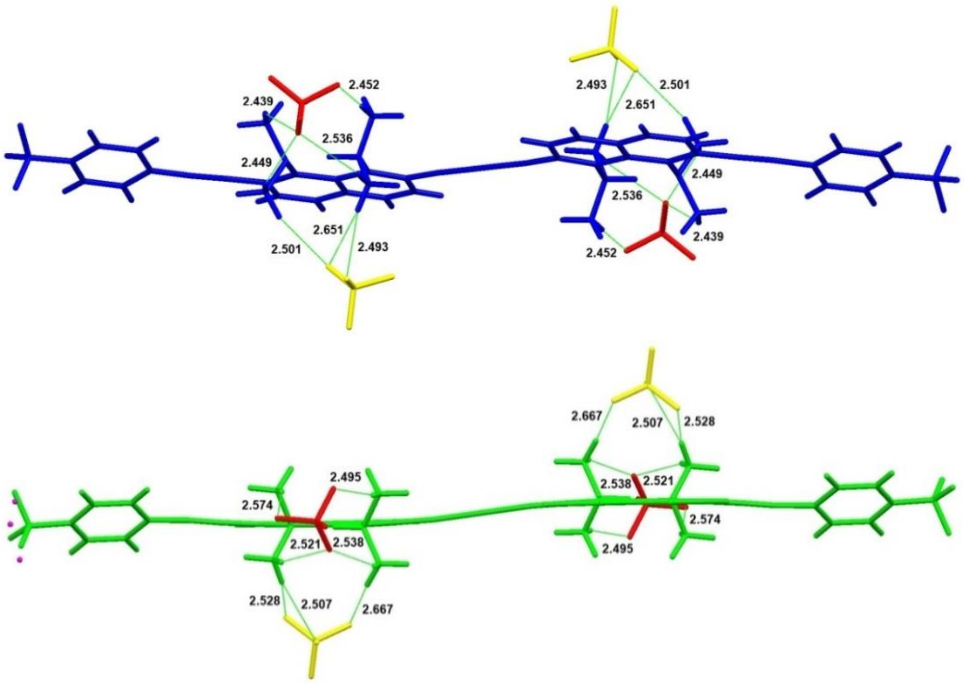

Figure S68. Two types of independent non-equivalent dications, marked in blue and green, and two types of BF_4^- anions, marked in red and yellow, in the crystal structure of salt **11c**.

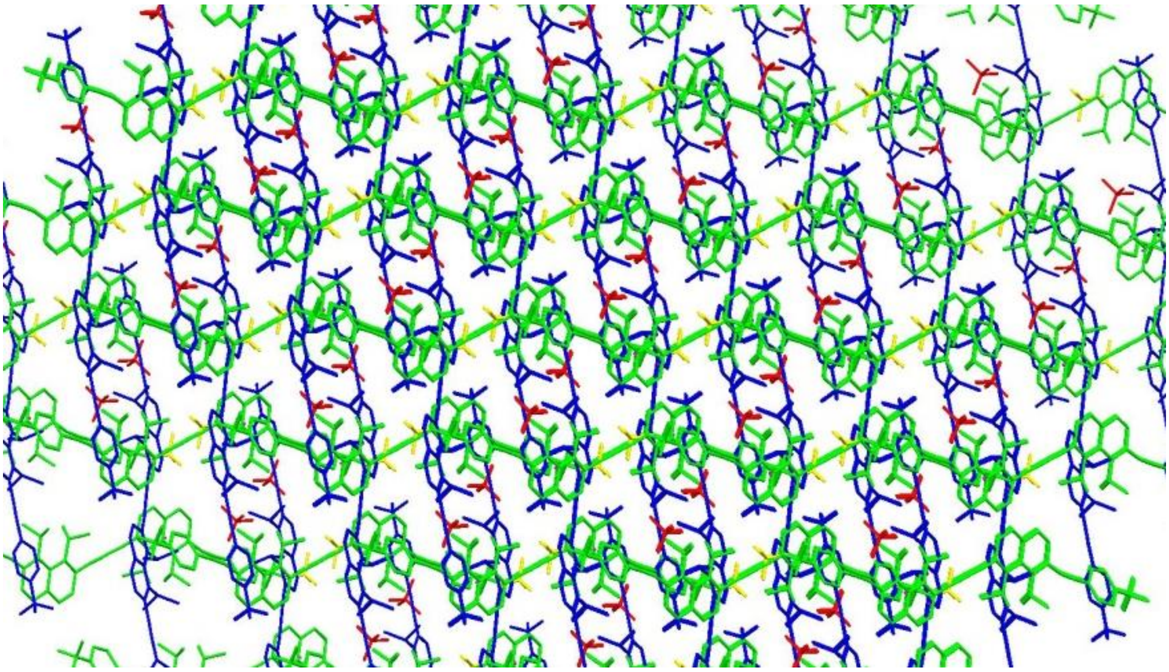

Figure S69. Crystal packing of salt **11c** with perpendicular layers of cations of two types.

References

[1] A. F. Pozharskii, O. V. Ryabtsova, V. A. Ozeryanskii, A. V. Degtyarev, O. N. Kazheva, G. G. Alexandrov, O. A. Dyachenko, *J. Org. Chem*. **2003**, *68*, 10109–10122.

[2] W. Chen, B. Wang, N. Liu, D. Huang, X. Wang, Y. Hu, *Org. Lett*. **2014**, *16*, 6140–6143.

[3] E. A. Filatova, A. F. Pozharskii, A. V. Gulevskaya, N. V. Vistorobskii, V. A. Ozeryanskii, *Synlett*, **2013**, *24*, 2515–2518.
